# Design, Synthesis, and in Vitro Evaluation of 4‐(4‐Hydroxyphenyl)piperazine‐Based Compounds Targeting Tyrosinase

**DOI:** 10.1002/cmdc.202200305

**Published:** 2022-09-26

**Authors:** Salvatore Mirabile, Maria Paola Germanò, Antonella Fais, Lisa Lombardo, Federico Ricci, Sonia Floris, Anna Cacciola, Antonio Rapisarda, Rosaria Gitto, Laura De Luca

**Affiliations:** ^1^ Department of Chemical, Biological, Pharmaceutical, and Environmental Sciences University of Messina Viale Stagno D'Alcontres 31, Pole Papardo 98166 Messina Italy; ^2^ Foundation Prof. Antonio Imbesi University of Messina Piazza Pugliatti 1 98122 Messina Italy; ^3^ Department of Life and Environment Sciences University of Cagliari 09042 Monserrato Cagliari Italy

**Keywords:** *Agaricus bisporus*, Docking studies, Tyrosinase inhibitors, B16F10 melanoma cells, Anti-melanogenic effects

## Abstract

Melanin biosynthesis is enzymatically regulated by tyrosinase (TYR, EC 1.14.18.1), which is efficiently inhibited by natural and synthetic phenols, demonstrating potential therapeutic application for the treatment of several human diseases. Herein we report the inhibitory effects of a series of (4‐(4‐hydroxyphenyl)piperazin‐1‐yl)arylmethanone derivatives, that were designed, synthesised and assayed against TYR from *Agaricus bisporus* (AbTYR). The best inhibitory activity was predominantly found for compounds bearing selected hydrophobic *ortho*‐substituents on the aroyl moiety (IC_50_ values in the range of 1.5–4.6 μM). They proved to be more potent than the reference compound kojic acid (IC_50_=17.8 μM) and displayed competitive mechanism of inhibition of diphenolase activity of AbTYR. Docking simulation predicted their binding mode into the catalytic cavities of AbTYR and the modelled human TYR. In addition, these compounds displayed antioxidant activity combined with no cytotoxicity in MTT tests. Notably, the best inhibitor affected tyrosinase activity in α‐MSH‐stimulated B16F10 cells, thus demonstrating anti‐melanogenic activity.

## Graphical abstract

Anti‐melanogenic agents: The design, synthesis, docking and inhibitory assays toward tyrosinase (TYR) from *Agaricus bisporus* (AbTYR) were performed for a series of (4‐(4‐hydroxyphenyl)piperazin‐1‐yl)arylmethanone derivatives. The most promising compound 10 displayed antioxidant activity combined with no cytotoxicity in MTT tests and exerted anti‐melanogenic effects on B16F10 cells.

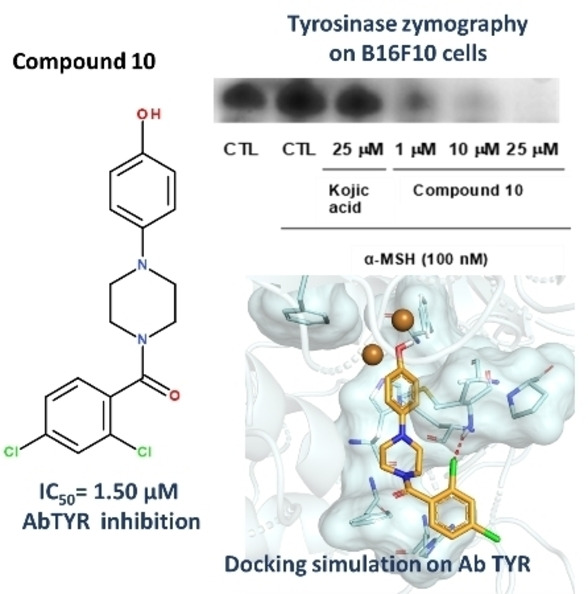

## Introduction

Tyrosinase (TYR, E.C. 1.14.18.1) is a type‐3 binuclear copper oxidoreductase involved in mammalian melanogenesis, that controls pigmentation of retina and skin. The melanin synthesis proceeds through different steps; the catalytic cycle consists in the oxidation of monophenols and then diphenols in presence of oxygen, thus generating *o*‐benzoquinone derivative leading to melanin synthesis through different enzymatic reactions accelerated by TYRP‐1 (Tyrosinase related protein‐1) and TYRP‐2 (Tyrosinase related protein‐2).^[1]^ TYR activity contributes to the key rate‐limiting reaction in melanin production.^[2]^ The upregulated TYR oxidation lead to the excessive accumulation of melanin, which is associated to skin pigmentation related to human diseases (melasma, freckles, senile spots, malignant melanoma).[^3^, ^4^] Therefore, tyrosinase inhibitors (TYRIs) can reduce excessive melanin production in melanocytes and constitute promising therapeutic strategy for the treatment of hyperpigmentation diseases.

The human tyrosinase (hTYR) is a transmembrane protein that is highly glycosylated. hTYR is a membrane‐bound enzyme which is considered hard to purify and crystallize. Therefore, the wide screening campaign to identify TYRIs is generally carried out by employing the tetrameric TYR from *Agaricus bisporus* (AbTYR). It is well‐known that hTYR and AbTYR share only 23 % of similarity, however they show similar composition on catalytic sites, thus corroborating the employment of AbTYR as amenable and cheap surrogated of hTYR to perform preliminary biochemical screening.^[5]^ It has been demonstrated that several AbTYR inhibitors are poor active agents toward hTYR, therefore their potential anti‐melanogenic effects should be confirmed in human systems.[^2^, ^6^, ^7^]

**Figure 1:**
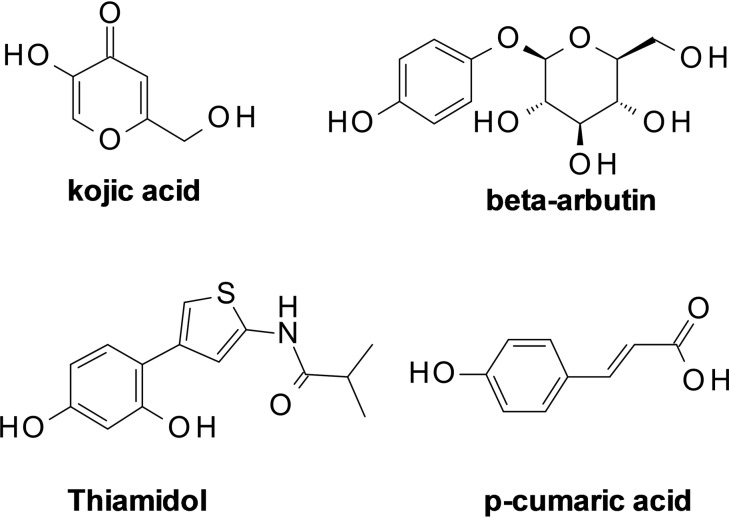
Chemical structures of selected TYRIs from natural and chemical sources bearing phenolic moiety.

To date several TYRIs had been identified; some of them are extracted or generated from natural sources.[^8^, ^9^] Ex‐novo synthesized TYRIs emerged from *in silico* approaches of drug discovery. Overall, TYRIs belong to distinct chemical classes including flavonoids,^[10]^ phenols,^[11]^ terpenes, miscellanea of heterocyclic compounds.^[12]^ Several TYRIs contain crucial moiety able to establish a network of interaction within catalytic site in place of corrected substrate L‐tyrosine or L‐DOPA in monophenolase or diphenolase reaction; so, these compounds display an obvious competitive inhibition of TYR activity.^[4]^ The phenolic fragment constitutes the best representative example of crucial chemical feature for tyrosinase inhibition (see Figure 1).^[8]^ In addition, the 4‐fluorophenyl moiety appears a recurrent chemical feature of different scaffolds with potent inhibitory efficacy against tyrosinase.[^13^, ^14^, ^15^, ^16^, ^17^, ^18^, ^19^, ^20^, ^21^] Overall, the above‐reported structures of TYRIs are characterized by the presence of two main portions: 1) phenolic moiety as the primary pharmacophoric element for the optimal binding recognition within catalytic site mimicking L‐tyrosine; 2) an aromatic tail as secondary pharmacophoric moiety, which establishes profitable contacts with hydrophobic area at the entrance of catalytic cavity. These two crucial fragments are linked by a molecular fragment able to create additional interactions.

**Figure 2:**
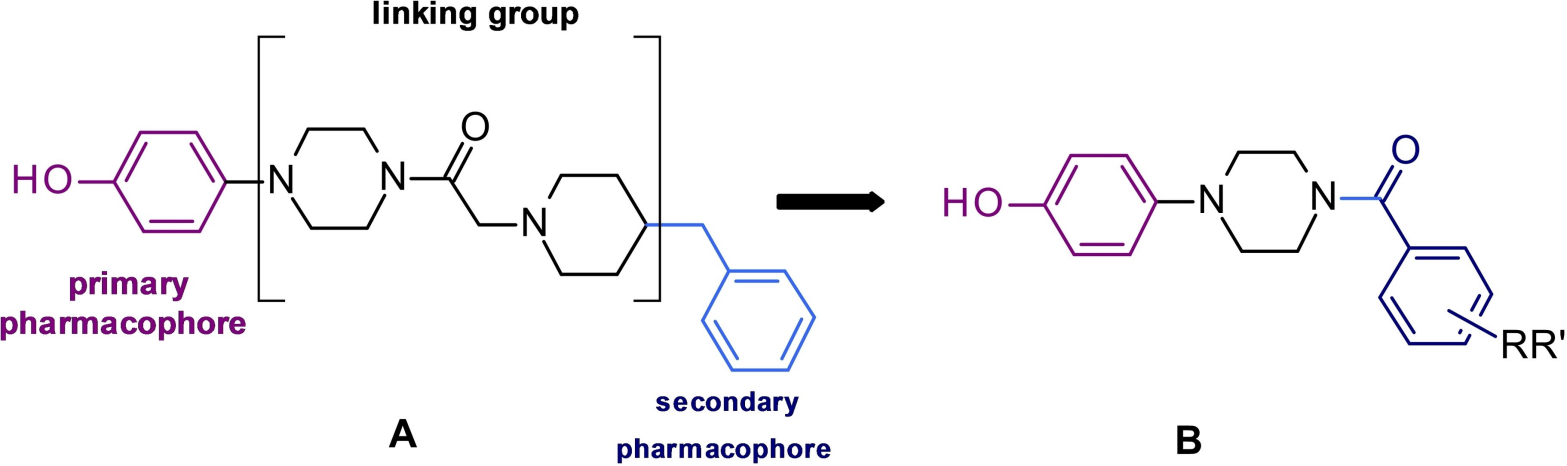
(4‐Hydroxyphenyl)piperazine‐based tyrosinase inhibitor^[15]^ and designed compounds.

In our previous paper, we have demonstrated that the 2‐(4‐benzyl‐1‐piperidinyl)‐1‐[4‐(4‐hydroxyphenyl)piperazin‐1‐yl]ethenone (**A**) proved to inhibit AbTYR (IC_50_=3.8 μM) and exert antioxidant effects.^[15]^ As a continuation of these studies, we now report the design and synthesis of new 4‐hydroxyphenylpiperazine‐based compounds (**B**) as the general structure that is depicted in Figure 2. In detail, we chose to reduce the length and flexibility of the spacer that connects the aromatic tail to the piperazine central core. Moreover, the secondary pharmacophoric feature was modified incorporating a wide series of substituents on aromatic tail. The structure‐affinity relationships, *in silico* studies and biological characterization are reported.

## Results and Discussion

### Active fragment confirmation and design of new inhibitors

**Figure 3:**
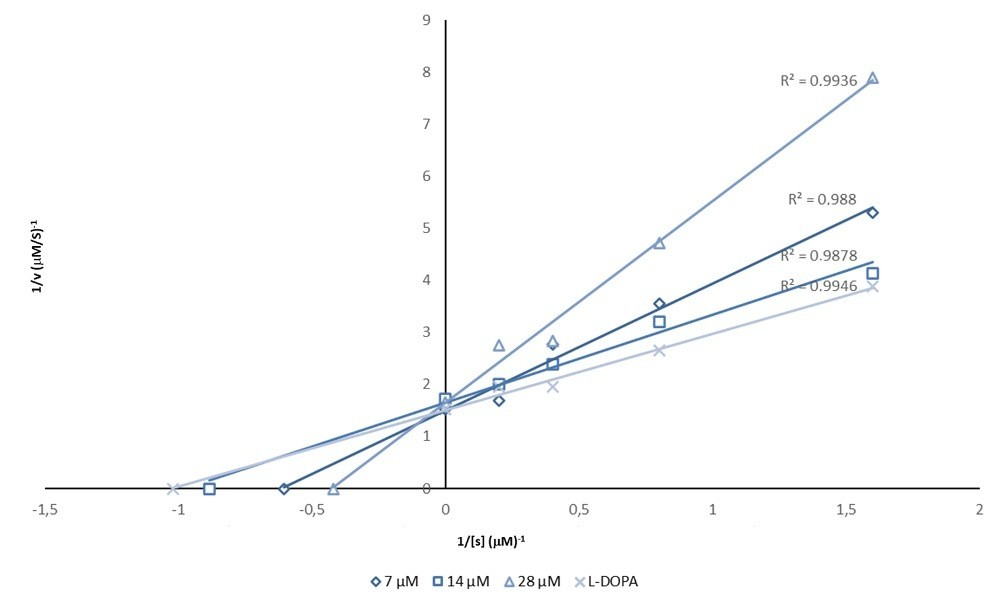
Lineweaver‐Burk plot for inhibition of AbTYR in presence of the compound **1** tested at concentrations of 7, 14 and 28 μM.

Initially, the starting material 4‐(1‐piperazinyl)phenol (**1**) has been studied in biochemical assays towards AbTYR; we detected a promising IC_50_ value of 28.9 μM (see Table 1) and kinetic studies disclosed that compound **1** is able to interfere with AbTYR catalytic cycle by acting as a competitive inhibitor (Figure 3).

**Table 1:** Biochemical data of **1**–**35** and reference compound Kojic acid (**KA**) for their inhibition of diphenolase activity of AbTYR.

Entry	R	IC_50_ [μM]^[a]^±SD^[b]^
**1**	–	28.9±4.9
**2**	H	73.2±3.5
**3**	4‐C_6_H_5_	128.3±4.1
**4**	2‐F	15.2±0.9
**5**	3‐F	22.6±1.3
**6**	4‐F	21.7±1.2
**25**	2,4‐F_2_	17.5±1.3
**7**	2‐Cl	2.6±0.3
**8**	3‐Cl	9.0±1.0
**9**	4‐Cl	8.9±0.9
**10**	2,4‐Cl_2_	1.5±0.1
**11**	2‐Br	4.5±0.6
**12**	3‐Br	16.4±1.9
**13**	4‐Br	39.6±0.9
**26**	2,4‐Br_2_	9.5±0.3
**14**	2‐CH_3_	29.9±2.0
**15**	3‐CH_3_	31.7±0.5
**16**	4‐CH_3_	34.4±0.5
**27**	2,4‐(CH_3_)_2_	33.7±0.7
**17**	2‐CF_3_	4.6±0.3
**18**	3‐CF_3_	5.5±0.2
**19**	4‐CF_3_	35.1±0.5
**20**	2,4‐(CF_3_)_2_	18.2±2.4
**21**	2‐OCH_3_	3.5±0.2
**22**	3‐OCH_3_	9.8±0.4
**23**	4‐OCH_3_	14.9±0.7
**24**	2,4‐(OCH_3_)_2_	16.7±2.0
**28**	2‐NO_2_	7.4±0.7
**29**	3‐NO_2_	21.8±1.2
**30**	4‐NO_2_	23.2±1.5
**31**	2,4‐(NO_2_)_2_	7.6±0.05
**32**	2‐NH_2_	66.4±2.1
**33**	3‐NH_2_	35.7±1.05
**34**	4‐NH_2_	43.9±3.4
**35**	2,4(NH_2_)_2_	82.4±1.1
**KA**	–	17.8±0.2

[a] All compounds were examined in a set of experiments performed in three replicates; IC50 values represent the concentration that caused 50 % enzyme activity loss. [b] SD indicates standard deviation.

**Scheme 1:**
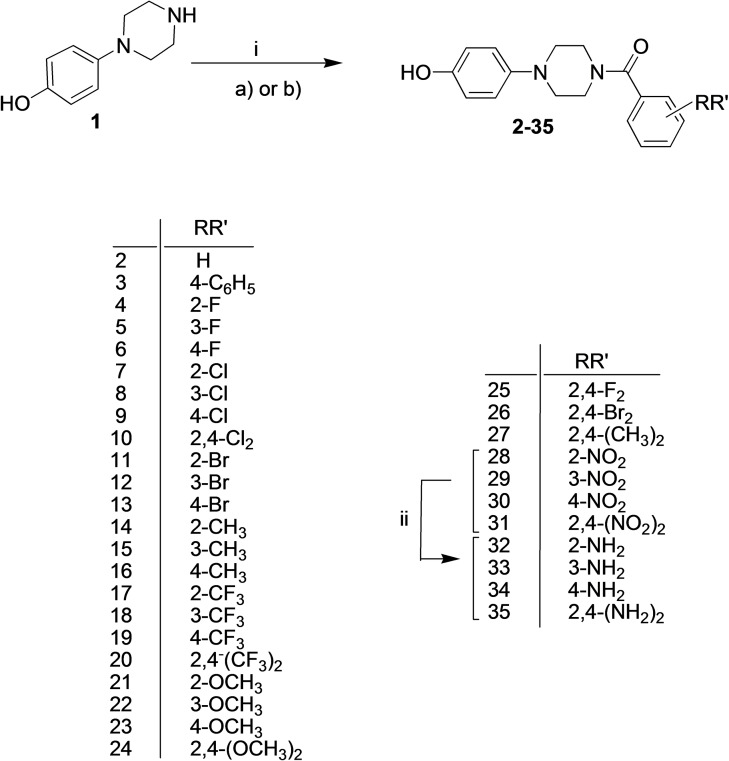
i: a) suitable benzoyl chloride for compounds **2**–**24**, DIPEA, DMF, 5 h, rt; or b) appropriate benzoic acid for compounds **25**–**31**, HBTU, TEA, DMF, overnight, rt; ii) Zn, HCl, EtOH, 2 h, reflux.

This evidence appointed **1** as plausible substrate‐like inhibitor and inspired us in the development of a series of new compounds bearing the (4‐hydroxyphenyl)piperazine group as structural requirement for recognition in to the TYR catalytic cavity. A library of type **B** (*cfr* Figure 2) thirty‐four new compounds was synthesized by following the route drawn in Scheme 1. By coupling the suitable benzoyl chlorides or benzoic acids with the 4‐(1‐piperazinyl)phenol in basic conditions at room temperature, we obtained the 4‐(4‐hydroxyphenyl)piperazine‐based compounds **2**–**31** in good yields. Then, the four amino‐derivatives **32**–**35** were synthesized by zinc‐mediated nitro‐reduction of parent four nitro‐compounds **28**–**31**.

### Assessment of AbTYR inhibitory effects

All synthesized compounds were screened toward the diphenolase activity of AbTYR as a surrogate test of hTYR inhibition in our preliminary screening of new potential agents; the results are reported below in comparison with reference compound kojic acid (KA) as well as active precursor compound **1** (Table 1).

As shown in Table 1, the 4‐hydroxyphenylpiperazine derivatives **2**–**35** are able to inhibit AbTYR and most of them at low micromolar value. By analyzing the data collected in Table 1, we achieved the following structure‐activity relationship considerations about the influence on AbTYR inhibition made by the introduction of aroyl moiety on the nitrogen atom of piperazine ring of precursor compound **1**. Firstly, the unsubstituted compound **2** (R=H, IC_50_=73.2 μM) and the corresponding 4‐phenyl‐substituted analog **3** (IC_50_=128.3 μM) displayed poor activity when compared to compound **1** (IC_50_=28.9 μM).

The absence of substituents or the presence of bulky substituents on the aromatic moiety negatively influence the AbTYR inhibition.

Compounds **7**, **11**, **17**, and **21** bearing several EDG or EWG substituents (R=Cl, Br, CF_3_, OCH_3_) at C‐2’ position of benzoyl ring resulted the best potent inhibitors displaying IC_50_ values <5 μM so that they were about 3.5‐fold more potent than reference compound KA (IC_50_=17.8 μM). Notably, the introduction of additional chlorine atom at C‐4’ position improved the potency, so that the 4‐(4‐hydroxyphenyl)piperazin‐1‐yl)(2,4‐dichlorophenyl)methanone (**10**) showed inhibitory effect at very low micromolar concentration (IC_50_ value of 1.5±0.1 μM). Other aromatic substituents at C‐2’ (R=F and NO_2_) or C‐3’/C‐4’ positions (R=F, Cl, Br, CF_3_, NO_2_, OCH_3_) were generally well tolerated and compounds **4**–**6**, **8**–**9**, **12**, **18**, **20**, **22**–**26**, **28**–**31** were more potent than precursor compound **1**. In contrast, compounds **13**–**16**, **19**, **32**–**34** demonstrated lower affinity respect to precursor compound **1**; however, these compounds inhibited AbTYR with higher potency when compared to unsubstituted compound **2**, thus suggesting that the decoration of aryl moiety generally improved the ability to establish profitable interactions within catalytic site of enzyme. Finally, the presence of a pair of hydrophilic amino‐substituents led to the poor active inhibitor **35**.

The most active compounds **7**, **10**, **11**, **17**, **21** were chosen for further biochemical assays using L‐Tyr as substrate to test inhibitory effects for monophenolase activity of AbTYR; further profiling of these (4‐hydroxyphenyl)piperazine‐based compounds confirmed that they were able to act as active inhibitors (data displayed in Supporting Information).

### Kinetic studies

**Figure 4:**
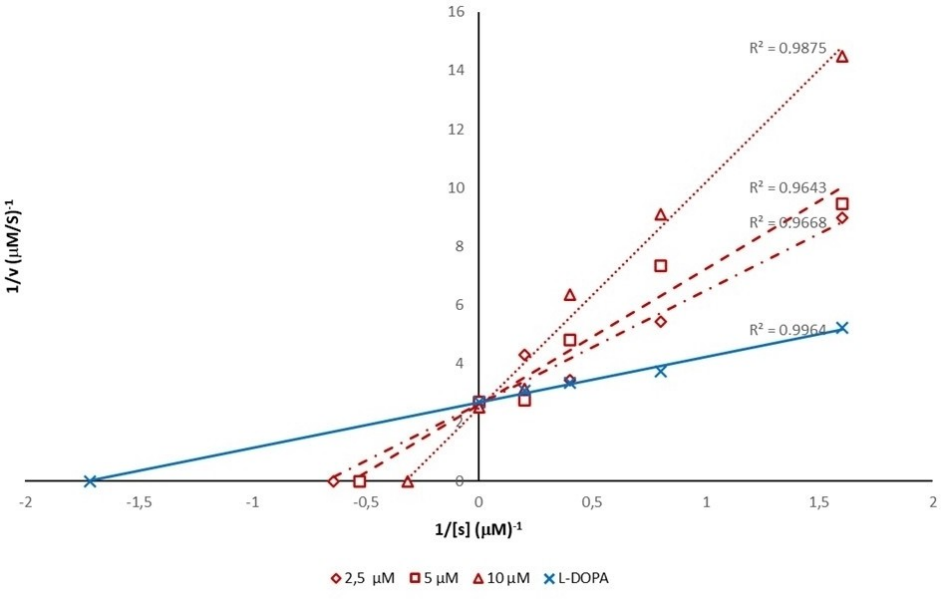
Lineweaver‐Burk plot for inhibition of AbTYR in presence of the compound **10** tested at concentrations of 2.5, 5 and 10 μM.

**Figure 5:**
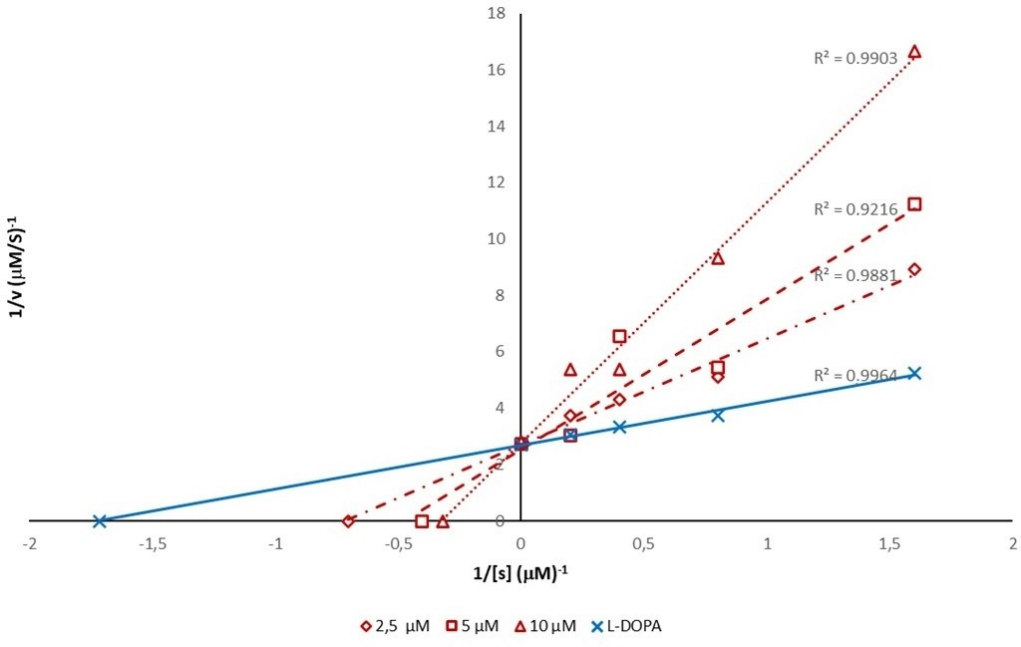
Lineweaver‐Burk plot for inhibition of AbTYR in presence of the compound **21** tested at concentrations of 2.5, 5 and 10 μM.

Considering the effects against both diphenolase and monophenolase activity of AbTYR, we decided to explore the mechanism of inhibition for this new series of TYRIs from synthetic source thus selecting compounds **10** and **21**, that are characterized by EWG or EDG substituents (R=Cl or OCH_3_) on aromatic tail. Kinetics studies were performed in presence of three different concentrations of selected inhibitors, L‐DOPA (0.6–5 mM) and AbTYR in phosphate buffer (0.05 M, pH 6.8). Results are reported using Lineweaver‐Burk double reciprocal plots (see Figures 4 and 5) and gave straight lines intersecting on Y‐axis. This behavior revealed that tested compounds **10** and **21** were competitive inhibitors. These data were consistent with mechanism of inhibition determined for their parent compound **1** (*cfr* Figure 3).

### Docking analysis

In order to gain information about the binding mode of the studied derivatives, we performed consensus docking studies on AbTYR by using three different docking programs: Gold V2020.2.0,^[22]^ Glide V8.8[^23^, ^24^, ^25^] and AutoDock V4.2.6.[^26^, ^27^] This method was based on the combination of the results from different programs to improve the prediction of the correct pose compared to each individual procedure.^[28]^ To perform this study we retrieved the crystal structure of AbTYR in complex with the inhibitor tropolone in the RCSB Protein Data Bank (PDB ID: 2Y9X).^[29]^

**Figure 6:**
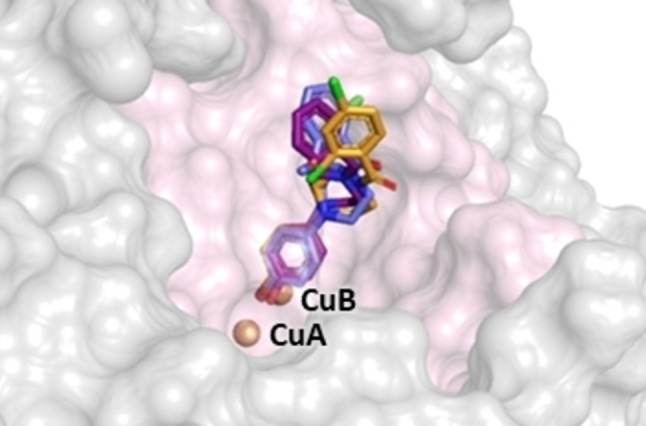
Superimposition of the docked poses in the catalytic cavity of AbTYR. The 4‐hydroxyphenyl moiety of the ligand **7** (deep purple sticks), **10** (bright orange sticks) and **21** (slate sticks) is located towards the copper ions CuA and CuB (brown spheres) in the binding site (light pink surface) of AbTYR, represented as surface (PDB code: 2Y9X). The figure was created by means of PyMOL software.^[30]^

In Figure 6 is showed the results of selected active derivatives **7** (R=Cl), **10** (R=2,4‐Cl_2_) and **21** (2‐OCH_3_) as prototypes of this class of inhibitors bearing (4‐hydroxyphenyl)piperazine moiety anchored to a substituted aroyl fragment.

The binding mode of the inhibitors in the AbTYR pocket was analysed through Maestro V12.5.139.^[31]^ As shown in Figure 6 all ligands exhibited the same orientation in which the 4‐hydroxyphenyl moiety was placed between the two copper ions. The corresponding 2 D interaction diagrams are reported in Supporting Information.

**Figure 7:**
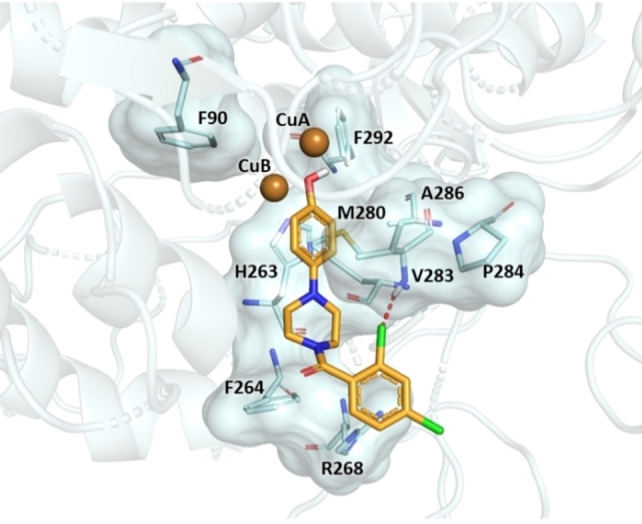
Molecular interactions of the docked poses of compound **10** (bright orange) in the binding site of AbTYR. The residues interacting with compound are displayed as both surface and stick model, the copper ion as brown sphere. The halogen and hydrogen bond are respectively depicted in red and yellow. The figure was created by means of PyMOL software.^[30]^

In Figure 7 it is possible to see the binding mode of the most active ligand **10**, that established metal coordination with both copper ions (CuA and CuB) whereas the phenyl ring engaged π‐π interaction with imidazole ring of H263 residue, suggesting that these contacts play a crucial role for the positioning within the binding pocket. Furthermore, this portion exhibited hydrophobic interactions with F90, F292, M280, V283 and A286. The piperazine ring was involved in hydrophobic contacts with the residues V283 and F264. Moreover, the docking pose of compound **10** predicted (i) a π‐cation interaction between aroyl moiety and R268 side chain, (ii) a halogen bond between o‐chlorine substituent and NH backbone of V283, and (iii) a hydrophobic interaction with F264. These interactions might contribute to improving the stability of the potent ligand **10** in the catalytic cavity.

To have an idea if these molecules could target human isoform, we further investigated the capability of these ligands to bind the catalytic cavity of hTYR. To achieve this purpose, we employed the homology model built using TRP‐1 as template due to the high similarity with hTYR. Actually, TRP‐1 and hTYR share 40 % of residues sequence identity and four conserved regions, including a tyrosinase‐like subdomain in the intra melanosomal region, in which is placed the active site.^[1]^ For our consensus docking studies we applied the same protocol described for AbTYR (*cfr supra*).

**Figure 8:**
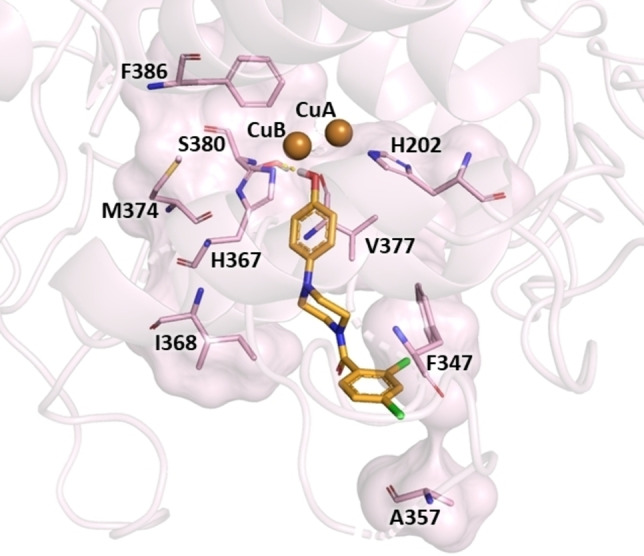
Molecular interactions of the docked pose of compound **10** (bright orange) in the binding site of hTYR. The residues interacting with compound are displayed as both surface and stick model, the copper ions as brown spheres. The halogen and hydrogen bond are respectively depicted in red and yellow. The figure was created by means of PyMOL software.

Docking simulations revealed that compound **10** was able to occupy the catalytic site of hTYR (Figure 8); 4‐hydroxyphenyl moiety was oriented towards the copper ions, in which a hydrogen bond was observed between hydroxyl function and side chain of S380, while the phenyl ring engaged π‐π interaction with both H367 and H202 imidazole rings, and hydrophobic contact with M374, V377 and F386. For piperazine core, the docking pose predicted hydrophobic interaction with I368. Finally, the aroyl moiety was involved in π‐π interaction with F347 and hydrophobic contact with A357.

**Figure 9:**
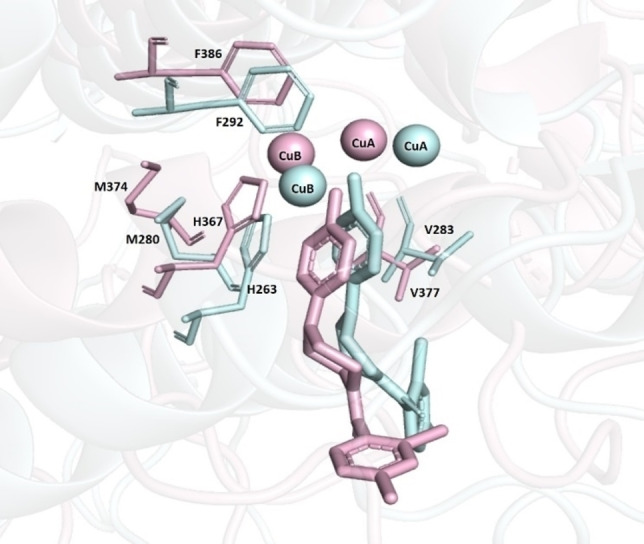
Superimposed docking models of compound 10 bound to the catalytic cavities of AbTYR (ligand and protein coloured in cyan stick) and hTYR (ligand and protein coloured in pink stick). The figure was created by means of PyMOL software

Analysing the docking data on AbTYR and hTYR, it is possible to provide a comparison of the contacts established between the compound **10** and the amino acids of these two distinct tyrosinases. Compound **10** showed a similar orientation in the catalytic site of the two proteins coordinating the copper ions. Furthermore, in the AbTYR established π‐π interaction with H263 and hydrophobic interactions with M280, V283 and F292, which corresponded to the residues H367, V377, M374 and F386 of hTYR. Therefore, we can speculate that compound **10** might display affinity toward hTYR, thus assuming a similar mode of interaction for the critical 4‐hydroxyphenyl moiety as displayed in Figure 9.

### Cell viability and cellular tyrosinase activity

Cell viability assay (MTT) based on mitochondria efficiency was used for selected active inhibitors **7**, **10**, **11**, **17**, and **21**, bearing distinct substituents on the accessory pharmacophoric benzoyl‐moiety. All compounds exhibited no cytotoxic effect until a concentration of 10 μM (see Figure S71 in Supporting Information). Compound **10**, the best compound of the series, showed a good cell viability until 25 μM. Thus, further experiments at 25 μM of compound were performed.

### Effects of compound 10 on tyrosinase activity in B16F10 cells with α‐MSH stimulation

To gain evidence of compound **10** involvement in melanogenesis, its effects on melanin production in B16F10 cells were evaluated by tyrosinase zymography. ImageJ software was used to determine the intensity of bands.

**Figure 10:**
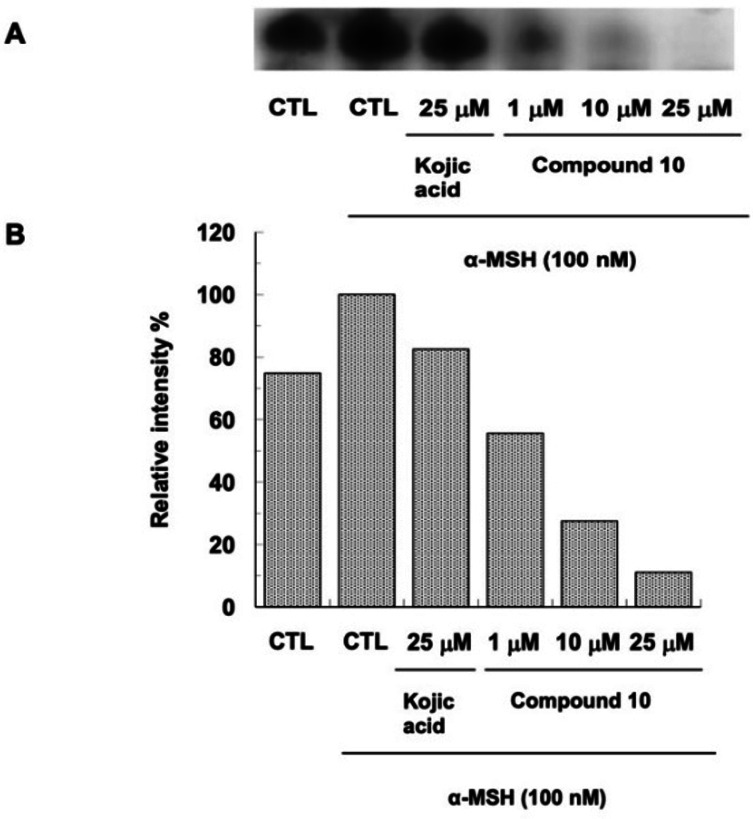
Effect of compound **10** on tyrosinase activity by L‐DOPA staining (**A**). Relative intensity of bands was determined by ImageJ software (**B**).

Figure 10 shows the effect of compound **10** and kojic acid on tyrosinase activity in α‐MSH‐stimulated B16F10 cells.

After treatment only with hormone α‐MSH, the tyrosinase activity appears significantly increased if compared with untreated cells. Compound **10** significantly reduced melanogenesis of α‐MSH‐stimulated B16F10 cells in a dose‐dependent manner, with 44 % of inhibition at concentration of 1 μM, 73 % at 10 μM and 88 % at 25 μM. Compound **10** exhibited excellent inhibitory properties already at the concentration of its IC_50_ value. Instead, kojic acid at concentration of 25 μM showed an inhibition of 17 %.

A cellular model was used to confirm the AbTYR inhibitory activities of compound **10**.

### Antioxidant activity

The antioxidant property of compounds **7**, **10**, **11**, **17** and **21** was also evaluated by ABTS^.+^ assay and the results are represented as EC_50_ values in Table 2. All the compounds were found to possess an ability to quench ABTS radical and displayed a scavenging activity comparable or even better to that of the positive control. Interestingly, compound **10** that was also the most active inhibitor, showed a good antioxidant activity with EC_50_ value of 9.0 μM.

**Table 2:** Antioxidant activity of compounds **7**, **10**, **11**, **17** and **21**.

Compound	EC_50_ [μM]^[a]^
**7**	13.2±0.5
**10**	9.0±0.3
**11**	13.2±1.1
**17**	9.0±0.9
**21**	9.2±0.2
**Trolox** ^[b]^	13.0±1.1^[32]^

[a] Data represent the mean (±standard deviation, SD) of three independent experiments. [b] Positive control.

## Conclusion

In conclusion, our studies might contribute to extend the knowledge on SAR for several phenolic competitive TYRIs from synthetic source. The positioning of one or a pair of hydrophobic substituents on benzoyl pharmacophoric portion is critical for optimizing the orientation of aromatic tail and improving inhibitory potency toward TYRs. Further, it was apparent that the introduction of hydrophilic functional groups negatively influenced the affinity as well as additional phenyl ring. The piperazine core seems to contribute to address the correct orientation of the two pharmacophoric structural requirements that are hydroxy‐phenyl fragment linked to aromatic tail. In consideration for future design of new hydroxyphenyl‐based compounds, we highlighted the preference for halogen and hydrogen bond contacts through substituents located at the ortho‐position. Five potent competitive TYR inhibitors were disclosed and their biological profile was characterized. Docking studies suggested the main interactions within TYR catalytic site. Overall, a combination of biological data and *in silico* simulations allowed as to identify new potential anti‐melanogenic agents able to exert antioxidant properties in absence of cytotoxicity. Based on the knowledge that hTYR and AbTYR could display different substrate specificities in the case of distinct inhibitors, we believe that additional and appropriate tests against human systems are needed to further ascertain the anti‐hTYR application.

## Experimental Section

### Chemistry

Chemicals were used without further purification and bought from common commercial suppliers (Sigma‐Aldrich, Milan, Italy and Alfa Aesar, Karlsruhe, Germany). Melting points were determined on a Büchi B‐545 apparatus (Büchi Labortechnik AG Flawil, Switzerland) and are uncorrected. By combustion analysis (C, H, N) carried out on a Carlo Erba Model 1106‐Elemental Analyzer, we determined the purity of synthesized compounds; the results confirmed a ≥95 % purity. Merck Silica Gel 60 F254 plates were used for analytical thin‐layer chromatography (TLC; Merck KGaA, Darmstadt, Germany). For detection UV light (254 nm) was used. ^1^H NMR spectra and ^13^C NMR spectra were measured in dimethylsulfoxide‐d6 (DMSO‐d_6_) with a Varian Gemini 500 spectrometer (Varian Inc. Palo Alto, California USA); chemical shifts are expressed in δ (ppm) and coupling constants (*J*) in hertz (Hz). All exchangeable protons were confirmed by addition of D_2_O. R_*f*_ values were determined on TLC plates using a mixture of CH_2_Cl_2_/CH_3_OH (96 : 4) as eluent.

### General procedures for the synthesis of compounds 2–35

Pathway i) Synthesis of (4‐(4‐hydroxyphenyl)piperazin‐1‐yl)methanone derivatives **2**–**24**. To a mixture of 4‐Piperazin‐1‐yl phenol (200 mg, 1.12 mmol) and *N*,*N*‐diisopropylethylamine (DIPEA, 292.6 μL, 1.68 mmol) in Dimethylformamide (DMF, 4 mL) at 0 °C, the suitable benzoyl chloride derivative (1.12 mmol) was added dropwise. The reaction was stirred overnight, at room temperature. After completion, water was added and the mixture was extracted with EtOAc (3×10 mL). The obtained organic phase was washed many times with brine (3×15 mL) and dried with anhydrous Na_2_SO_4_. The solvent was removed under reduced pressure and the final products were crystalized with diethyl ether and ethanol, or cyclohexane and dichloromethane.

Pathway ii) Synthesis of (4‐(4‐hydroxyphenyl)piperazin‐1‐yl)methanone derivatives **25**–**31**. A mixture of the suitable benzoic acid derivative (1.12 mmol) and *N*,*N*,*N’*,*N’*‐tetramethyl‐*O*‐(1*H*‐benzotriazol‐1‐yl)uronium hexafluorophosphate (HBTU, 424.8 mg, 1.12 mmol) in DMF (4 mL) was stirred at room temperature for 1 hour. After that, a solution of the 4‐Piperazin‐1‐yl phenol (200 mg, 1.12 mmol) and Triethylamine (TEA, 156 μL, 1.12 mmol) in DMF was added. The reaction was carried out overnight, at room temperature. Then, it is quenched with water (10 mL) and extracted with EtOAc (3×10 mL). The organic phase was washed with brine (3×15 mL) and dried with anhydrous Na_2_SO_4_. The solvent was removed in vacuo and the residues were purified by crystallization with Et_2_O, leading to the final compounds.

Excluding compounds **20**, **26**, **31** and **35**, for all synthetized compounds registered CAS numbers have been already assigned. However, their synthetic procedures, chemical properties and structural characterization are not available in literature, except for derivatives **2**, **6**, **30**, and **32**.[^33^, ^34^, ^35^]


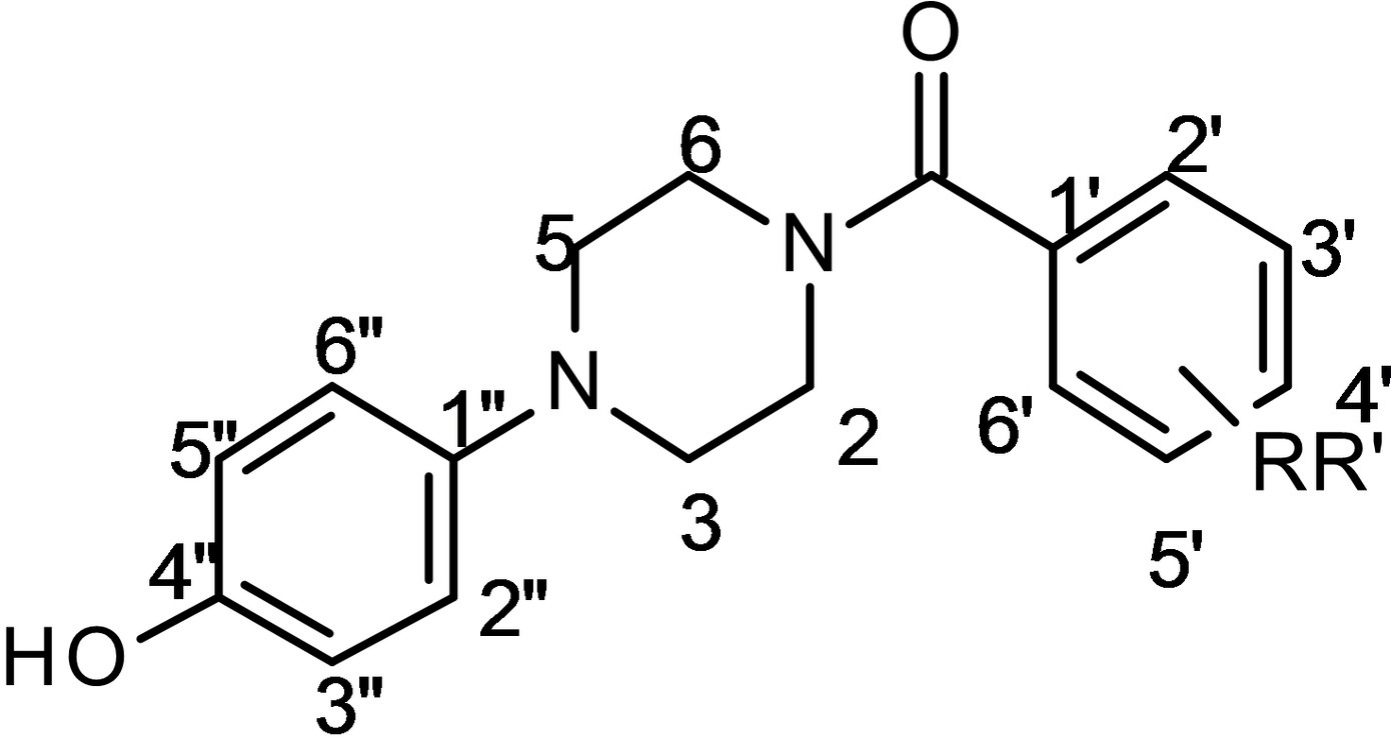


### (4‐(4‐Hydroxyphenyl)piperazin‐1‐yl)(phenyl)methanone (2)

CAS Number: 684249‐53‐4. Yield: 62 %. White powder. M.p. 170–171 °C. R_*f*_: 0.34. ^1^H‐NMR (500 MHz, DMSO‐*d*_6_): (δ) 2.96 (m, 4H, 2CH_2_), 3.44–3.75 (m, 4H, 2CH_2_), 6.69 (d, *J=8.6 Hz*, 2H, ArH, H‐2“ and H‐6“), 6.81 (d, *J*=8.6 Hz, 2H, ArH, H‐3“ and H‐5“), 7.42–7.46 (m, 5H, ArH, H‐2‘,3‘,4‘,5‘ and 6‘), 8.91 (s, 1H, OH). ^13^C‐NMR (126 MHz, DMSO‐d_6_): (δ) 41.7 (CH_2_), 47.2 (CH_2_), 50.44 (C‐3 and C‐5), 115.5 (C‐2“ and C‐6“), 118.5 (C‐3“ and C‐5“), 126.7 (C‐2‘ and C‐6‘), 128.4 (C‐3“ and C‐5“), 129.5 (C‐4‘), 135.9 (C‐1‘), 143.9 (C‐1“), 151.4 (C‐4“), 168.9 (C=O). Anal. Calcd for (C_17_H_18_N_2_O_2_): C 72.32, H 6.43, N 9.92. Found: C 72.29, H 6.40, N 9.87.

### (4‐(4‐Hydroxyphenyl)piperazin‐1‐yl)(biphenyl‐4‐yl)methanone (3)

CAS Number: 1625802‐45‐0. Yield: 65 %. Light grey powder. M.p. 204–205 °C. R_*f*_: 0.37. ^1^H‐NMR (500 MHz, DMSO‐*d*_6_): (δ) 2.98 (bs, 4H, 2CH_2_), 3.52–3.76 (m, 2H, 2CH_2_), 6.70 (d, *J=8.6 Hz*, 2H, ArH, H‐2“ and H‐6“), 6.82 (d, J=8.6 Hz, 2H, ArH, H‐3“ and H‐5“), 7.39 (t, *J=7.3 Hz*, 1H, ArH, H‐4“‘), 7.48 (t, *J=7.5 Hz*, 2H, ArH, H‐3“‘ and H‐5“‘), 7.52 (d, *J=7.9 Hz*, 2H, ArH, H‐2“‘ and H‐6“‘) 7.70 (d, *J=7.7 Hz*, 2H, ArH, H‐3‘ and H‐5‘), 7.74 (d, *J=7.8 Hz*, 2H, ArH, H‐2‘ and H‐6‘), 8.93 (s, 1H, OH). ^13^C‐NMR (126 MHz, DMSO‐d_6_): (δ) 41.7 (CH_2_), 47.3 (CH_2_), 50.5(C‐3 and C‐5), 115.5 (C‐2“ and C‐6“), 118.5 (C‐3“ and C‐5“), 126.6 (C‐2‘ and C‐6‘), 126.8 (C‐2“‘ and C‐6“‘), 127.7 (C‐3‘ and C‐5), 127.8 (C‐3“‘ and C‐5“‘), 129.0 (C‐4“‘), 134.7 (C‐1‘), 139.3 (C‐4‘), 141.3 (C‐1“‘), 143.9 (C‐1“), 151.5 (C‐4“), 168.7 (C=O). Anal. Calcd for (C_23_H_22_N_2_O_2_): C 77.07, H 6.19, N 7.82. Found: C 77.01, H 6.22, N 7.88.

### (4‐(4‐Hydroxyphenyl)piperazin‐1‐yl)(2‐fluorophenyl)methanone (4)

CAS Number: 1623536‐33‐3. Yield: 60 %. White powder. M.p. 142–143 °C. R_*f*_: 0.38. ^1^H‐NMR (500 MHz, DMSO‐*d*_6_): (δ) 2.89 (m, 2H, CH_2_), 3.01 (m, 2H, CH_2_), 3.32 (m, 2H, CH_2_), 3.77 (m, 2H, CH_2_), 6.66 (d, *J=8.9 Hz*, 2H, ArH, H‐2“ and H‐6“), 6.81 (d, *J=8.9 Hz*, 2H, ArH, H‐3“ and H‐5“), 7.28–7.32 (m, 2H, ArH, H‐3‘ and H‐5‘), 7.41–7.44 (m, 1H, ArH, H‐4‘), 7.49–7.53 (m, 1H, ArH, H‐6‘), 8.92 (s, 1H, OH). ^13^C‐NMR (126 MHz, DMSO‐d_6_): (δ) 41.5 (CH_2_), 46.6 (CH_2_), 50.4 (CH_2_), 50.6 (CH_2_), 115.5 (C‐2“ and C‐6“), 115.8 (d, *J* ^*2*^_C‐F_=20.9 Hz, C‐3‘), 118.6 (C‐3“ and C‐5“), 124.0 (d, *J* ^*2*^_C‐F_=17.1 Hz, C‐1‘), 124.9 (C‐5‘), 128.9 (C‐6‘), 131.5 (d, *J* ^*3*^_C‐F_=7.3 Hz, C‐4‘), 143.8 (C‐1“), 151.5 (C‐4“), 157.5 (d, *J* ^*1*^_C‐F_=245.6 Hz, C‐2‘), 163.9 (C=O). Anal. Calcd for (C_17_H_17_FN_2_O_2_): C 67.99, H 5.71, N 9.33. Found: C 68.03, H 5.74, N 9.28.

### (4‐(4‐Hydroxyphenyl)piperazin‐1‐yl)(3‐fluorophenyl)methanone (5)

CAS Number: 1625819‐84‐2. Yield: 70 %. White powder. M.p. 145–146 °C. R_*f*_: 0.39. ^1^H‐NMR (500 MHz, DMSO‐*d*_6_): (δ) 2.96 (m, 6H, 3CH_2_), 3.74 (bs, 2H, CH_2_), 6.65 (d, *J=8.8 Hz*, 2H, ArH, H‐2“ and H‐6“), 6.81 (d, *J=8.9 Hz*, 2H, ArH, H‐3“ and H‐5“), 7.25–7.32 (m, 3H, ArH, H‐2‘, 4‘ and 6‘), 7.48–7.53 (m, 1H, ArH, H‐5‘), 8.91 (s, 1H, OH). ^13^C‐NMR (126 MHz, DMSO‐d_6_): (δ) 41.7 (CH_2_), 47.1 (CH_2_), 50.3 (C‐3 and C‐5), 114.0 (d, *J* ^2^_C‐F_=22.6 Hz, C‐2‘), 115.5 (C‐2“ and C‐6“), 116.4 (d, *J* ^2^_C‐F_=20.9 Hz, C‐4‘), 118.6 (C‐3“ and C‐5“), 123.0 (d, *J* ^4^_C‐F_=2.9 Hz, C‐6‘), 130.7 (d, *J* ^3^_C‐F_=8.1 Hz, C‐5‘), 138.2 (d, *J* ^3^_C‐F_=7.0 Hz, C‐1‘), 143.8 (C‐1“), 151.5 (C‐4“), 161.9 (d, *J* ^1^_C‐F_=245.7 Hz), 167.5 (C=O). Anal. Calcd for (C_17_H_17_FN_2_O_2_): C 67.99, H 5.71, N 9.33. Found: C 67.96, H 5.70, N 9.36.

### (4‐(4‐Hydroxyphenyl)piperazin‐1‐yl)(4‐fluorophenyl)methanone (6)

CAS Number: 684249‐42‐1. Yield: 69 %. White powder. M.p. 166–167 °C. R_*f*_: 0.36. ^1^H‐NMR (500 MHz, DMSO‐*d*_6_): (δ) 2.96 (bs, 6H, 3CH_2_), 3.72 (bs, 2H, CH_2_), 6.66 (d, *J=8.8 Hz*, 2H, ArH, H‐2“ and H‐6“), 6.81 (d, *J=8.9 Hz*, 2H, ArH, H‐3“ and H‐5“), 7.26–7.30 (m, 2H, ArH, H‐3‘ and H‐5‘), 7.47–7.51 (m, 2H, ArH, H‐2‘ and H‐6‘), 8.91 (s, 1H, OH). ^13^C‐NMR (126 MHz, DMSO‐d_6_): (δ) 41.8 (CH_2_), 47.3 (CH_2_), 50.4 (C‐3 and C‐5), 115.4 (d, *J* ^*2*^_C‐F_=21.6 Hz, C‐3‘ and C‐5‘), 115.5 (C‐2“ and C‐6“), 118.5 (C‐3“ and C‐5“), 129.6 (d, *J* ^*3*^_C‐F_=8.6 Hz, C‐2‘ and C‐6‘), 132.2 (d, *J* ^*4*^_C‐F_=3.2 Hz, C‐1‘), 143.8 (C‐1“), 151.4 (C‐4“), 162.5 (d, *J* ^*1*^_C‐F_=246.7 Hz, C‐4‘), 168.1 (C=O). Anal. Calcd for (C_17_H_17_FN_2_O_2_): C 67.99, H 5.71, N 9.33. Found: C 68.05, H 7.75, N 9.37.

### (4‐(4‐Hydroxyphenyl)piperazin‐1‐yl)(2,4‐difluorophenyl)methanone (25)

CAS Number: 1624035‐79‐5. Yield: 52 %. White powder. M.p. 180–181 °C. R_*f*_: 0.41. ^1^H‐NMR (500 MHz, DMSO‐*d*_6_): (δ) 2.90 (m, 2H, CH_2_), 3.01 (m, 2H, CH_2_), 3.34 (m, 2H, CH_2_), 3.77 (m, 2H, CH_2_), 6.67 (d, *J*=8.9 Hz, 2H, ArH, H‐2“ and H‐6“), 6.80 (d, *J*=8.9 Hz, 2H, ArH, H‐3“ and H‐5“), 7.19 (td, *J* ^*1*^=8.5 Hz, *J* ^*2*^=2.2 Hz, 1H, ArH, H‐5‘), 7.37 (td, *J* ^*1*^=9.7, *J* ^*2*^=2.4 Hz, 1H, ArH, H‐3‘), 7.51 (td, *J* ^*1*^=8.2, *J* ^*2*^=6.6 Hz 1H, ArH, H‐6’), 8.91 (s, 1H, OH). ^13^C‐NMR (126 MHz, DMSO‐d_6_): (δ) 41.6 (CH_2_), 46.7 (CH_2_), 50.3 (CH_2_), 50.7 (CH_2_), 104.4 (J^2^_C‐F_=26.4 Hz, C‐3‘), 112.3 (J^2^_C‐F_=21.3 Hz, C‐5‘), 115.5 (C‐2“ and C‐6“), 118.6 (C‐3“ and C‐5“), 120.7 (J^4^_C‐F_=3.8 Hz, C‐1‘), 130.5 (J^3^_C‐F_=9.1 Hz, C‐6‘),143.9 (C‐1“), 151.6 (C‐4“), 158.1 (J^1^_C‐F_=248.4 Hz, C‐2‘), 162.8 (J^1^_C‐F_=248.4 Hz, C‐4‘), 163.2 (C=O). Anal. Calcd for (C_17_H_16_F_2_N_2_O_2_): C 64.14, H 5.07, N 8.80. Found: C 64.20, H 5.02, N 8.76.

### (4‐(4‐Hydroxyphenyl)piperazin‐1‐yl)(2‐chlorophenyl)methanone (7)

CAS Number: 1625575‐85‐0. Yield: 55 %. White powder. M.p. 169–170 °C. R_*f*_: 0.41. ^1^H‐NMR (500 MHz, DMSO‐*d*_6_): (δ) 2.90 (m, 2H, CH_2_), 3.02 (m, 2H, CH_2_), 3.24 (m, 2H, CH_2_), 3.77 (m, 2H, CH_2_), 6.65 (d, *J=8.8 Hz*, 2H, ArH, H‐2“ and H‐6“), 6.80 (d, *J=8.9 Hz*, 2H, ArH, H‐3“ and H‐5“), 7.40 (m, 1H, ArH, H‐5‘), 7.45 (m, 2H, ArH, H‐3‘ and H‐4‘), 7.52–7.55 (m, 1H, ArH, H‐6‘), 8.91 (s, 1H, OH). ^13^C‐NMR (126 MHz, DMSO‐d_6_): (δ) 41.2 (CH_2_), 46.3 (CH_2_), 50.3 (CH_2_), 50.5 (CH_2_), 115.5 (C‐2“ and C‐6“), 118.6 (C‐3“ and C‐5“), 127.6 (C‐6‘), 128.0 (C‐5‘), 129.1 (C‐4‘), 129.4 (C‐3‘),130.4 (C‐2‘), 135.7 (C‐1‘), 143.7 (C‐1“), 151.5 (C‐4“), 165.4 (C=O). Anal. Calcd for (C_17_H_17_ClN_2_O_2_): C 64.46, H 5.41, N 8.84. Found: C 64.49, H 5.43, N 8.87.

### (4‐(4‐Hydroxyphenyl)piperazin‐1‐yl)(3‐chlorophenyl)methanone (8)

CAS Number: 1623965‐66‐1. Yield: 51 %. Light grey powder. M.p. 138–139 °C. R_*f*_: 0.44. ^1^H‐NMR (500 MHz, DMSO‐*d*_6_): (δ) 2.97 (m, 6H, 3CH_2_), 3.74 (bs, 2H, CH_2_), 6.65 (d, *J=8.8 Hz*, 2H, ArH, H‐2“ and H‐6“), 6.80 (d, *J=8.9 Hz*, 2H, ArH, H‐3“ and H‐5“), 7.37–7.40 (m, 1H, ArH, H‐5‘), 7.47–7.50 (m, 2H, ArH, H‐2‘ and H‐6‘), 7.52–755 (m, 1H, ArH, H‐4‘), 8.91 (s, 1H, OH).^13^C‐NMR (126 MHz, DMSO‐d_6_): (δ) 42.1 (CH_2_), 47.6 (CH_2_), 50.8 (C‐3 and C‐5), 115.9 (C‐2“ and C‐6“), 119.0 (C‐3“ and C‐5“), 126.0 (C‐6‘), 127.2 (C‐2‘), 129.9 (C‐4‘), 130.9 (C‐5‘), 133.7 (C‐3‘), 138.4 (C‐1‘), 144.2 (C‐1“), 151.9 (C‐4“), 167.8 (C=O). Anal. Calcd for (C_17_H_17_ClN_2_O_2_): C 64.46, H 5.41, N 8.84. Found: C 64.51, H 5.45, N 8.89.

### (4‐(4‐Hydroxyphenyl)piperazin‐1‐yl)(4‐chlorophenyl)methanone (9)

CAS Number: 1024179‐25‐6. Yield: 68 %. White powder. M.p. 172–173 °C. R_*f*_: 0.42. ^1^H‐NMR (500 MHz, DMSO‐*d*_6_): (δ) 2.96 (m, 6H, 3CH_2_), 3.73 (bs, 2H, CH_2_), 6.66 (d, *J*=8.9 Hz, 2H, ArH, H‐2“ and H‐6“), 6.80 (d, *J*=8.8 Hz, 2H, ArH, H‐3“ and H‐5“), 7.45 (d, *J*=8.4 Hz, 2H, ArH, C‐3‘ and C‐5‘), 7.52 (d, *J*=8.6 Hz, 2H, ArH, C‐2‘ and C‐6‘), 8.91 (s, 1H, OH). ^13^C‐NMR (126 MHz, DMSO‐d_6_): (δ) 41.8 (CH_2_), 47.3 (CH_2_), 50.4 (C‐3 and C‐5), 115.5 (C‐2“ and C‐6“), 118.5 (C‐3“ and C‐5“), 128.5 (C‐2‘ and C‐6‘), 129.0 (C‐3‘ and C‐5‘), 134.3 (C‐1‘), 134.6 (C‐4‘), 143.8 (C‐1“), 151.5 (C‐4“), 167.9 (C=O). Anal. Calcd for (C_17_H_17_ClN_2_O_2_): C 64.46, H 5.41, N 8.84. Found: C 64.42, H 5.38, N 8.82.

### (4‐(4‐Hydroxyphenyl)piperazin‐1‐yl)(2,4‐dichlorophenyl)methanone (10)

CAS Number: 1624139‐46‐3. Yield: 71 %. White powder. M.p. 177–178 °C. R_*f*_: 0.46. ^1^H‐NMR (500 MHz, DMSO‐*d*_6_): (δ) 2.90 (s, 2H, CH_2_), 3.01 (m, 2H, CH_2_), 3.24 (m, 2H, CH_2_), 3.75 (m, 2H, CH_2_), 6.65 (m, 2H, ArH, H‐2“ and H‐6“), 6.80 (m, 2H, ArH, H‐3“ and H‐5“), 7.44 (dd, J=8.2 Hz, 1H, ArH, H‐5‘), 7.52 (m, 1H, ArH, H‐3‘), 7.73 (t, J=1.8 Hz, 1H, ArH, H‐6‘), 8.93 (d, J=1.8 Hz, 1H, OH). ^13^C‐NMR (126 MHz, DMSO‐d_6_): (δ) 41.3 (CH_2_), 46.3 (CH_2_), 50.2 (CH_2_), 50.5 (CH_2_), 115.5 (C‐2“ and C‐6“), 118.6 (C‐3“ and C‐5“), 128.0 (C‐5‘ and C‐6‘), 129.2 (C‐3‘), 130.4 (C‐4‘), 134.3 (C‐1‘), 134.6 (C‐2‘), 143.7 (C‐1“), 151.5 (C‐4“), 164.6 (C=O). Anal. Calcd for (C_17_H_16_Cl_2_N_2_O_2_): C 58.14, H 4.59, N 7.98. Found: C 58.11, H 4.54, N 7.95.

### (4‐(4‐Hydroxyphenyl)piperazin‐1‐yl)(2‐bromophenyl)methanone (11)

CAS Number: 1623604‐87‐4. Yield: 68 %. White powder. M.p. 180–181 °C. R_*f*_: 0.39. ^1^H‐NMR (500 MHz, DMSO‐*d*_6_): (δ) 2.93 (m, 2H, CH_2_), 3.03 (m, 2H, CH_2_), 3.23 (t, *J*=5.2 Hz, 2H, CH_2_), 3.76 (m, 2H, CH_2_), 6.65 (d, *J*=8.7 Hz, 2H, ArH, H‐2“ and H‐6“), 6.80 (d, *J*=8.7 Hz, 2H, ArH, H‐3“ and H‐5“), 7.37 (m, 2H, ArH, H‐4‘ and H‐5‘), 7.47 (m, 1H, ArH, H‐3‘), 7.69 (m, 1H, ArH, H‐6‘), 8.91 (s, 1H, OH).^13^C‐NMR (126 MHz, DMSO‐d_6_): (δ) 41.1 (CH_2_), 46.4 (CH_2_), 50.2 (CH_2_), 50.4 (CH_2_), 115.5 (C‐2“ and C‐6“), 118.4 (C‐2‘),118.5 (C‐3“ and C‐5“), 128.0 (C‐5‘ and C‐6‘), 130.6 (C‐4‘), 132.5 (C‐3‘), 137.9 (C‐1‘), 143.7 (C‐1“), 151.5 (C‐4“), 166.3 (C=O). Anal. Calcd for (C_17_H_17_BrN_2_O_2_): C 56.52, H 4.74, N 7.75. Found: C 56.50, H 4.76, N 7.73.

### (4‐(4‐Hydroxyphenyl)piperazin‐1‐yl)(3‐bromophenyl)methanone (12)

CAS Number: 1624460‐64‐5. Yield: 83 %. White powder. M.p. 153–154 °C. R_*f*_: 0.41. ^1^H‐NMR (500 MHz, DMSO‐*d*_6_): (δ) 2.96 (m, 6H, 3CH_2_), 3.73 (bs, 2H, CH_2_), 6.66 (d, *J=8.9* Hz, 2H, ArH, H‐2“ and H‐6“), 6.81 (d, *J*=8.8 Hz, 2H, ArH, H‐3“ and H‐5“), 7.42 (m, 2H, ArH, H‐4‘ and H‐6‘), 7.61 (m, 1H, ArH, H‐5‘), 7.65–7.68 (m, 1H, ArH, H‐2‘), 8.91 (s, 1H, OH). ^13^C‐NMR (126 MHz, DMSO‐d_6_): (δ) 41.7 (CH_2_), 47.2 (CH_2_), 50.4 (C‐3 and C‐5), 115.5 (C‐2“ and C‐6“), 118.5 (C‐3“ and C‐5“), 121.7 (C‐3‘), 125.9 (C‐6‘), 129.6 (C‐5‘), 130.7 (C‐2‘), 132.3 (C‐4‘), 138.2 (C‐1‘), 143.8 (C‐1“), 151.4 (C‐4“), 167.2 (C=O). Anal. Calcd for (C_17_H_17_BrN_2_O_2_): C 56.52, H 4.74, N 7.75. Found: C 56.57, H 4.72, N 7.79.

### (4‐(4‐Hydroxyphenyl)piperazin‐1‐yl)(4‐bromophenyl)methanone (13)

CAS Number: 1024254‐02‐1. Yield: 41 %. White powder. M.p. 190–191 °C. R_*f*_: 0.39. ^1^H‐NMR (500 MHz, DMSO‐*d*_6_): (δ) 2.95 (m, 6H, 3CH_2_), 3.73 (bs, 2H, CH_2_), 6.67 (d, *J=8.4 Hz*, 2H, ArH, H‐2“ and H‐6“), 6.81 (d, *J*=8.4 Hz, 2H, ArH, H‐3“ and H‐5“), 7.39 (d, *J*=8.3 Hz, 2H, ArH, H‐3‘ and H‐5‘), 7.66 (d, *J*=8.3 Hz, 2H, ArH, H‐2‘ and H‐6‘), 8.92 (s, 1H, OH). ^13^C‐NMR (126 MHz, DMSO‐d_6_): (δ) 41.7 (CH_2_), 47.2 (CH_2_), 50.4 (C‐3 and C‐5), 115.5 (C‐2“ and C‐6“), 118.5 (C‐3“ and C‐5“), 122.9 (C‐4‘), 129.2 (C‐2’and C‐6‘), 131.4 (C‐3’and C‐5‘), 135.0 (C‐1‘), 143.8 (C‐1“), 151.4 (C‐4“), 168.0 (C=O). Anal. Calcd for (C_17_H_17_BrN_2_O_2_): C 56.52, H 4.74, N 7.75. Found: C 56.48, H 4.71, N 7.70.

### (4‐(4‐Hydroxyphenyl)piperazin‐1‐yl)(2,4‐dibromophenyl)methanone (26)

Yield: 92 %. Pink powder. M.p. 176–177 °C. R_*f*_: 0.44. ^1^H‐NMR (500 MHz, DMSO‐*d*_6_): (δ) 2.91 (m, 2H, CH_2_), 3.03 (m, 2H, CH_2_), 3.23 (t, *J=5.0 Hz*, 2H, CH_2_), 3.75 (m, 2H, CH_2_), 6.67 (d, *J=8.6 Hz*, 2H, ArH, H‐2“ and H‐6“), 6.79 (d, *J*=8.6 Hz, 2H, ArH, H‐3“ and H‐5“), 7.34 (d, *J*=8.2 Hz, 1H, ArH, H‐5‘), 7.67 (dd, *J*=8.1, *J*=1.8 Hz, 1H, ArH, 6‐H‘), 7.96 (d, *J*=1.7 Hz, 1H, ArH, H‐3‘), 8.90 (s, 1H, OH). ^13^C‐NMR (126 MHz, DMSO‐d_6_): (δ) 41.2 (CH_2_), 46.3 (CH_2_), 50.1 (CH_2_), 50.5 (CH_2_), 115.5 (C‐2“ and C‐6“), 118.5 (C‐3“ and C‐5“), 119.5 (C‐2‘), 122.5 (C‐4‘), 129.5 (C‐6‘), 131.1 (C‐5‘), 134.5 (C‐3‘), 137.1 (C‐1‘), 143.7 (C‐1“), 151.5 (C‐4“), 165.5 (C=O). Anal. Calcd for (C_17_H_16_Br_2_N_2_O_2_): C 46.39, H 3.66, N 6.36. Found: C 46.42, H 3.64, N 6.40.

### [4‐(4‐Hydroxyphenyl)piperazin‐1‐yl](2‐methylphenyl)methanone (14)

CAS Number: 1624460‐70‐3. Yield: 76 %. White powder. M.p. 127–128 °C. R_*f*_: 0.41. ^1^H‐NMR (500 MHz, DMSO‐*d*_6_): (δ) 2.22 (s, 3H, CH_3_), 2.85 (m, 2H, CH_2_), 3.01 (bs, 2H, CH_2_), 3.24 (t, *J=4.9 Hz*, 2H, CH_2_), 3.77 (bs, 2H, CH_2_), 6.66 (d, *J=8.9 Hz*, 2H, ArH, H‐2“ and H‐6“), 6.79 (d, *J=8.9 Hz*, 2H, ArH, H‐3“ and H‐5“), 7.18 (d, *J=7.2 Hz*, 1H, ArH, H‐4‘), 7.24 (d, *J=7.2 Hz*, 2H, ArH, H‐6‘), 7.28 (m, 2H, ArH, H‐3‘ and H‐5‘), 8.91 (s, 1H, OH). ^13^C‐NMR (126 MHz, DMSO‐d_6_): (δ) 18.6 (CH_3_), 41.0 (CH_2_), 46.4 (CH_2_), 50.5 (CH_2_), 50.7 (CH_2_), 115.5 (C‐2“ and C‐6“), 118.6 (C‐3“ and C‐5“), 125.8 (C‐5‘), 128.6 (C‐6‘), 130.2 (C‐3‘ and C‐4‘), 133.7 (C‐1‘), 136.3 (C‐2‘), 143.8 (C‐1“), 151.5 (C‐4“), 168.5 (C=O). Anal. Calcd for (C_18_H_20_N_2_O_2_): C 72.95, H 6.80, N 9.45. Found: C 72.98, H 6.82, N 9.49.

### [4‐(4‐Hydroxyphenyl)piperazin‐1‐yl](3‐methylphenyl)methanone (15)

CAS Number: 1623614‐88‐9. Yield: 88 %. White powder. M.p. 155–156 °C. R_*f*_: 0.41. ^1^H‐NMR (500 MHz, DMSO‐*d*_6_): (δ) 2.34 (s, 3H, CH_3_), 2.95 (m, 6H, 3CH_2_), 3.73 (bs, 2H, CH_2_), 6.66 (d, *J=8.7 Hz*, 2H, ArH, H‐2“ and H‐6“), 6.80 (d, *J=8.7 Hz*, 2H, ArH, H‐3“ and H‐5“), 7.19 (d, *J=7.5 Hz*, 1H, ArH, H‐4‘), 7.22 (bs, 1H, ArH, H‐5‘), 7.27 (d, *J=7.6 Hz*, 1H, ArH, H‐6‘), 7.33 (t, *J=7.5 Hz*, 1H, ArH, H‐2‘), 8.91 (s, 1H, OH). ^13^C‐NMR (126 MHz, DMSO‐d_6_): (δ) 20.9 (CH_3_), 41.6 (CH_2_), 47.2 (CH_2_), 50.5 (C‐3 and C‐5), 115.5 (C‐2“ and C‐6“), 118.5 (C‐3“ and C‐5“), 124.0 (C‐2‘), 127.4 (C‐6‘), 128.3 (C‐5‘), 130.1 (C‐4‘), 135.9 (C‐1‘), 137.8 (C‐3‘), 143.9 (C‐1“), 151.5 (C‐4“), 169.1 (C=O). Anal. Calcd for (C_18_H_20_N_2_O_2_): C 72.95, H 6.80, N 9.45. Found: C 73.00, H 6.85, N 9.52.

### [4‐(4‐Hydroxyphenyl)piperazin‐1‐yl](4‐methylphenyl)methanone (16)

CAS Number: 1023496‐70‐9. Yield: 69 %. White powder. M.p. 162–163 °C. R_*f*_: 0.40. ^1^H‐NMR (500 MHz, DMSO‐*d*_6_): (δ) 2.34 (s, 3H, CH_3_), 2.95 (bs, 6H, 3CH_2_), 3.70 (bs, 2H, CH_2_), 6.67 (d, *J=8.8 Hz*, 2H, ArH, H‐2“ and H‐6“), 6.80 (d, *J=8.9 Hz*, 2H, ArH, H‐3“ and H‐5“), 7.25 (d, *J=7.8 Hz*, 2H, ArH, C‐3‘ and C‐5‘), 7.31 (d, *J=8.0 Hz*, 2H, ArH, C‐2‘ and C‐6‘), 8.91 (s, 1H, OH). ^13^C‐NMR (126 MHz, DMSO‐d_6_): (δ) 20.9 (CH_3_), 41.8 (CH_2_), 47.4 (CH_2_), 50.5 (C‐3 and C‐5), 115.5 (C‐2“ and C‐6“), 118.5 (C‐3“ and C‐5“), 127.1 (C‐2‘ and C‐6‘), 128.9 (C‐3‘ and C‐5‘), 132.9 (C‐1‘), 139.2 (C‐4‘), 143.9 (C‐1“), 151.5 (C‐4“), 169.1 (C=O). Anal. Calcd for (C_18_H_20_N_2_O_2_): C 72.95, H 6.80, N 9.45. Found: C 72.97, H 6.84, N 9.49.

### [4‐(4‐Hydroxyphenyl)piperazin‐1‐yl](2,4‐dimethylphenyl)methanone (27)

CAS Number: 1624460‐80‐5. Yield: 71 %. White powder. M.p. 164–165 °C. R_*f*_: 0.41. ^1^H‐NMR (500 MHz, DMSO‐*d*_6_): (δ) 2.19 (s, 3H, CH_3_), 2.29 (s, 3H, CH_3_), 2.84 (bs, 2H, CH_2_), 3.00 (bs, 2H, CH_2_), 3.24 (t, 2H, *J*=4.6 Hz, CH_2_), 3.76 (bs, 2H, CH_2_), 6.65 (d, *J=9.0 Hz*, 2H, ArH, H‐2“ and H‐6“), 6.79 (d, *J=9.0 Hz*, 2H, ArH, H‐3“ and H‐5“), 7.03–7.06 (m, 2H, ArH, H‐5‘ and H‐6‘), 7.08 (d, *J=4.8 Hz*, 1H, ArH, H‐3‘), 8.88 (s, 1H, OH). ^13^C‐NMR (126 MHz, DMSO‐d_6_): (δ) 18.6 (CH_3_), 20.7 (CH_3_), 41.0 (CH_2_), 46.4 (CH_2_), 50.5 (CH_2_), 50.7 (CH_2_), 115.5 (C‐2“ and C‐6“), 118.5 (C‐3“ and C‐5“), 125.8 (C‐5‘), 126.1 (C‐6‘), 130.7 (C‐3‘), 133.5 (C‐1‘), 133.9 (C‐2‘), 137.9 (C‐4‘), 143.8 (C‐1“), 151.4 (C‐4“), 168.7 (C=O). Anal. Calcd for (C_19_H_22_N_2_O_2_): C 73.52, H 7.14, N 9.03. Found: C 73.48, H 7.11, N 9.01.

### (4‐(4‐Hydroxyphenyl)piperazin‐1‐yl)(2‐(trifluoromethyl)phenyl)methanone (17)

CAS Number: 1993240‐74‐6. Yield: 48 %. White powder. M.p. 152–153 °C. R_*f*_: 0.45. ^1^H‐NMR (500 MHz, DMSO‐*d*_6_): (δ) 2.78–2.92 (m, 2H, CH_2_), 2.95–3.08 (m, 2H, CH_2_), 3.14–3.26 (m, 2H, CH_2_), 3.76 (t, *J*=5.2 Hz, 2H, CH_2_), 6.65 (d, *J*=8.9 Hz, 2H, ArH, H‐2“ and H‐6“), 6.80 (d, *J*=8.9 Hz, 2H, ArH, H‐3“ and H‐5“), 7.50 (d, *J*=7.5 Hz, 1H, ArH, H‐3‘), 7.66 (t, *J*=7.7 Hz, 1H, ArH, H‐4‘), 7.76 (t, *J*=7.5 Hz, 1H, ArH, H‐5‘), 7.82 (d, *J*=7.8 Hz, 1H, ArH, H‐6‘), 8.91 (s, 1H, OH). ^13^C‐NMR (126 MHz, DMSO‐d_6_): (δ) 41.2 (CH_2_), 46.7 (CH_2_), 50.1 (C‐3 and C‐5), 115.5 (C‐2“ and C‐6“), 118.6 (C‐3“ and C‐5“), 123.8 (d, *J*_*C‐F*_=273.6 Hz, CF_3_), 125.3 (d, *J*_*C‐F*_=31.2 Hz, C‐2‘), 126.5 (q, *J*_*C‐F*_=4.3 Hz, C‐3‘),127.5 (C‐6‘), 129.5 (C‐4‘), 132.9 (C‐5‘), 134.7 (q, *J*_*C‐F*_=2.4 Hz, C‐1‘), 143.7 (C‐1“), 151.5 (C‐4“), 166.0 (C=O). Anal. Calcd for (C_18_H_17_F_3_N_2_O_2_): C 61.71, H 4.89, N 8.00. Found: C 61.67, H 4.86, N 8.02.

### (4‐(4‐Hydroxyphenyl)piperazin‐1‐yl)(3‐(trifluoromethyl)phenyl)methanone (18)

CAS Number: 1625575‐92‐9. Yield: 26 %. Light grey powder. M.p. 127–128 °C. R_*f*_: 0.46. ^1^H‐NMR (500 MHz, DMSO‐*d*_6_): (δ) 2.98 (m, 6H, 3CH_2_), 3.76 (bs, 2H, CH_2_), 6.66 (d, *J*=8.8 Hz, 2H, ArH, H‐2“ and H‐6“), 6.80 (d, *J*=8.8 Hz, 2H, ArH, H‐3“ and H‐5“), 7.70 (t, *J*=7.6 Hz, 1H, ArH, H‐5‘), 7.74 (d, *J*=8.1 Hz, 1H, ArH, H‐4‘), 7.77 (s, 1H, ArH, H‐2‘), 7.84 (d, *J*=7.6 Hz, 1H, ArH, H‐6‘), 8.91 (s, 1H, OH). ^13^C‐NMR (126 MHz, DMSO‐d_6_): (δ) 41.7 (CH_2_), 47.2 (CH_2_), 50.3 (C‐3 and C‐5), 115.5 (C‐2“ and C‐6“), 118.5 (C‐3“ and C‐5“), 123.7 (q, *J*_*C‐F*_=3.8 Hz, C‐4‘), 123.9 (d, *J*_*C‐F*_=272.7 Hz, CF_3_), 126.2 (q, *J*_*C‐F*_=3.5 Hz, C‐2‘), 129.3 (d, *J*_*C‐F*_=31.8 Hz, C‐3‘), 129.7 (C‐5‘), 131.0 (C‐6‘), 137.0 (C‐1‘), 143.8 (C‐1“), 151.4 (C‐4“), 167.4 (C=O). Anal. Calcd for (C_18_H_17_F_3_N_2_O_2_): C 61.71, H 4.89, N 8.00. Found: C 61.74, H 4.93, N 7.97.

### (4‐(4‐Hydroxyphenyl)piperazin‐1‐yl)(4‐(trifluoromethyl)phenyl)methanone (19)

CAS Number: 1623717‐66‐7. Yield: 55 %. Light grey powder. M.p. 201–202 °C. R_*f*_: 0.46. ^1^H‐NMR (500 MHz,DMSO‐*d*_6_): (δ) 2.97 (m, 6H, 3CH_2_), 3.77 (bs, 2H, CH_2_), 6.66 (d, *J*=8.9 Hz, 2H, ArH, H‐2“ and H‐6“), 6.81 (d, *J*=8.9 Hz, 2H, ArH, H‐3“ and H‐5“), 7.65 (d, *J*=7.9 Hz, 2H, ArH, H‐3‘ and H‐5‘), 7.82 (d, *J*=8.0 Hz, 2H, ArH, H‐2‘ and H‐6‘), 8.92 (s, 1H, OH). ^13^C‐NMR (126 MHz, DMSO‐d_6_): (δ) 41.7 (CH_2_), 47.1 (CH_2_), 50.2 (CH_2_), 50.5 (CH_2_), 115.5 (C‐2“ and C‐6“), 118.6 (C‐3“ and C‐5“), 123.9 (q, *J*_*C‐F*_=272.5 Hz, CF_3_), 125.5 (q, *J*_C‐F_=3.8 Hz, C‐3‘ and C‐5‘), 127.8 (C‐2‘ and C‐6‘),129.7 (q, *J*_*C‐F*_=32.0 Hz, C‐4‘), 140.0 (C‐1‘), 143.8 (C‐1“), 151.5 (C‐4“), 167.6 (C=O). Anal. Calcd for (C_18_H_17_F_3_N_2_O_2_): C 61.71, H 4.89, N 8.00. Found: C 61.66, H 4.91, N 7.95.

### (4‐(4‐Hydroxyphenyl)piperazin‐1‐yl)(2,4‐bis(trifluoromethyl)phenyl)methanone (20)

Yield: 72 %. White powder. M.p. 194–195 °C. R_*f*_: 0.40. ^1^H‐NMR (500 MHz, DMSO‐*d*_6_): (δ) 2.77–280 (m, 1H), 2.90–2.99 (m, 2H, CH_2_), 3.06–3.11 (m, 1H), 3.16–3.20 (m, 1H), 3.24–3.29 (m, 1H), 3.78 (m, 2H, CH_2_), 6.67 (d, *J=9.0 Hz*, 2H, ArH, H‐2“ and H‐6“), 6.81 (d, *J*=8.09 Hz, 2H, ArH, H‐3“ and H‐5“), 7.81 (d, *J*=8.4 Hz, 1H, ArH, H‐3‘), 8.17 (m, 2H, ArH, H‐5‘ and H‐6‘), 8.93 (s, 1H, OH). ^13^C‐NMR (126 MHz, DMSO‐d_6_): (δ) 41.3 (CH_2_), 46.6 (CH_2_), 50.1 (C‐3 and C‐5), 115.5 (C‐2“ and C‐6“), 118.6 (C‐3“ and C‐5“), 122.9 (d, *J*_C‐F_=273.7 Hz, CF_3_), 123.1 (d, *J*_C‐F_=273.2 Hz, CF_3_), 123.8 (d, *J*_C‐F_=3.7 Hz, C‐3‘), 126.4 (d, *J*_C‐F_=31.4 Hz, C‐2‘), 129.1 (C‐6‘), 130.04 (d, *J*_C‐F_=3.7 Hz, C‐5‘), 129.97 (d, *J*_C‐F_=32.9 Hz, C‐4‘), 138.7 (C‐1‘), 143.7 (C‐1“), 151.5 (C‐4“), 164.7 (C=O). Anal. Calcd for (C_19_H_16_F_6_N_2_O_2_): C 54.55, H 3.86, N 6.70. Found: C 54.61, H 3.87, N 6.75.

### [4‐(4‐Hydroxyphenyl)piperazin‐1‐yl](2‐methoxyphenyl)methanone (21)

CAS Number: 1625821‐37‐5. Yield: 79 %. White powder. M.p. 169–170 °C. R_*f*_: 0.31. ^1^H‐NMR (500 MHz, DMSO‐*d*_6_): (δ) 2.83–2.89 (m, 2H, CH_2_), 2.98 (m, 2H, CH_2_), 3.24 (m, 2H, CH_2_), 3.73 (m, 2H, CH_2_), 3.79 (s, 3H, CH_3_), 6.65 (d, *J=8.7 Hz*, 2H, ArH, H‐2“ and H‐6“), 6.80 (d, *J=8.7 Hz*, 2H, ArH, H‐3“ and H‐5“), 7.00 (t, *J=7.4 Hz*, 1H, ArH, H‐6‘), 7.08 (d, *J=8.4 Hz*, 1H, ArH, H‐4‘), 7.19 (d, *J=*7.3 Hz, 1H, ArH, H‐3‘), 7.40 (m, 1H, ArH, H‐5‘), 8.90 (s, 1H, OH). ^13^C‐NMR (126 MHz, DMSO‐d_6_): (δ) 41.1 (CH_2_), 46.4 (CH_2_), 50.3 (CH_2_), 50.7 (CH_2_), 55.4 (CH_3_), 111.3 (C‐3‘), 115.5 (C‐2“ and C‐6“), 118.5 (C‐3“ and C‐5“), 120.6 (C‐5‘), 125.6 (C‐1‘), 127.7 (C‐6‘), 130.4 (C‐4‘), 143.9 (C‐1“), 151.4 (C‐4“), 154.9 (C‐2‘), 166.4 (C=O). Anal. Calcd for (C_18_H_20_N_2_O_3_): C 69.21, H 6.45, N 8.97. Found: C 69.28, H 6.58, N 9.04.

### [4‐(4‐Hydroxyphenyl)piperazin‐1‐yl](3‐methoxyphenyl)methanone (22)

CAS Number: 1623614‐68‐5. Yield: 69 %. White powder. M.p. 165–166 °C. R_*f*_: 0.33. ^1^H‐NMR (500 MHz, DMSO‐*d*_6_): (δ) 2.96 (m, 6H, 3CH_2_), 3.73 (bs, 2H, CH_2_), 3.78 (s, 3H, CH_3_), 6.66 (d, *J=8.7 Hz*, 2H, ArH, H‐2“ and H‐6“), 6.80 (d, *J=8.7 Hz*, 2H, ArH, H‐3“ and H‐5“), 6.94 (d, *J=2.2 Hz*, 1H, ArH, H‐2‘), 6.96 (d, *J=7.5 Hz*, 1H, ArH, H‐4‘), 7.02 (dd, *J* ^1^ *=*8.3 Hz and *J* ^*2*^=2.6 Hz, 1H, ArH, H‐5‘), 7.36 (t, *J*=7.8 Hz, 1H, ArH, H‐6‘), 8.91 (s, 1H, OH). ^13^C‐NMR (126 MHz, DMSO‐d_6_): (δ) 41.7 (CH_2_), 47.2 (CH_2_), 50.5 (C‐3 and C‐5), 55.2 (CH_3_), 112.3 (C‐2‘), 115.2 (C‐3‘), 115.5 (C‐2“ and C‐6“), 118.5 (C‐3“ and C‐5“), 118.9 (C‐6‘), 129.7 (C‐5‘), 137.3 (C‐1‘), 143.9 (C‐1“), 151.5 (C‐4“), 159.2 (C‐3‘), 168.7 (C=O). Anal. Calcd for (C_18_H_20_N_2_O_3_): C 69.21, H 6.45, N 8.97. Found: C 69.24, H 6.43, N 8.92.

### [4‐(4‐Hydroxyphenyl)piperazin‐1‐yl](4‐methoxyphenyl)methanone (23)

CAS Number: 1024368‐66‐8. Yield: 70 %. White powder. M.p. 177–178 °C. R_*f*_: 0.31. ^1^H‐NMR (500 MHz, DMSO‐*d*_6_): (δ) 2.95 (bs, 4H, 2CH_2_), 3.60 (bs, 4H, 2CH_2_), 3.79 (s, 3H, CH_3_), 6.66 (d, *J=8.9 Hz*, 2H, ArH, H‐2“ and H‐6“), 6.80 (d, *J=8.9 Hz*, 2H, ArH, H‐3“ and H‐5“), 6.99 (d, *J*=8.7 Hz, 2H, ArH, H‐3‘ and H‐5‘), 7.39 (d, *J*=8.8 Hz, 2H, ArH, H‐2‘ and H‐6‘), 8.90 (s, 1H, OH). ^13^C‐NMR (126 MHz, DMSO‐d_6_): (δ) 26.3 (CH_2_), 30.7 (CH_2_), 50.5 (C‐3 and C‐5), 55.2 (CH_3_), 113.6 (C‐3‘ and C‐5‘), 115.5 (C‐2“ and C‐6“), 118.5 (C‐3“ and C‐5“), 127.8 (C‐2‘ and C‐6‘), 129.1 (C‐1‘), 143.9 (C‐1“), 151.4 (C‐4“), 160.2 (C‐4‘), 168.9 (C=O). Anal. Calcd for (C_18_H_20_N_2_O_3_): C 69.21, H 6.45, N 8.97. Found: C 69.18, H 6.41, N 8.95.

### [4‐(4‐Hydroxyphenyl)piperazin‐1‐yl](2,4‐dimethoxyphenyl)methanone (24)

CAS Number: 1024368‐66‐8. Yield: 24 %. Light grey powder. M.p. 174–175 °C. R_*f*_: 0.30. ^1^H‐NMR (500 MHz, DMSO‐*d*_6_): (δ) 2.89 (m, 4H, 2CH_2_), 3.32 (m, 4H, 2CH_2_), 3.79 (s, 6H, 2CH_3_), 6.58 (m, 2H, ArH, H‐3“ and H‐5“), 6.66 (m, 2H, ArH, H‐2“ and H‐6“), 6.80 (d, *J*=7.4 Hz, 2H, ArH, H‐3‘ and H‐5‘), 7.13 (d, *J*=7.8 Hz, 1H, ArH, C‐6‘), 8.89 (s, 1H, OH). ^13^C‐NMR (126 MHz, DMSO‐d_6_): (δ) 41.2 (CH_2_), 46.5 (CH_2_), 50.3 (CH_2_), 50.7 (CH_2_), 55.4 (2CH_3_), 98.3 (C‐3‘), 105.4 (C‐5‘), 115.5 (C‐2“ and C‐6“), 118.1 (C‐3“ and C‐5“), 118.5 (C‐1‘), 128.9 (C‐6‘), 143.9 (C‐1“), 151.4 (C‐4“), 156.3 (C‐2‘), 161.2 (C‐4‘), 166.4 (C=O). Anal. Calcd for (C_19_H_22_N_2_O_4_): C 66.65, H 6.48, N 8.18. Found: C 66.70, H 6.52, N 8.11.

### (4‐(4‐hydroxyphenyl)piperazin‐1‐yl)(2‐nitrophenyl)methanone (28)

CAS Number: 1987456‐19‐8. Yield: 70 %. Orange powder. M.p. 184–185 °C. R_*f*_: 0.35. ^1^H‐NMR (500 MHz, DMSO‐*d*_6_): (δ) 2.90 (s, 2H, CH_2_), 3.06 (s, 2H, CH_2_), 3.30 (s, 2H, CH_2_), 3.77 (s, 2H, CH_2_), 6.66 (d, *J*=8.9 Hz, 2H, ArH, H‐2“ and H‐6“), 6.81 (d, *J*=8.9 Hz, 2H, ArH, H‐3“ and H‐5“), 7.58 (dd, *J*=7.6 Hz, 1H, ArH, H‐5‘), 7.72 (m, 1H, ArH, H‐4‘) 7.86 (td, *J* ^*1*^=7.5 Hz and J^2^=1.1 Hz, 1H, ArH, H‐6‘), 8.21 (dd, *J*=8.3 Hz, 1H, ArH, H‐3‘), 8.89 (s, 1H, OH). ^13^C‐NMR (126 MHz, DMSO‐d_6_): (δ) 41.4 (CH_2_), 46.6 (CH_2_), 50.0 (CH_2_), 50.2 (CH_2_), 115.5 (C‐2“ and C‐6“), 118.6 (C‐3“ and C‐5“), 124.7 (C‐3‘), 128.1 (C‐6‘), 130.3 (C‐4‘), 132.3 (C‐5‘), 134.9 (C‐1‘), 143.8 (C‐1“), 145.4 (C‐2‘), 151.5 (C‐4“), 165.3 (C=O). Anal. Calcd for (C_17_H_17_N_3_O_4_): C 62.38, H 5.23, N 12.84. Found: C 62.34, H 5.20, N 12.80.

### (4‐(4‐Hydroxyphenyl)piperazin‐1‐yl)(3‐nitrophenyl)methanone (29)

CAS Number: 1623717‐58‐7. Yield: 60 %. Brown powder. M.p. 163–164 °C. R_*f*_: 0.36. ^1^H‐NMR (500 MHz, DMSO‐*d*_6_): (δ) 2.93 (s, 2H, CH_2_), 3.05 (s, 2H, CH_2_), 3.44 (s, 2H, CH_2_), 3.78 (s, 2H, CH_2_), 6.67 (d, *J*=8.9 Hz, 2H, ArH, H‐2“ and H‐6“), 6.81 (d, 2H, ArH, H‐3“ and H‐5“), 7.76 (t, *J*=7.6 Hz, 1H, ArH, H‐5‘), 7.89 (d, *J*=7.6 Hz, 1H, ArH, H‐6‘) 8.24 (bs, 1H, ArH, H‐4‘), 8.30–8.032 (m, 1H, ArH, H‐2‘), 8.89 (s, 1H, OH).^13^C‐NMR (126 MHz, DMSO‐d_6_): (δ) 41.8 (CH_2_), 47.2 (CH_2_), 50.2 (CH_2_), 50.4 (CH_2_), 115.5 (C‐2“ and C‐6“), 118.5 (C‐3“ and C‐5“), 121.9 (C‐1‘), 124.3 (C‐4‘), 130.2 (C‐5‘), 133.4 (C‐6‘), 137.4 (C‐1‘), 143.8 (C‐1“), 147.7 (C‐3‘), 151.4 (C‐4“), 166.7 (C=O). Anal. Calcd for (C_17_H_17_N_3_O_4_): C 62.38, H 5.23, N 12.84. Found: C 62.32, H 5.18, N 12.87.

### (4‐(4‐Hydroxyphenyl)piperazin‐1‐yl)(4‐nitrophenyl)methanone (30)

CAS Number: 1624408‐53‐2. Yield: 69 %. Yellow powder. M.p. 157–158 °C. R_*f*_: 0.38. ^1^H‐NMR (500 MHz, DMSO‐*d*_6_): (δ) 2.92 (s, 2H, CH_2_), 3.04 (s, 2H, CH_2_), 3.38 (s, 2H, CH_2_), 3.77 (s, 2H, CH_2_), 6.66 (d, *J*=8.8 Hz, 2H, ArH, H‐2“ and H‐6“), 6.81 (d, *J*=8.8 Hz, 2H, ArH, H‐3“ and H‐5“), 7.70 (d, *J*=8.8 Hz, 2H, ArH, H‐2“ and H‐6“), 8.29 (d, *J*=8.8 Hz, 2H, ArH, H‐3“ and H‐5“), 8.88 (s, 1H, OH).^13^C‐NMR (126 MHz, DMSO‐d_6_): (δ) 41.7 (CH_2_), 47.1 (CH_2_), 50.2 (CH_2_), 50.5 (CH_2_), 115.5 (C‐2“ and C‐6“), 118.6 (C‐3“ and C‐5“), 123.8 (C‐3‘ and C‐5‘), 128.3 (C‐2‘ and C‐6‘), 142.2 (C‐1‘), 143.8 (C‐1“), 147.8 (C‐4‘), 151.5 (C‐4“), 167.0 (C=O). Anal. Calcd for (C_17_H_17_N_3_O_4_): C 62.38, H 5.23, N 12.84. Found: C 62.41, H 5.25, N 12.81.

### (4‐(4‐Hydroxyphenyl)piperazin‐1‐yl)(2,4‐dinitrophenyl)methanone (31)

Yield: 63 %. Orange powder. M.p. 225–226 °C. R_*f*_: 0.40. ^1^H‐NMR (500 MHz, DMSO‐*d*_6_): (δ) 2.88–3.07 (m, 6H, 3CH_2_), 3.79 (bs, 2H, CH_2_), 6.66 (d, *J=8.7 Hz*, 2H, ArH, H‐2“ and H‐6“), 6.81 (d, *J=8.8 Hz*, 2H, ArH, H‐3“ and H‐5“), 7.89 (m, 1H, ArH, H‐6‘), 8.64 (m, 1H, ArH, H‐5‘), 8.85 (m, 1H, ArH, H‐3‘), 8.90 (s, 1H, OH). ^13^C‐NMR (126 MHz, DMSO‐d_6_): (δ) 41.5 (CH_2_), 46.5 (CH_2_), 49.9 (CH_2_), 50.1 (CH_2_), 115.5 (C‐2“ and C‐6“), 118.6 (C‐3“ and C‐5“), 120.4 (C‐3‘), 129.2 (C‐6‘), 130.0 (C‐5‘), 137.5 (C‐1‘), 143.7 (C‐1“), 145.5 (C‐2‘), 147.6 (C‐4‘), 151.5 (C‐4“), 163.6 (C=O). Anal. Calcd for (C_17_H_16_N_4_O_6_): C 54.84, H 4.33, N 15.05. Found: C 54.79, H 4.36, N 15.11.

### General procedure for the synthesis of aminophenyl derivatives 32–35

The appropriate nitro‐derivatives **28**–**31** (0.45 mmol) was dissolved in a solution of EtOH (8 ml) and HCl conc. (2 ml), then zinc dust (14.85 mmol) was added in several portions at 0 °C. When the addition was completed, the reaction was refluxed for 2 hours. The resulting mixture was cooled at room temperature, made alkaline with NaOH 5 N aqueous solution and extracted with EtOAc (3×15 ml). The organic phase was dried over Na_2_SO_4_ and evaporated to remove the solvent. The residue was crystallized from Et_2_O/EtOH.

### (4‐(4‐Hydroxyphenyl)piperazin‐1‐yl)(2‐aminophenyl)methanone (32)

CAS Number: 1604099‐56‐0. Yield: 45 %. Light brown powder. M.p. 101–102 °C. R_*f*_: 0.27. ^1^H‐NMR (500 MHz, DMSO‐*d*_6_): (δ) 2.96 (bs, 4H, 2CH_2_), 3.58 (bs, 4H, 2CH_2_), 5.17 (s, 2H, NH_2_), 6.58 (t, *J=7.3 Hz*, 1H, ArH, H‐2“ and H‐6“), 6.67 (d, *J=8.6 Hz*, 2H, ArH, H‐3“ and H‐5“), 6.73 (d, *J=8.1 Hz*, 1H, ArH, H‐3‘), 6.80 (d, *J=8.6 Hz*, 2H, ArH, H‐5‘), 7.02 (d, *J=7.5 Hz*, 1H, ArH, H‐4‘), 7.10 (t, *J=7.6 Hz*, 1H, ArH, H‐6‘), 8.90 (s, 1H, OH). ^13^C‐NMR (126 MHz, DMSO‐d_6_): (δ) 44.1 (C‐2 and C‐6), 50.5 (C‐3 and C‐5), 115.5 (C‐2“ and C‐6“), 115.7 (C‐3‘ and C‐5‘), 118.5 (C‐3“ and C‐5“), 119.2 (C‐1‘), 127.8 (C‐6‘), 130.0 (C‐4‘), 143.9 (C‐1“), 145.8 (C‐2‘), 151.4 (C‐4“), 168.6 (C=O). Anal. Calcd for (C_17_H_19_N_3_O_2_): C 68.67, H 6.44, N 14.13. Found: C 68.61, H 6.47, N 14.15.

### (4‐(4‐Hydroxyphenyl)piperazin‐1‐yl)(3‐aminophenyl)methanone (33)

CAS Number: 1837524‐47‐6. Yield: 48 %. Brown powder. M.p. 95–96 °C. R_*f*_: 0.25. ^1^H‐NMR (500 MHz, DMSO‐*d*_6_): (δ) 2.94 (bs, 4H, 2CH_2_), 3.46 (bs, 2H, CH_2_), 3.69 (bs, 2H, CH_2_), 5.24 (s, 2H, NH_2_), 6.49 (d, *J=7.4 Hz*, 1H, ArH, H‐4‘), 6.57 (s, 1H, ArH, H‐2‘), 6.61 (dd, *J* ^*1*^=8.1 Hz, *J* ^*2*^=1.3 Hz, 1H, ArH, C‐6‘), 6.66 (d, *J=8.9 Hz*, 2H, ArH, H‐2“ and H‐6“), 6.81 (d, *J=8.9 Hz*, 2H, ArH, H‐3“ and H‐5), 7.06 (t, *J=7.7 Hz*, 1H, ArH, H‐5‘), 8.89 (s, 1H, OH). ^13^C‐NMR (126 MHz, DMSO‐d_6_): (δ) 41.5 (CH_2_), 47.1 (CH_2_), 50.8 (C‐3 and C‐5), 112.4 (C‐2‘), 114.3 (C‐4‘), 115.1 (C‐6‘), 115.5 (C‐2“ and C‐6“), 118.7 (C‐3“ and C‐5“), 128.9 (C‐5‘), 136.5 (C‐1‘), 143.6 (C‐1“), 148.2 (C‐3‘), 151.7 (C‐4“), 169.6 (C=O). Anal. Calcd for (C_17_H_19_N_3_O_2_): C 68.67, H 6.44, N 14.13. Found: C 68.70, H 6.40, N 14.09.

### (4‐(4‐Hydroxyphenyl)piperazin‐1‐yl)(4‐aminophenyl)methanone (34)

CAS Number: 1834093‐26‐3. Yield: 44 %. Light brown powder. M.p. 94–95 °C. R_*f*_: 0.21. ^1^H‐NMR (500 MHz, DMSO‐*d*_6_): (δ) 2.94 (m, 4H, 2CH_2_), 3.61 (bs, 4H, 2CH_2_), 5.50 (s, 2H, NH_2_), 6.55 (d, *J=8.1 Hz*, 2H, ArH, H‐2“ and H‐6“), 6.65 (d, *J=8.6 Hz*, 2H, ArH, H‐3“ and H‐5“), 6.80 (d, *J=8.7 Hz*, 2H, ArH, H‐3‘ and H‐5‘), 7.15 (d, *J=8.2 Hz*, 2H, ArH, H‐2‘ and H‐6‘), 8.86 (s, 1H, OH). ^13^C‐NMR (126 MHz, DMSO‐d_6_): (δ) 45.7 (C‐2 and C‐6), 50.6 (C‐3 and C‐5), 112.7 (C‐3‘ and C‐5‘), 115.5 (C‐2“ and C‐6“), 118.5 (C‐3“ and C‐5“), 121.9 (C‐1‘), 129.3 (C‐2‘ and C‐6‘), 143.6 (C‐1“), 150.5 (C‐4‘), 151.4 (C‐4“), 169.9 (C=O). Anal. Calcd for (C_17_H_19_N_3_O_2_): C 68.67, H 6.44, N 14.13. Found: C 68.64, H 6.46, N 14.11.

### (4‐(4‐Hydroxyphenyl)piperazin‐1‐yl)(2,4‐diaminophenyl)methanone (35)

Yield: 40 %. Brown powder. M.p. 80–81 °C. R_*f*_: 0.14. ^1^H‐NMR (500 MHz, DMSO‐*d*_6_): (δ) 2.94 (bs, 4H, 2CH_2_), 3.57 (bs, 4H, 2CH_2_), 5.18 (d, *J*=8.4 Hz, 4H, 2NH_2_), 5.76–5.84 (m, 2H, ArH, H‐5‘ and H‐6‘), 5.87 (bs, 1H, ArH, H‐3‘), 6.65 (d, *J=8.6 Hz*, 2H, ArH, H‐2“ and H‐6“), 6.76–7.81 (m, 2H, ArH, H‐3“ and 5“), 8.86 (s, 1H, OH). ^13^C‐NMR (126 MHz, DMSO‐d_6_): (δ) 44.9 (C‐2 and C‐6), 50.6 (C‐3 and C‐5), 99.5 (C‐3‘), 102.9 (C‐1‘), 106.3 (C‐5‘), 115.5 (C‐2“ and C‐6“), 118.4 (C‐3“ and C‐5“), 129.9 (C‐6‘), 144.0 (C‐1“), 148.9 (C‐4‘), 150.9 (C‐2‘), 151.3 (C‐4“), 170.4 (C=O). Anal. Calcd for (C_17_H_20_N_4_O_2_): C 65.37, H 6.45, N 17.94. Found: C 65.42, H 6.43, N 17.90.

### Mushroom tyrosinase inhibition and kinetic assays

Mushroom tyrosinase (EC 1.14.18.1) was purchased from Sigma Chemical Co. (St. Louis, MO, USA). Tyrosinase inhibition assays and kinetic analysis were performed as previously described.^[19]^ For experimental details see Supporting Information.

### Docking studies

The AbTYR was previously prepared by means of Discovery Studio Visualizer V20.1.0.19295:^[36]^ we removed water molecules, the ligand and added hydrogens to the protein.

The hTYR was built by homology modelling using the web tool SWISS MODEL^[37]^ as reported in our previous work.^[13]^

Ligands structures preparation was carried out using the VEGA ZZ program.^[38]^ The molecules were built and submitted to optimization protocol by AMMP calculation choosing Conjugate gradients minimization; therefore, we performed a conformational search by AMMP in order to obtain the lowest energy conformation. We selected the Boltzmann‐jump method as search parameter and considered the flexible torsions.

Docking studies were carried out using three different programs: Gold V2020.2.0,^[22]^ Glide V8.8[^23^, ^24^, ^25^] and AutoDock V4.2.6.[^26^, ^27^] For all procedures we have set the following parameters: (i) the coordinates x, y and z, respectively −10.021, −28.823 −43.596, of the tropolone's centre as centroid, (ii) a binding cavity of 10 Å in the 3D direction from the original position of the ligand in the reference X‐ray complex (iii) and the ligands were submitted to 100 runs of the docking algorithm specific for each program.

In the docking studies performed with Gold suite we employed the procedure described in our previous paper.^[15]^ In the docking analysis carried out with Glide, the crystal structures of both proteins were additionally minimized by means of the Protein Preparation Wizard^[39]^ implemented in the Maestro software using default parameters. The active site was delineated generating a receptor grid with the 3D coordinates and size box previously described. The XP (extra precision) method was used as fitness function for calculation and no constrains were applied.

To perform a molecular docking using AutoDock, the proteins and the ligands were previously prepared employing AutoDock Tools.^[27]^ The utility assigns the Gasteiger charges and the atom types, defines the torsion angle in the ligand and generates the grid box for docking analysis.

Then after AutoDock was used to dock the compounds setting default criteria and Lamarckian genetic algorithm as search parameter.

The hierarchical clustering procedure was computed through the utility rms_analysis of Gold suite. We employed the group average method as cluster algorithm and selected the poses with an RMSD cut‐off of 2 Å.

Thus, we considered the poses with the same orientation in the binding site predicted by different docking methods. In order to retrieve the best pose from each cluster, the poses are submitted to a rescoring procedure by means Gold program; Chem Score was chosen as scoring function. The ligand‐protein complexes selected from each cluster were further minimized by means of the Prime Refine Protein‐Ligand Complex tool implemented in Schrödinger suite. In the refine calculation were included the residues within 5 Å of ligand, the VSGB was used as solvation model and OPLS3E as force field.^[40]^ The minimization was performed using Monte Carlo as sampling algorithm of the refinement region. The minimized complexes were chosen for analysis and representation.

### Cell culture

Murine melanoma B16F10 cells (CRL‐6475) were cultured as previous reported.^[17]^

### Cell viability analysis

3‐(4,5‐Dimethylthiazol‐2‐yl)‐2,5‐diphenyltetrazolium bromide (MTT) was used to measure the cells viability. Cells were seeded in 96‐well plates for 24 hours with a density of 5x10^3^ for well. Cells were then treated for 48 h with compounds (ranging from 4 μM to 100 μM). After incubation at 37 °C and 5 % of CO_2_, 100 μL of MTT reagent were added to the cells and incubated 3 h at 37 °C. After adding DMSO the absorbance was determined at 560 nm. The amount of living cells in treated samples relative to untreated controls (100 %) provided the cell viability. The mean value and standard deviation (SD) were calculated from triplicate experiments.

### Tyrosinase zymography (L‐DOPA staining)

A day after seeding the B16F10 cells (6×10^4^ cells/mL) the culture medium was substituted by fresh one supplemented with 100 nM α‐MSH. Different concentrations of the compounds (0–25 μM) was added to cells and incubated for 48 hours. Cells treated with 100 nM α‐MSH and 25 μM kojic acid were used as positive control to compare the inhibitory strength of the inhibitors.

After incubation for 48 h, cells were washed and lysed using phosphate buffer (25 mM, pH 6.8) containing Triton X‐100 (1 %) and phenylmethyl‐sulfonyl fluoride (0.1 mM). Cellular lysates were centrifugated at 13000 rpm for 20 min at 4 °C. The protein content was determined by Bradford method and BSA was used as standard.

SDS‐polyacrylamide gel electrophoresis was used to resolve the protein extracts previously mixed with 10 mM Tris‐HCl buffer, pH 7.0, containing 1 % SDS, without mercaptoethanol.

After running, gel was rinsed in phosphate buffer (0.1 M, pH 7.0) and equilibrated for 30 min twice. Then, staining solution containing phosphate buffer (25 mM, pH 6.8) and L‐DOPA (5 mM) was added to the gel and incubated in the dark overnight at 37 °C. Tyrosinase activity was visualized in the gel as dark melanin‐containing bands

### Antioxidant assay

ABTS [2,20‐azinobis‐(3‐ethylbenzothiazoline‐6‐sulfonic acid)] assay was used to determine the total free radical‐scavenging capacity of compounds. 6‐hydroxy‐2,5,7,8‐tetramethylchromane‐2‐carboxylic acid (Trolox) was used as the reference antioxidant.^[15]^

The ABTS⋅^+^ method is based on the capacity of an antioxidant to scavenge the free ABTS⋅^+^. To produce this radical an aqueous mixture of 7 mM ABTS with 2.45 mM potassium persulfate was required.

The ABTS solution was kept in the dark, at room temperature, for 24 h before use. The ABTS⋅^+^ mixture was diluted, and each compound (10 μL) was added to solution. After 1 min of incubation the absorbance was recorded at 734 nm. Afterwards the decrease in A734 was calculated and referred to the Trolox standard curve. Antioxidant activity was expressed as concentration of the compound to give a 50 % reduction in the original absorbance (EC_50_).

## Conflict of interest

The authors declare no conflict of interest.

1

## Supporting information

As a service to our authors and readers, this journal provides supporting information supplied by the authors. Such materials are peer reviewed and may be re‐organized for online delivery, but are not copy‐edited or typeset. Technical support issues arising from supporting information (other than missing files) should be addressed to the authors.

Supporting Information

## Data Availability

The data that support the findings of this study are available in the supplementary material of this article.
