# Supplementary material for: Design, Synthesis, and in Vitro Evaluation of 4‐(4‐Hydroxyphenyl)piperazine‐Based Compounds Targeting Tyrosinase
Source: ChemMedChem. 2022 Sep 26;17(21):e202200305. doi: 10.1002/cmdc.202200305 (PMC9828493; doi:10.1002/cmdc.202200305)
Supplement: Supplementary file 1 — Supporting Information [file CMDC-17-0-s001.pdf]

ChemMedChem

Supporting Information

Design, Synthesis, and in Vitro Evaluation of 4-(4-Hydroxyphenyl)piperazine-Based Compounds Targeting Tyrosinase

Salvatore Mirabile, Maria Paola Germanò, Antonella Fais, Lisa Lombardo, Federico Ricci, Sonia Floris, Anna Cacciola, Antonio Rapisarda, Rosaria Gitto, and Laura De Luca*

Supporting Information

Content:

Figure S1-S68: ^{1}H-NMR and ^{13}C-NMR spectra of compounds **2-35**...S2

Table S1: Smiles strings for compounds **2-35**...S36

Mushroom tyrosinase inhibition assay..S37

Figure S69: Inhibition effects of compounds **7, 10 11, 17** and **21** (40 µM) on monophenolase activity of AbTYR compared to **KA**...S37

Kinetic analysis of the tyrosinase inhibition..S37

Figure S70: 2 D interaction diagrams of compounds **7**, **10**,**11**, **17** and **21** in the catalytic cavity of AbTYR..S38

Figure S71: Cell viability of compounds **7**, **10**, **11**, **17** and **21** on B16F10 cells.................................S41

^{1}H-NMR and ^{13}C-NMR spectra of compounds 2-35

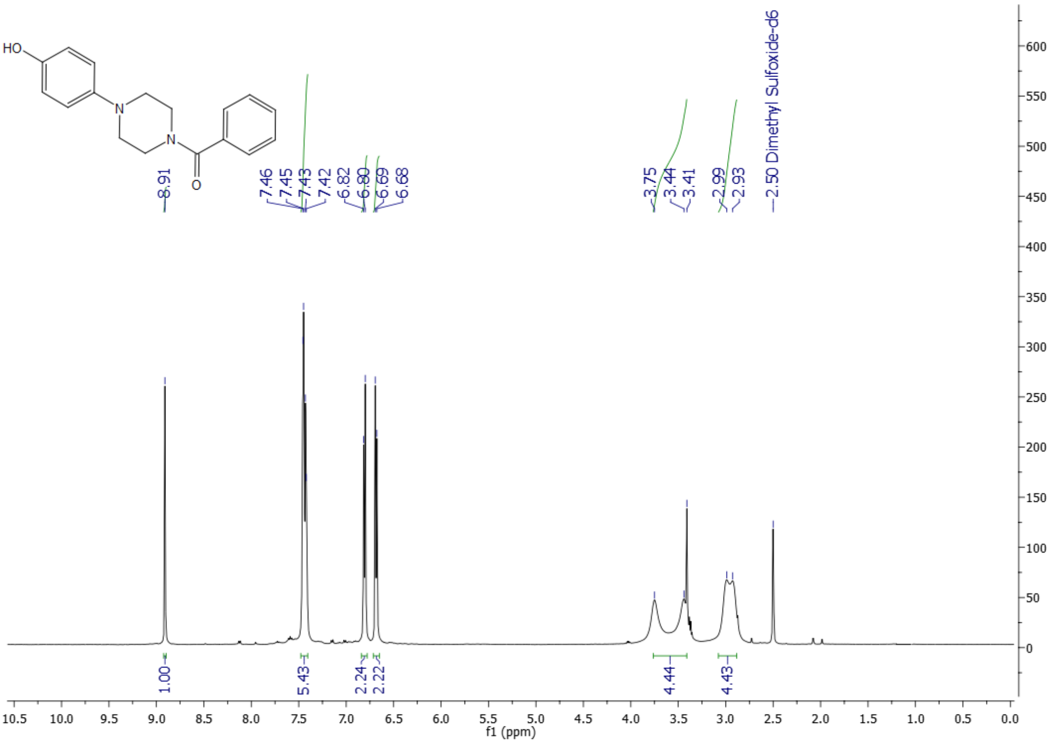

Figure S1: ^{1}H-NMR (DMSO-d_6) spectrum of (4-(4-hydroxyphenyl)piperazin-1-yl)(phenyl)methanone **(2)**

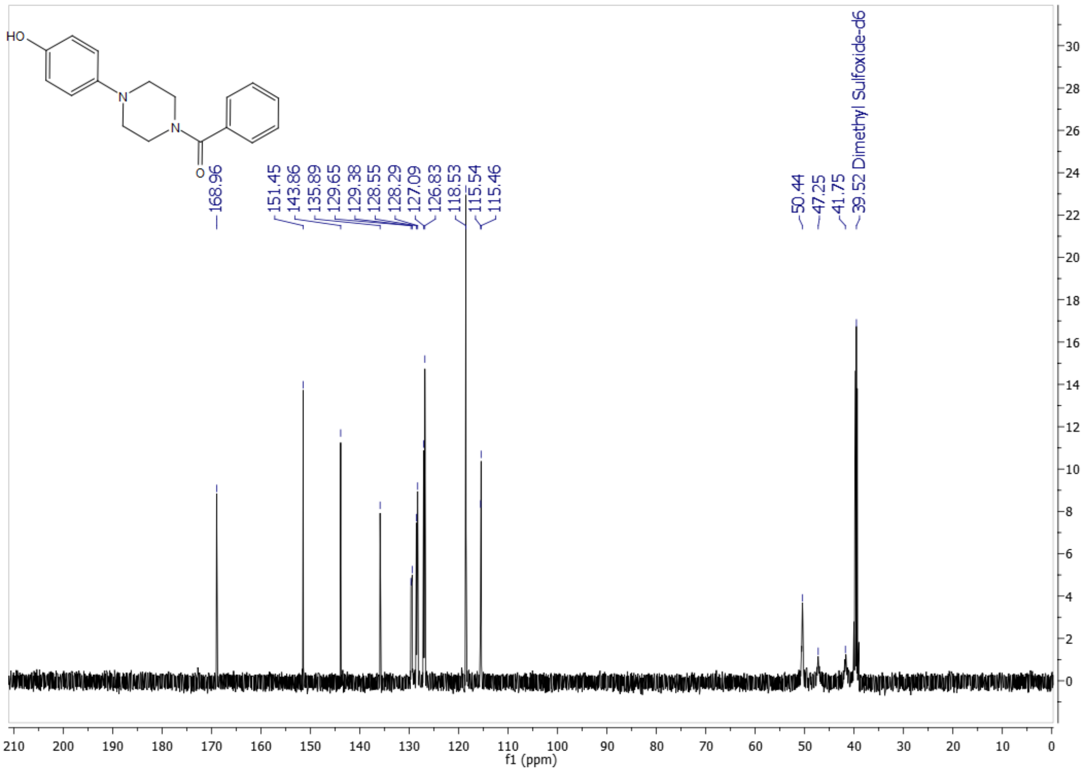

Figure S2: ^{13}C-NMR (DMSO-d_6) spectrum of (4-(4-hydroxyphenyl)piperazin-1-yl)(phenyl)methanone **(2)**

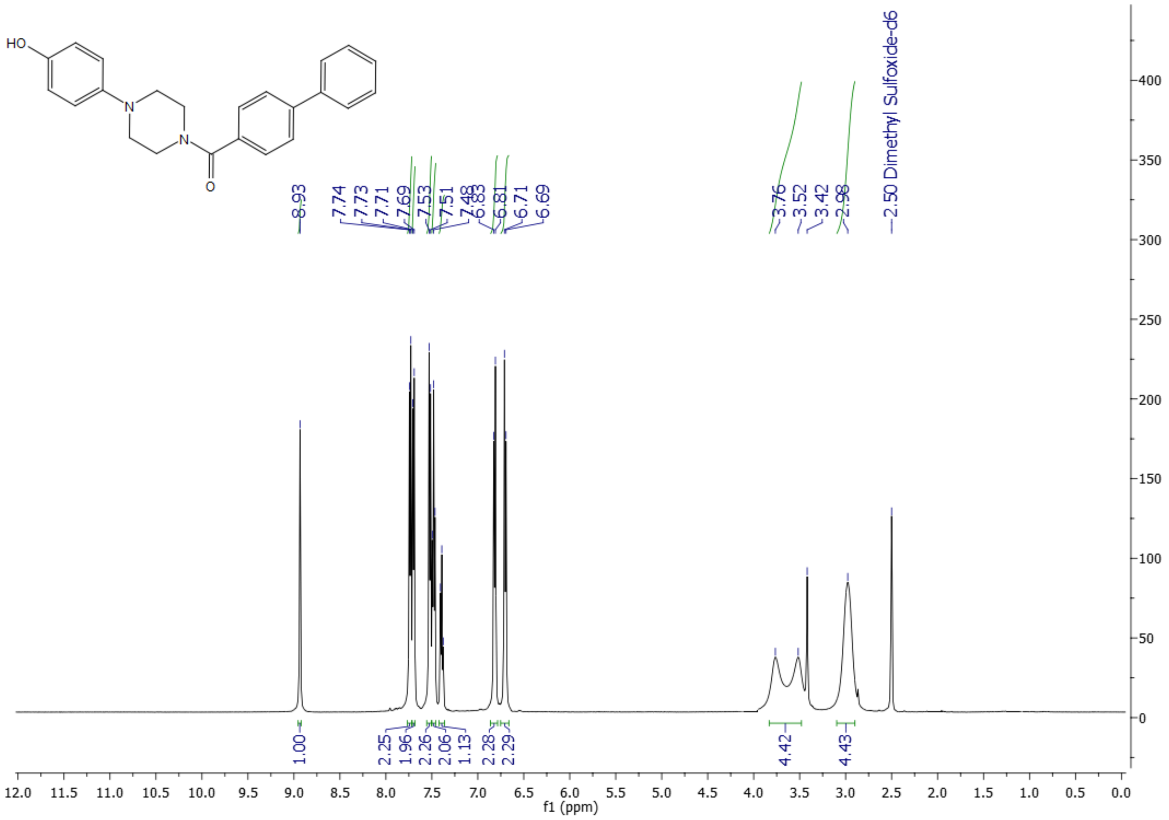

Figure S3: ^{1}H-NMR (DMSO-d$_6$) spectrum of (4-(4-hydroxyphenyl)piperazin-1-yl)(biphenyl-4-yl)methanone **(3)**

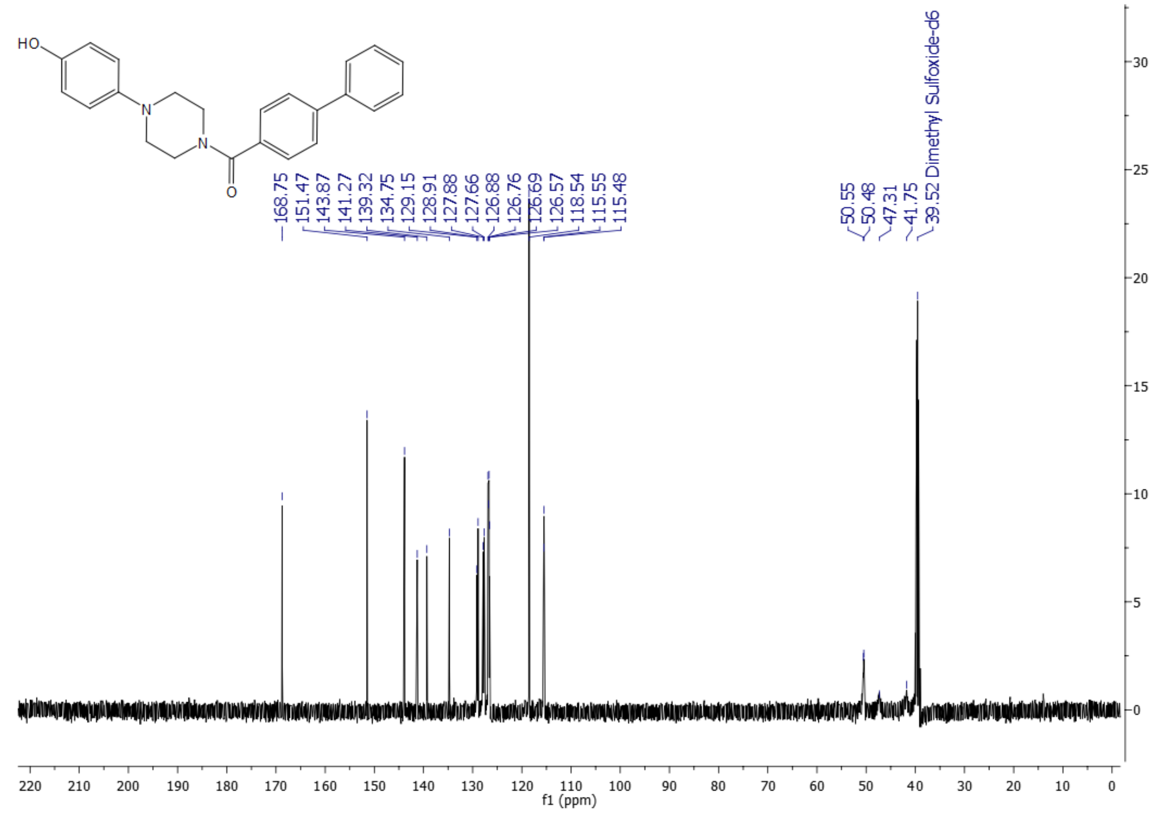

Figure S4: ^{13}C-NMR (DMSO-d$_6$) spectrum of (4-(4-hydroxyphenyl)piperazin-1-yl)(biphenyl-4-yl)methanone **(3)**

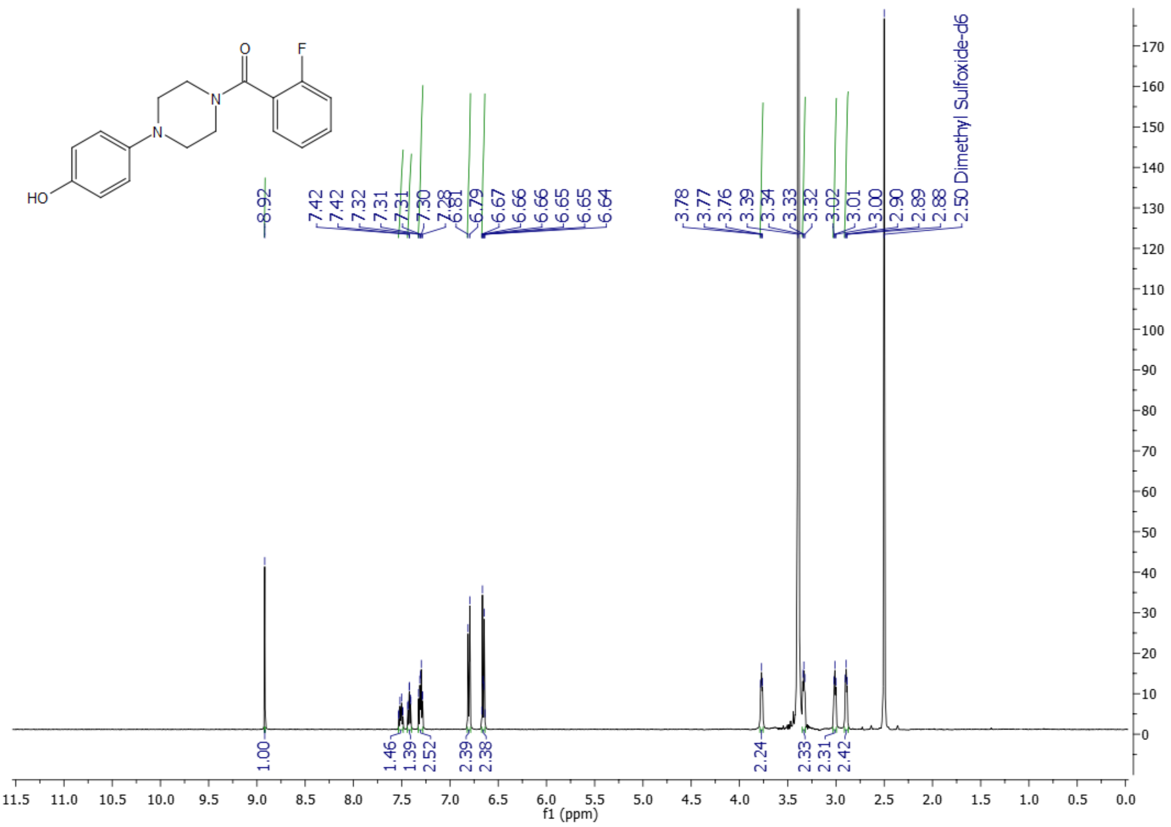

Figure S5: ^{1}H-NMR (DMSO-d_6) spectrum of (4-(4-hydroxyphenyl)piperazin-1-yl)(2-fluorophenyl)methanone **(4)**

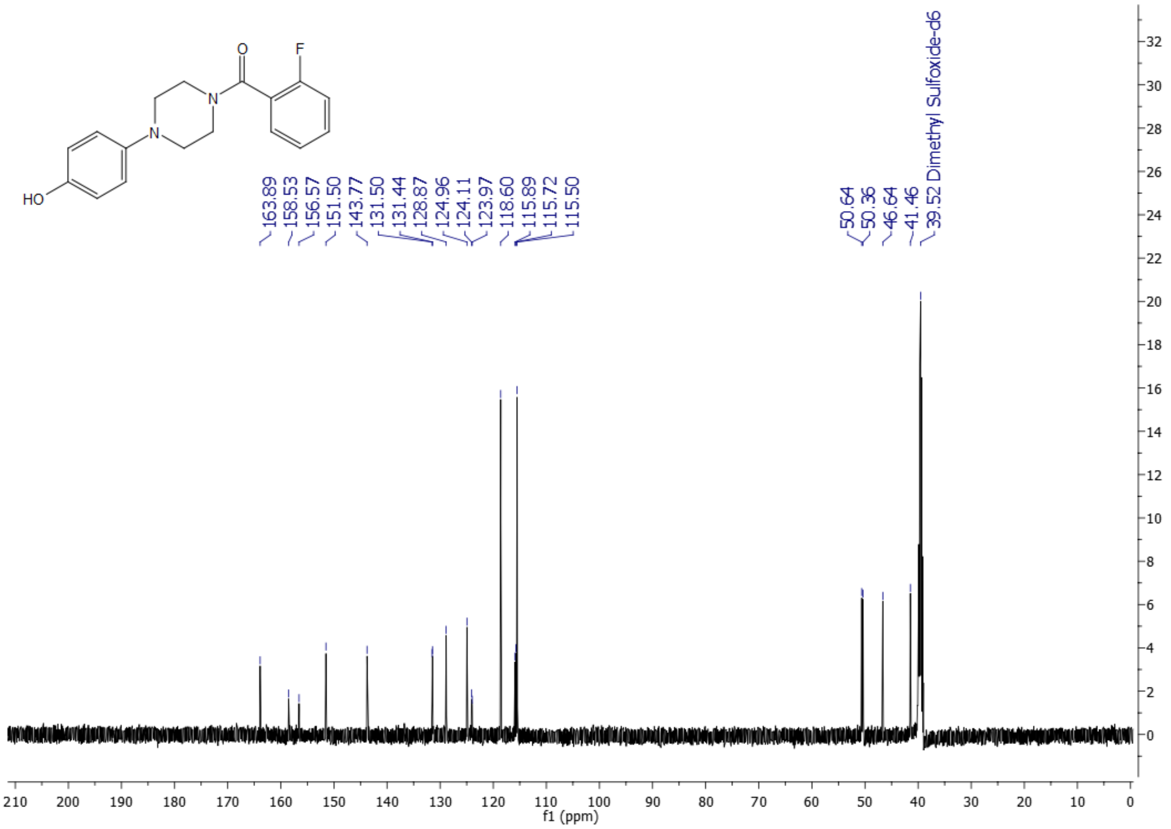

Figure S6: ^{13}C-NMR (DMSO-d_6) spectrum of (4-(4-hydroxyphenyl)piperazin-1-yl)(2-fluorophenyl)methanone **(4)**

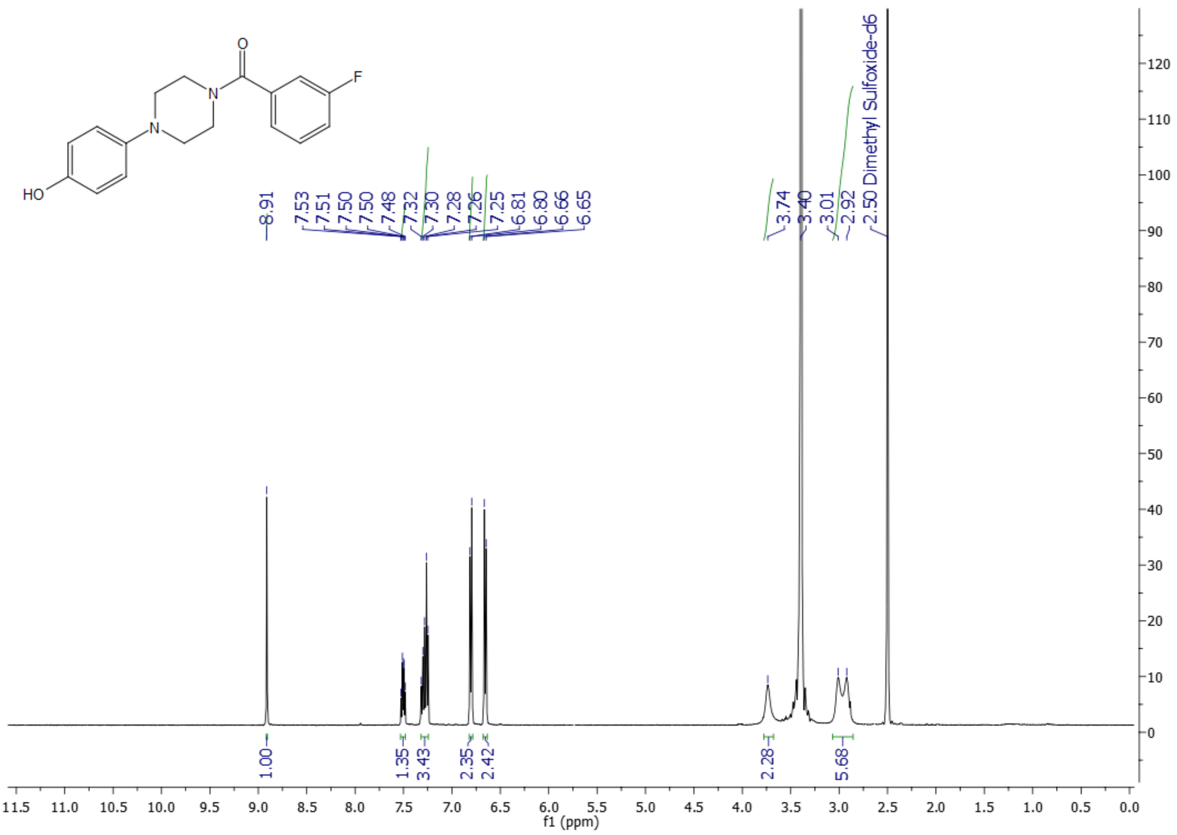

Figure S7: ^{1}H-NMR (DMSO-d_6) spectrum of (4-(4-hydroxyphenyl)piperazin-1-yl)(3-fluorophenyl)methanone **(5)**

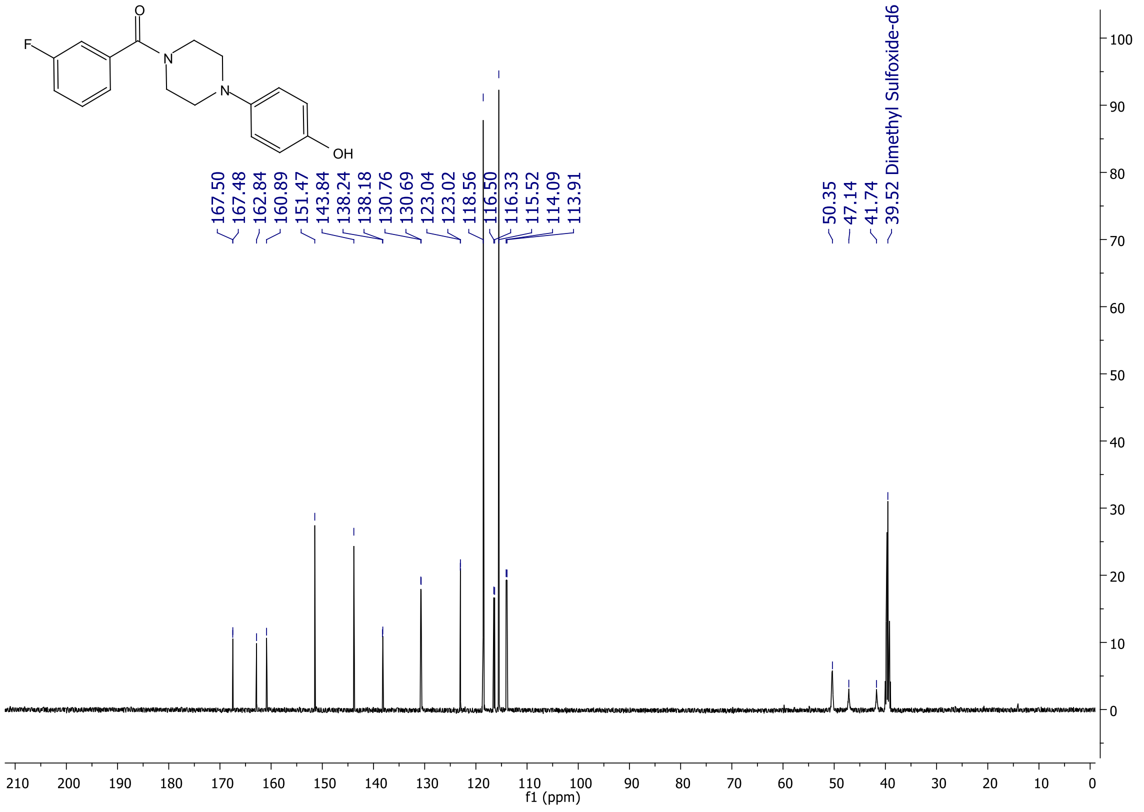

Figure S8: ^{13}C-NMR (DMSO-d_6) spectrum of (4-(4-hydroxyphenyl)piperazin-1-yl)(3-fluorophenyl)methanone **(5)**

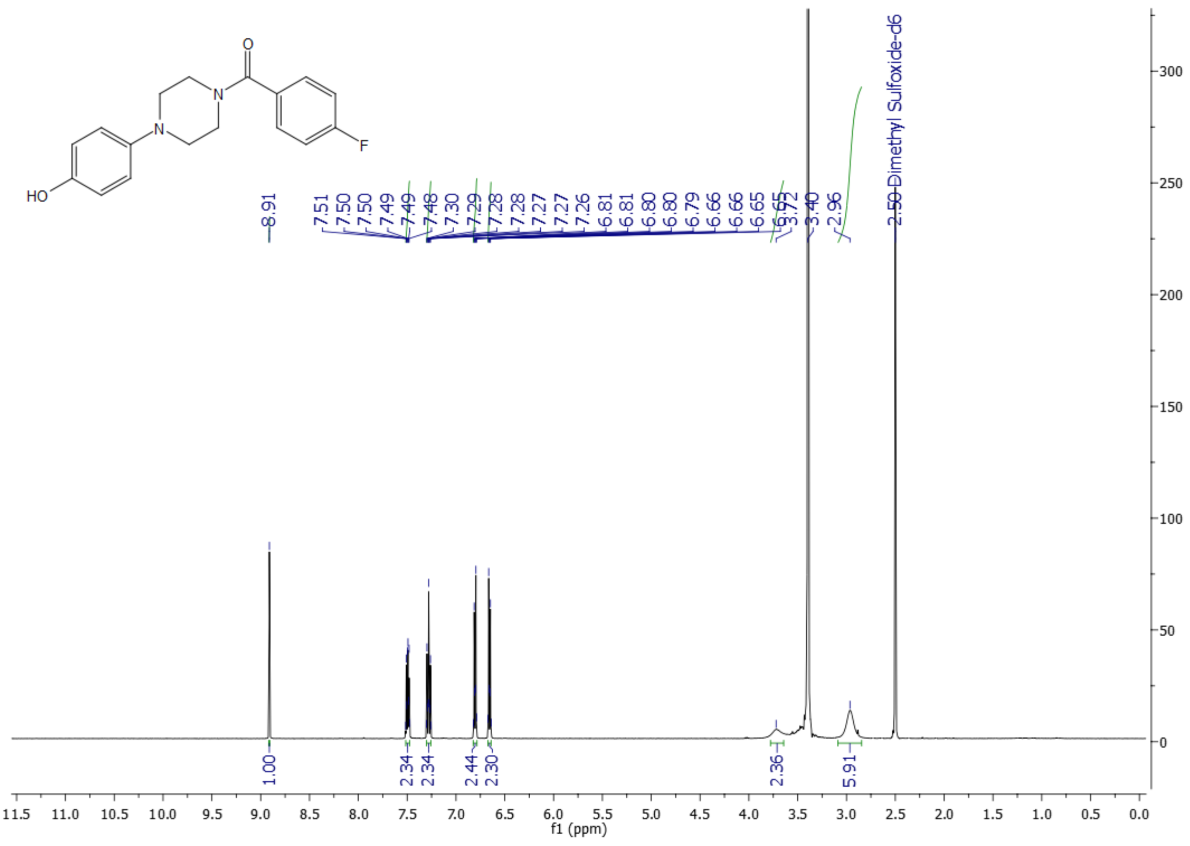

Figure S9: ^{1}H-NMR (DMSO-d$_6$) spectrum of (4-(4-hydroxyphenyl)piperazin-1-yl)(4-fluorophenyl)methanone **(6)**

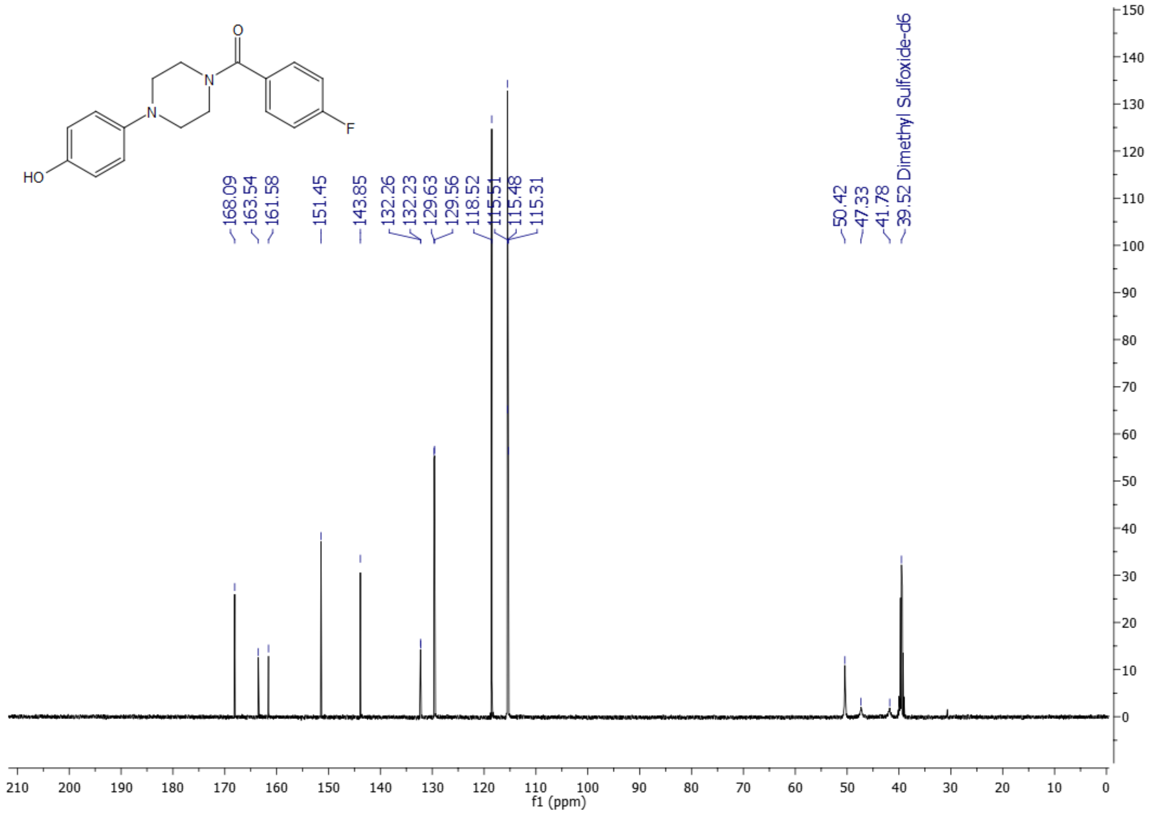

Figure S10: ^{13}C-NMR (DMSO-d$_6$) spectrum of (4-(4-hydroxyphenyl)piperazin-1-yl)(4-fluorophenyl)methanone **(6)**

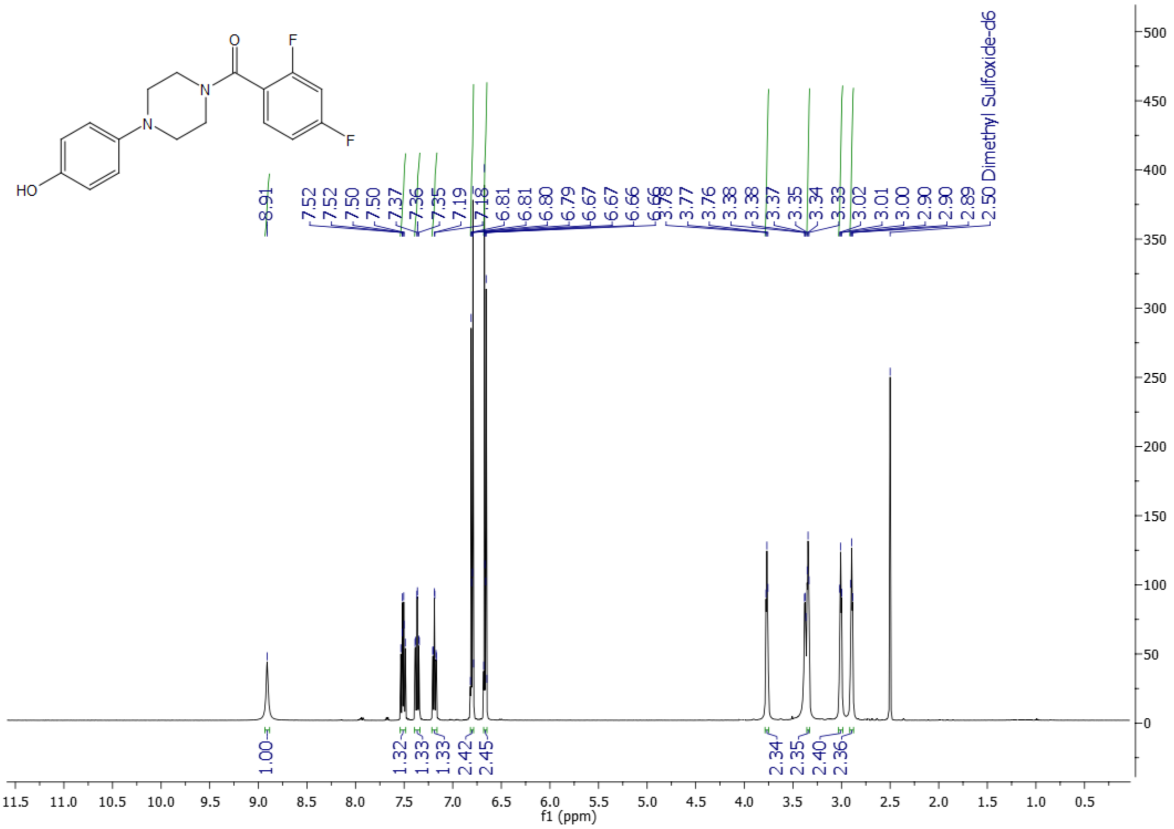

Figure S11: ^{1}H-NMR (DMSO-d$_6$) spectrum of (4-(4-hydroxyphenyl)piperazin-1-yl)(2,4-difluorophenyl)methanone **(25)**

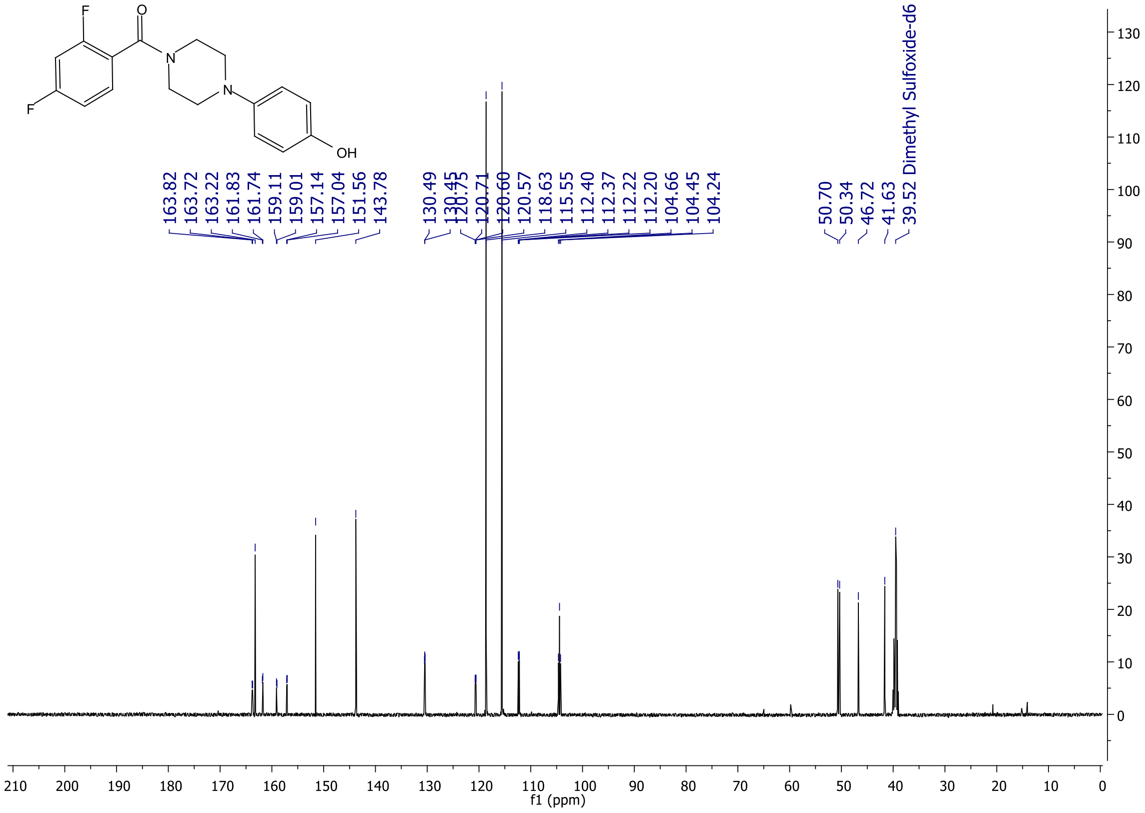

Figure S12: ^{13}C-NMR (DMSO-d$_6$) spectrum of (4-(4-hydroxyphenyl)piperazin-1-yl)(2,4-difluorophenyl)methanone **(25)**

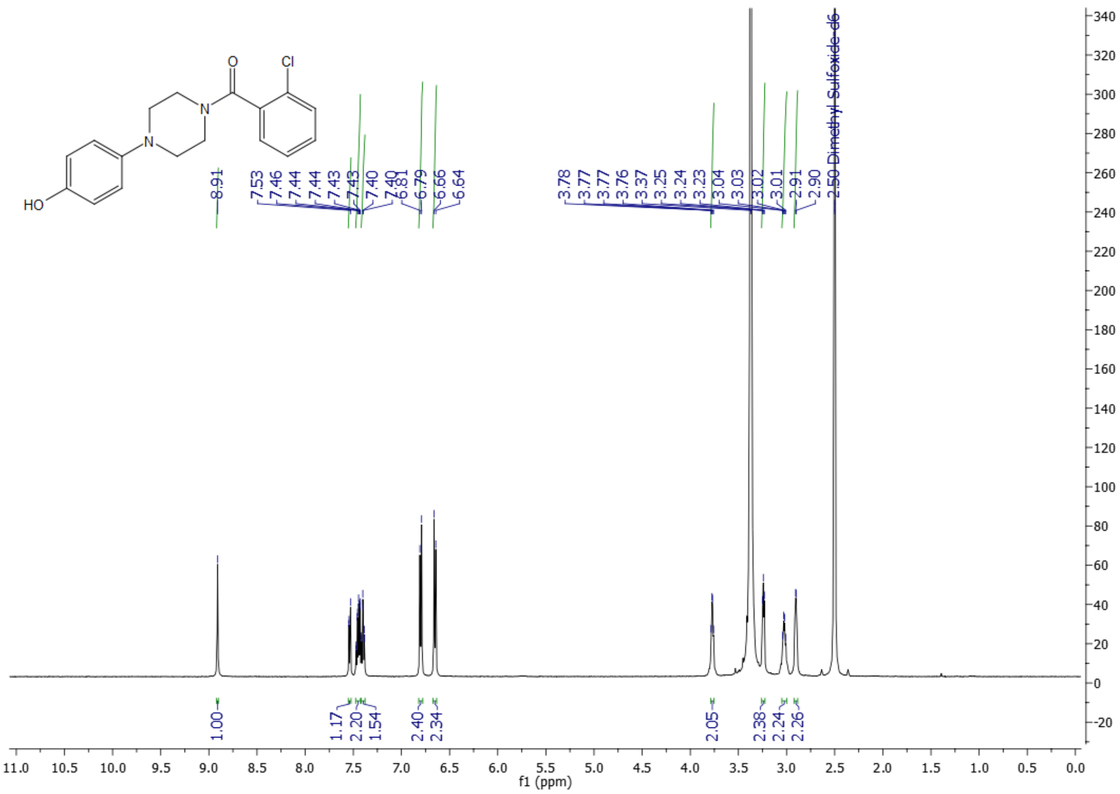

Figure S13: ^{1}H-NMR (DMSO-d_6) spectrum of (4-(4-hydroxyphenyl)piperazin-1-yl)(2-chlorophenyl)methanone **(7)**

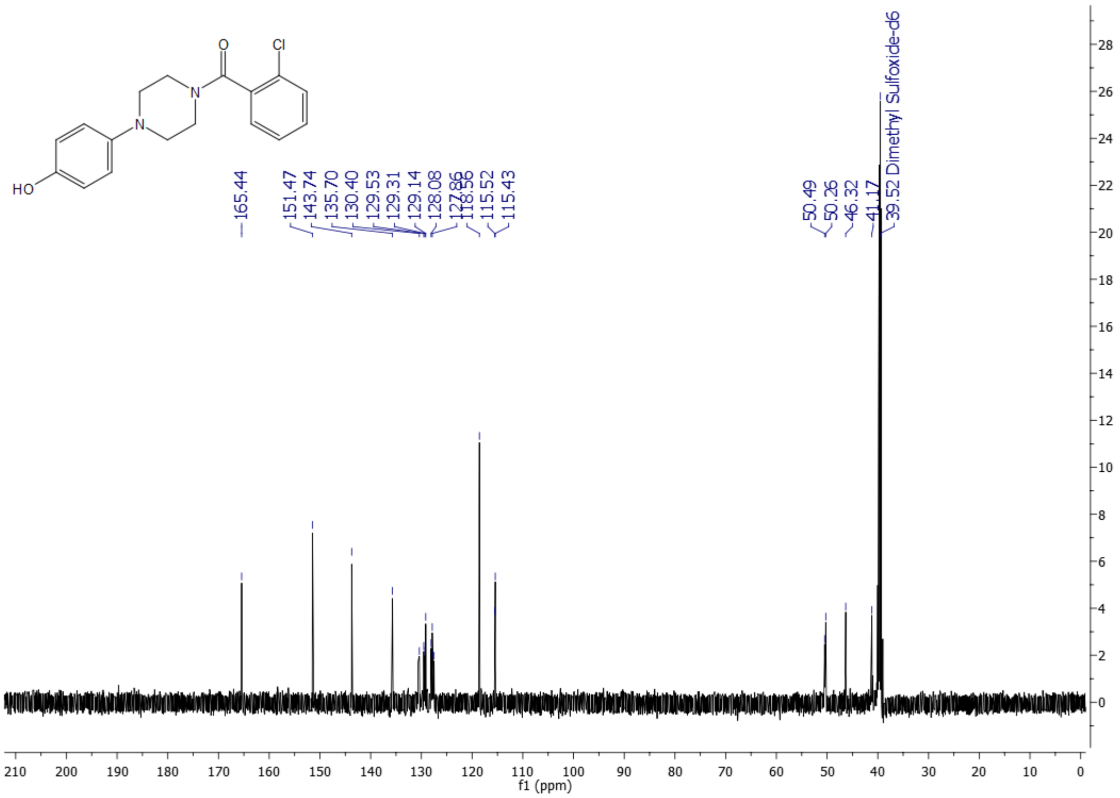

Figure S14: ^{13}C-NMR (DMSO-d_6) spectrum of (4-(4-hydroxyphenyl)piperazin-1-yl)(2-chlorophenyl)methanone **(7)**

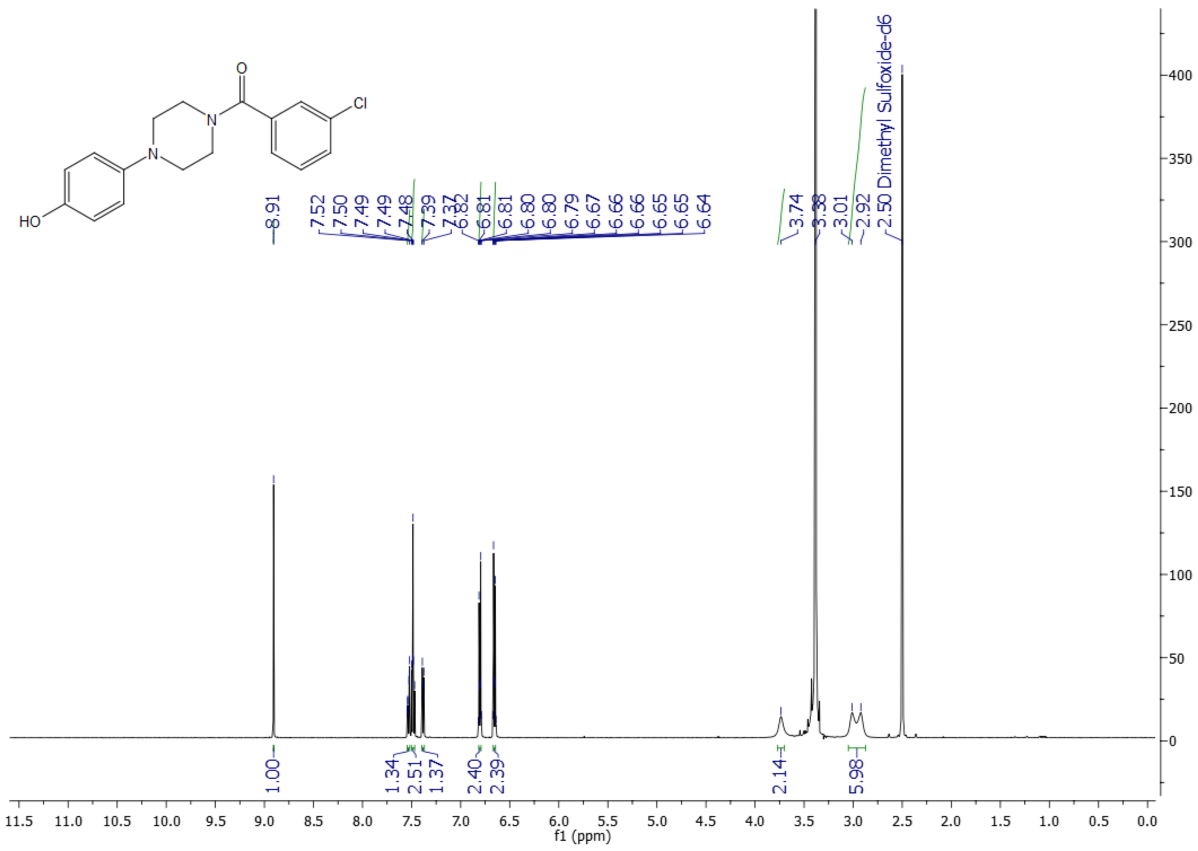

Figure S15: ^{1}H-NMR (DMSO-d$_6$) spectrum of (4-(4-hydroxyphenyl)piperazin-1-yl)(3-chlorophenyl)methanone **(8)**

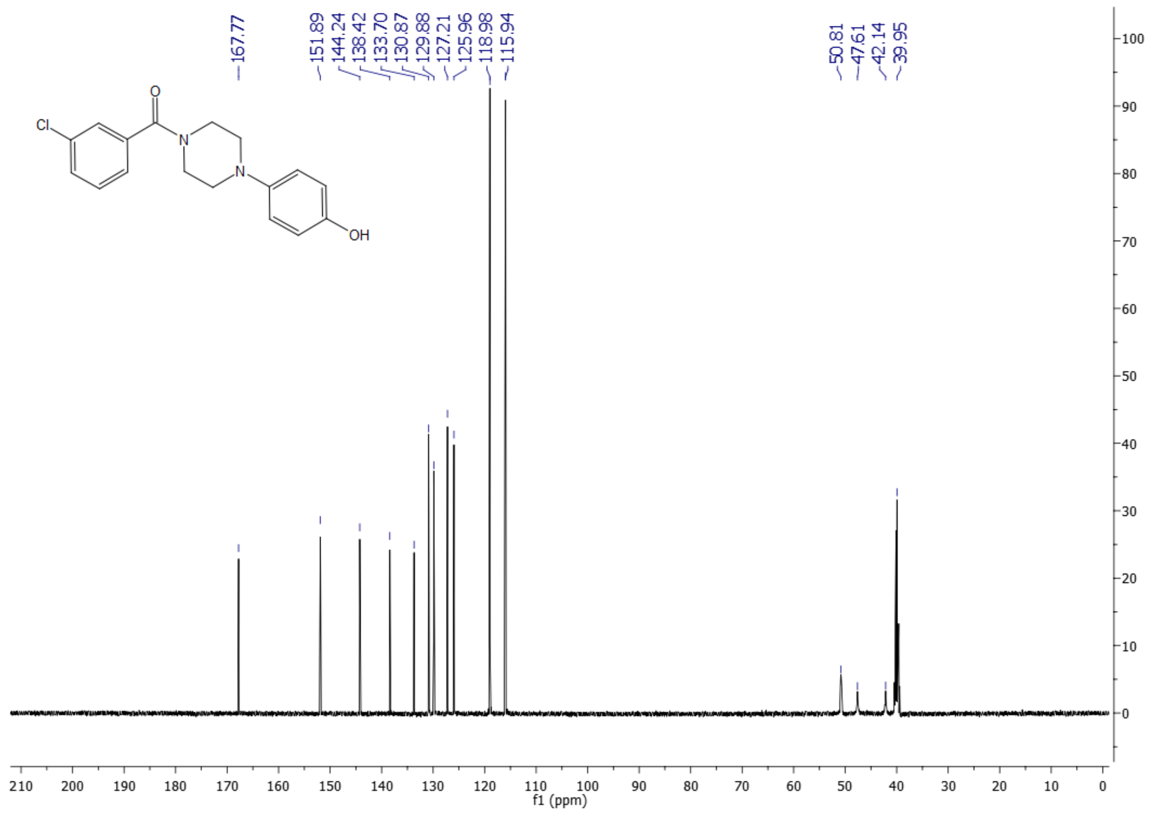

Figure S16: ^{13}C-NMR (DMSO-d$_6$) spectrum of (4-(4-hydroxyphenyl)piperazin-1-yl)(3-chlorophenyl)methanone **(8)**

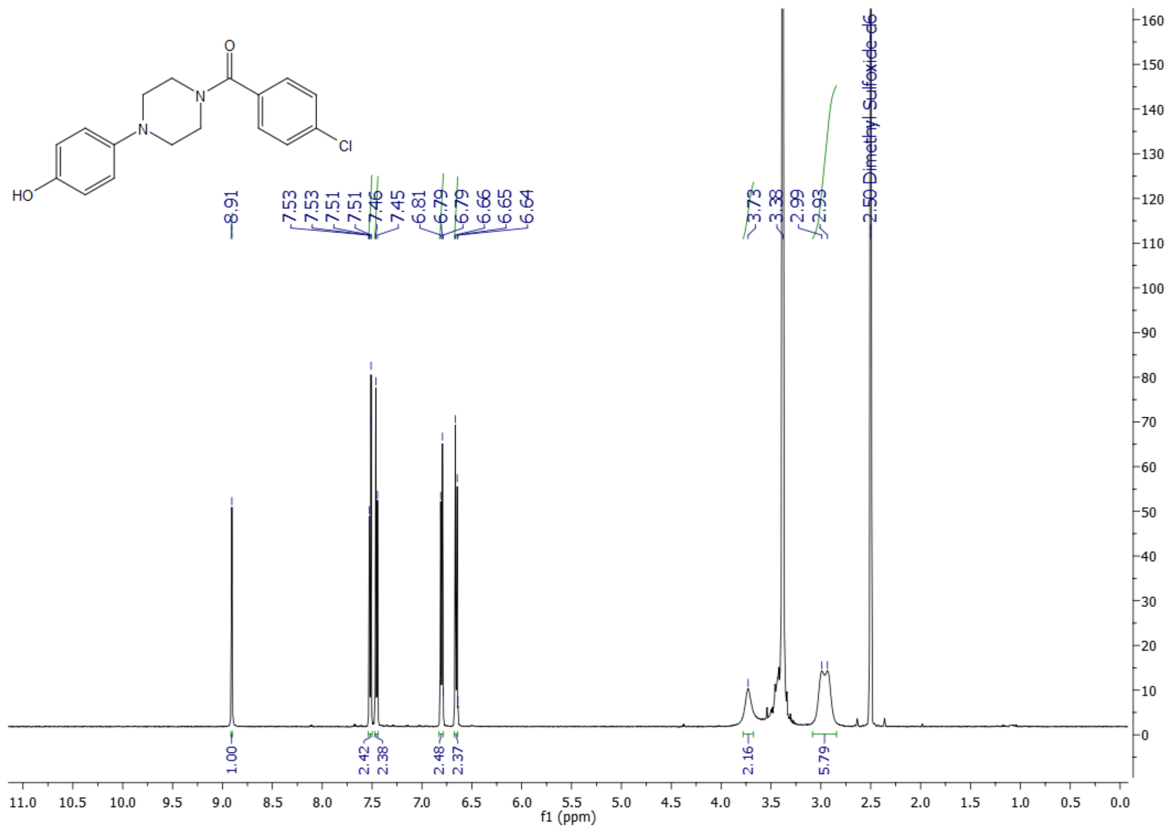

Figure S17: ^{1}H-NMR (DMSO-d_6) spectrum of (4-(4-hydroxyphenyl)piperazin-1-yl)(4-chlorophenyl)methanone **(9)**

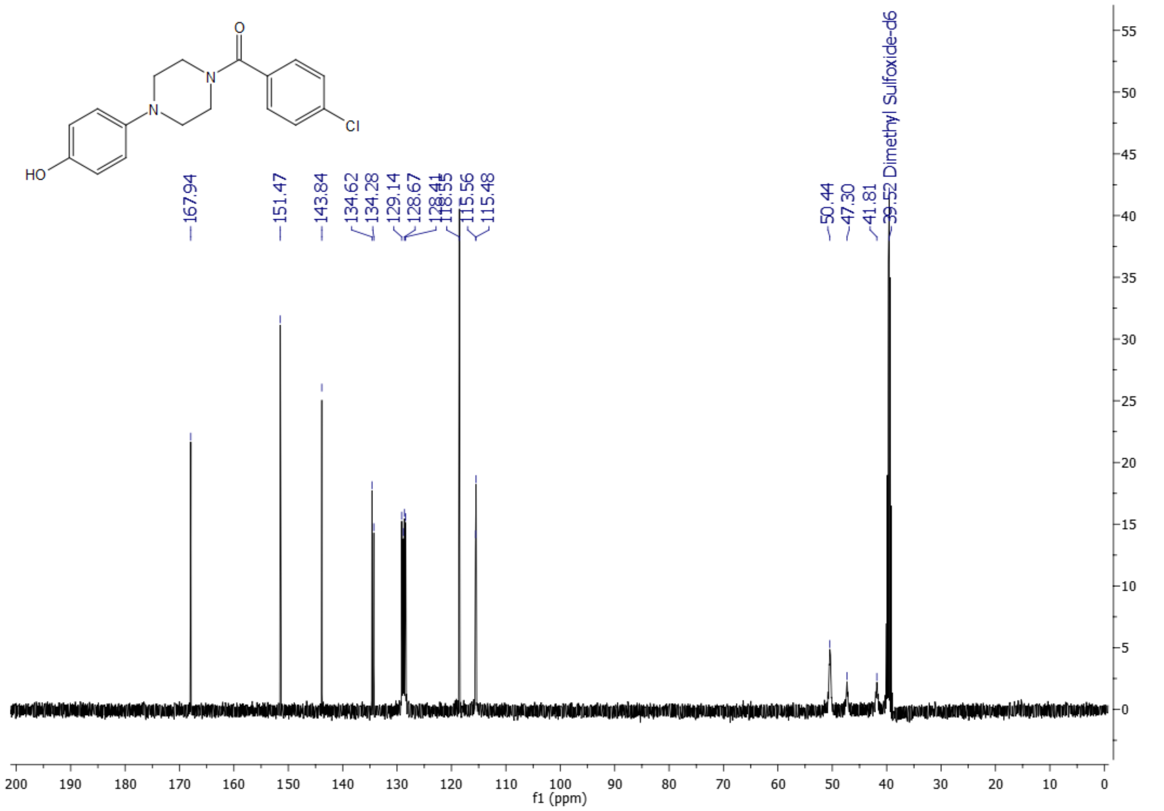

Figure S18: ^{13}C-NMR (DMSO-d_6) spectrum of (4-(4-hydroxyphenyl)piperazin-1-yl)(4-chlorophenyl)methanone **(9)**

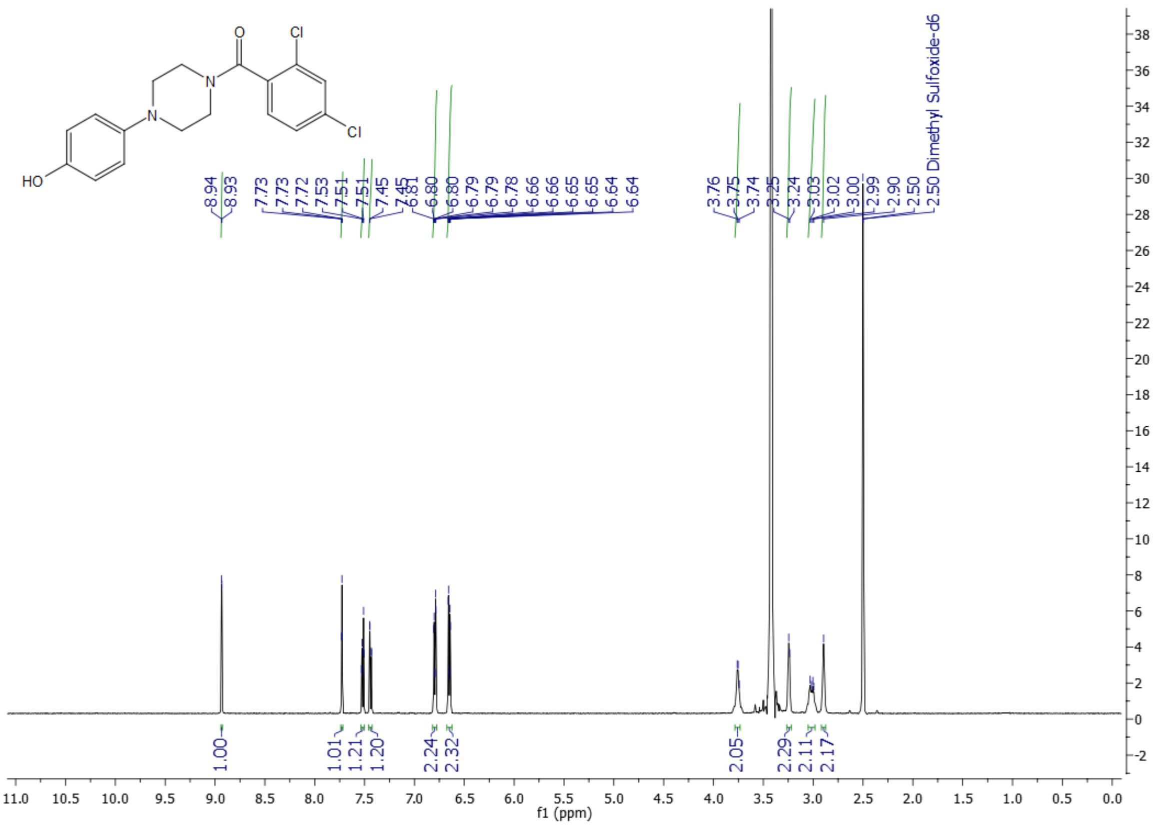

Figure S19: ^{1}H-NMR (DMSO-d$_6$) spectrum of (4-(4-hydroxyphenyl)piperazin-1-yl)(2,4-dichlorophenyl)methanone **(10)**

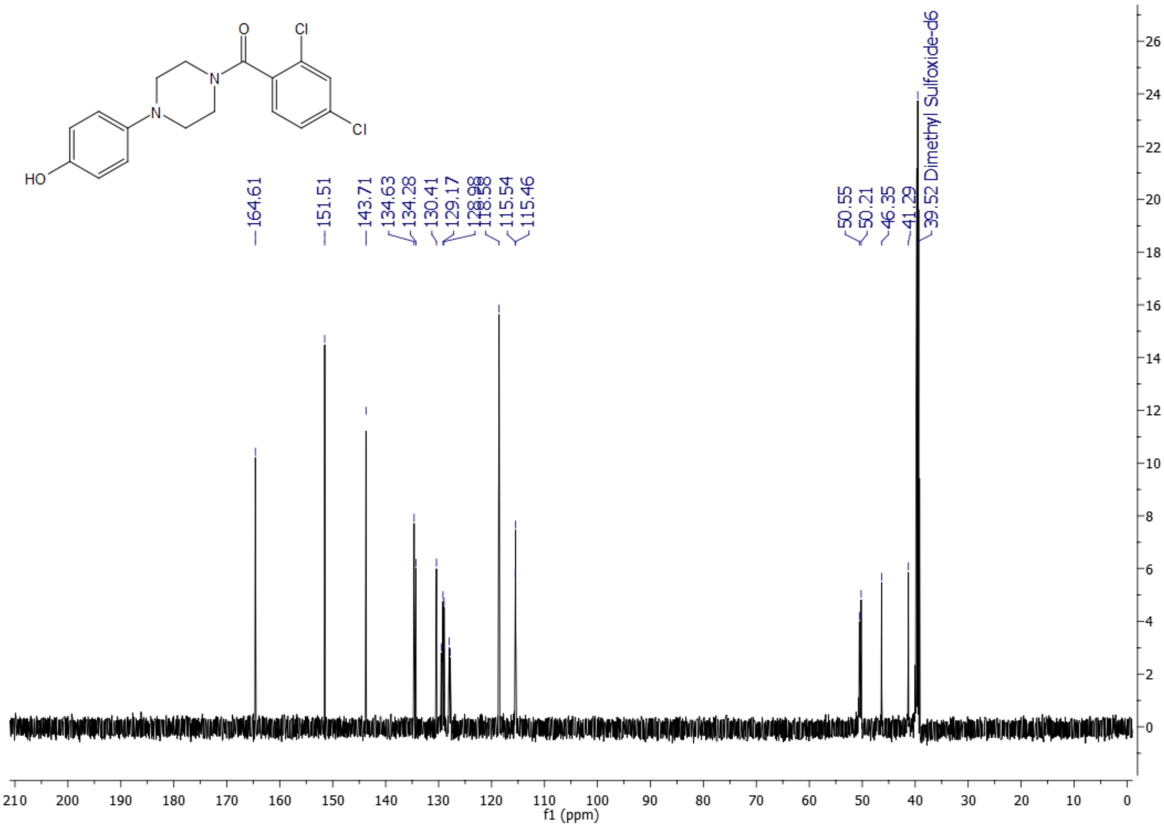

Figure S20: ^{13}C-NMR (DMSO-d$_6$) spectrum of (4-(4-hydroxyphenyl)piperazin-1-yl)(2,4-dichlorophenyl)methanone **(10)**

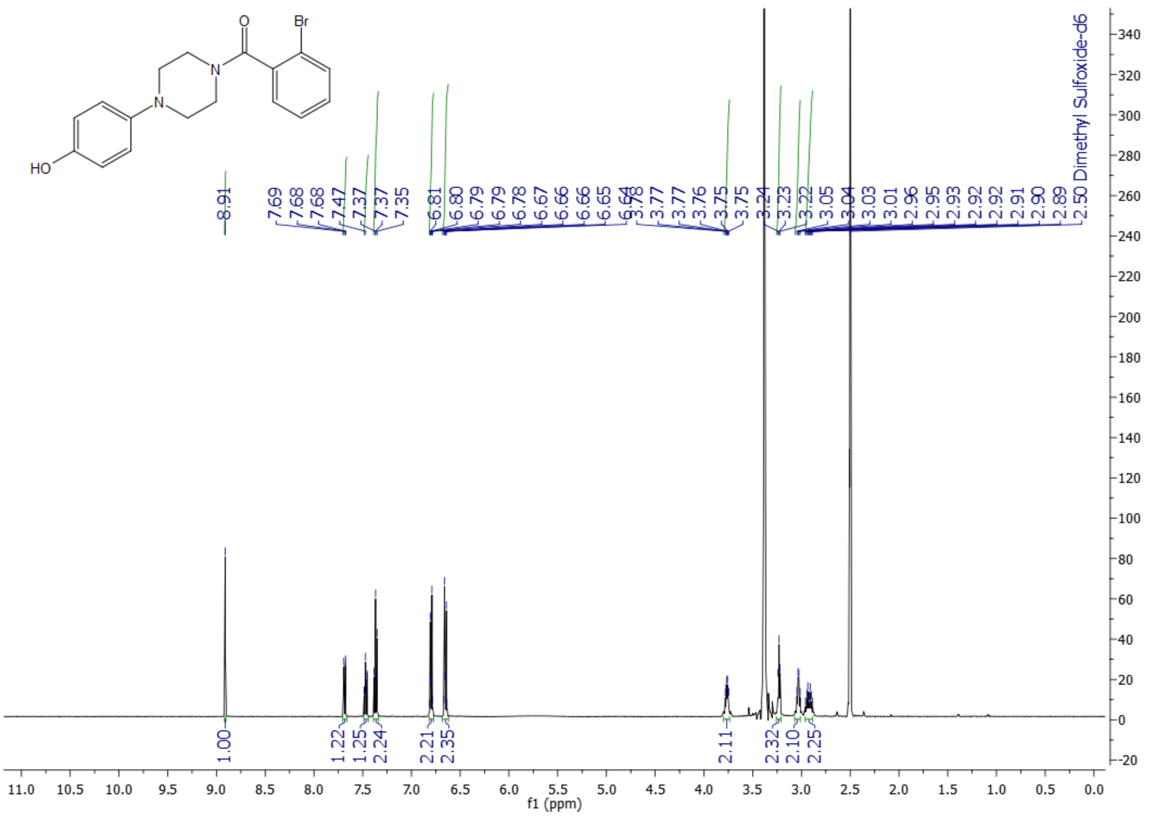

Figure S21: ^{1}H-NMR (DMSO-d$_6$) spectrum of (4-(4-hydroxyphenyl)piperazin-1-yl)(2-bromophenyl)methanone **(11)**

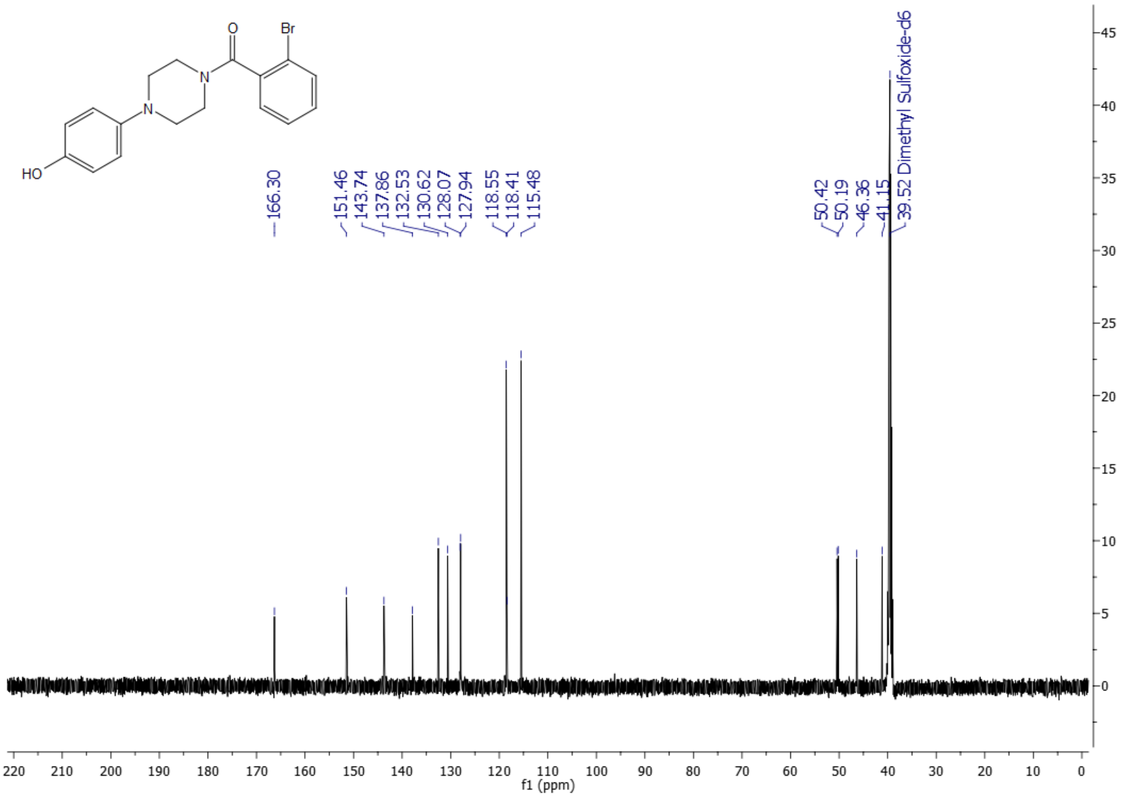

Figure S22: ^{13}C-NMR (DMSO-d$_6$) spectrum of (4-(4-hydroxyphenyl)piperazin-1-yl)(2-bromophenyl)methanone **(11)**

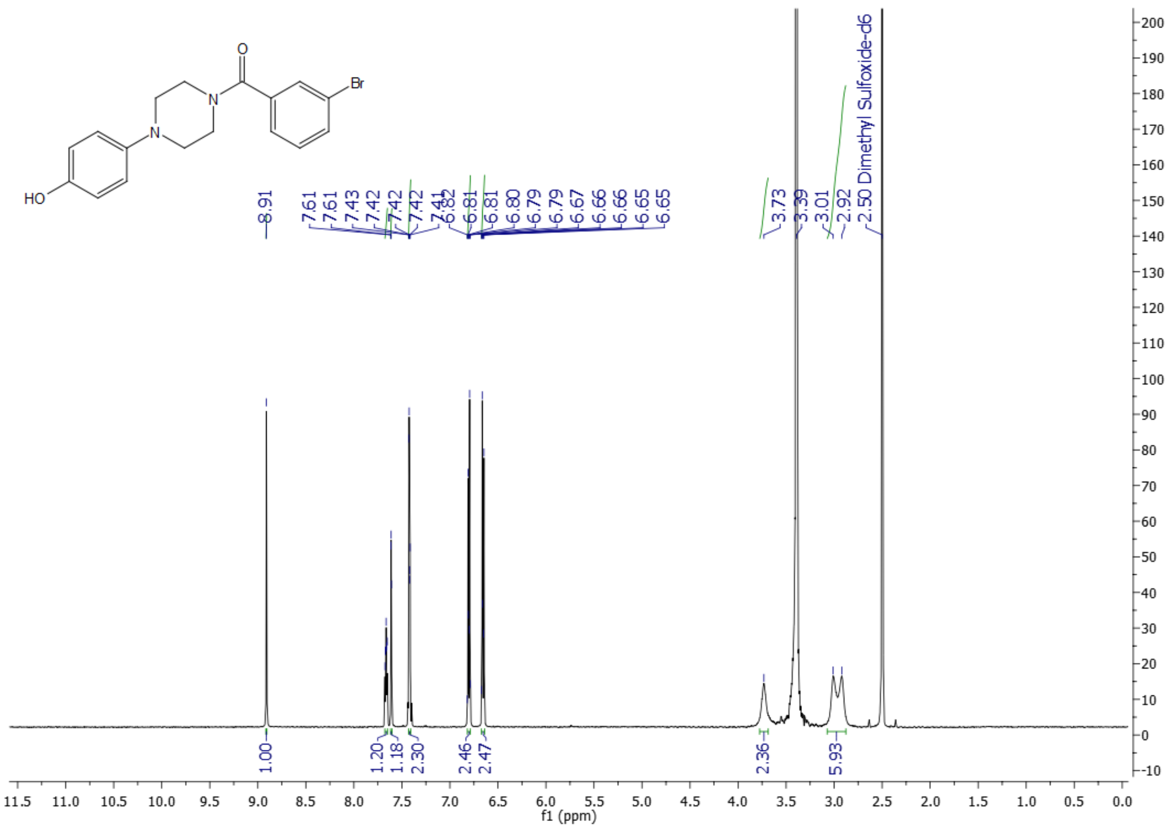

Figure S23: ^{1}H-NMR (DMSO-d_6) spectrum of (4-(4-hydroxyphenyl)piperazin-1-yl)(3-bromophenyl)methanone **(12)**

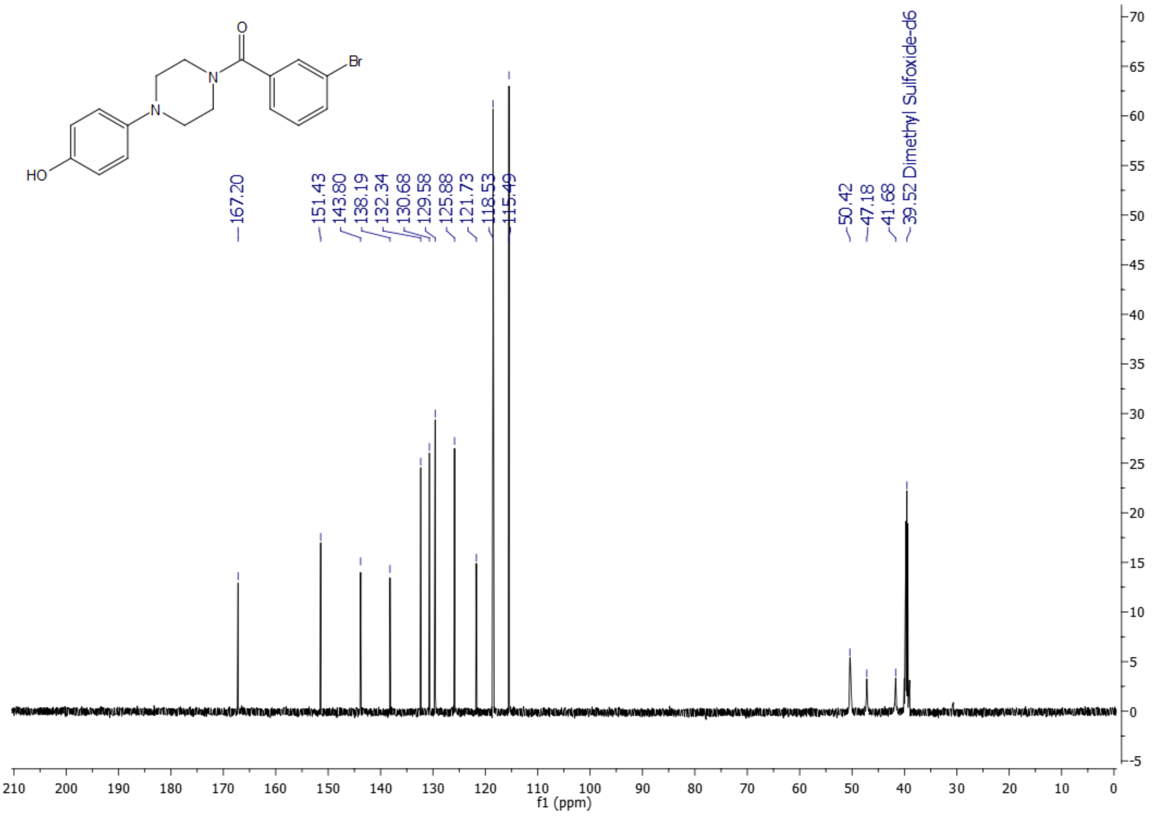

Figure S24: ^{13}C-NMR (DMSO-d_6) spectrum of (4-(4-hydroxyphenyl)piperazin-1-yl)(3-bromophenyl)methanone **(12)**

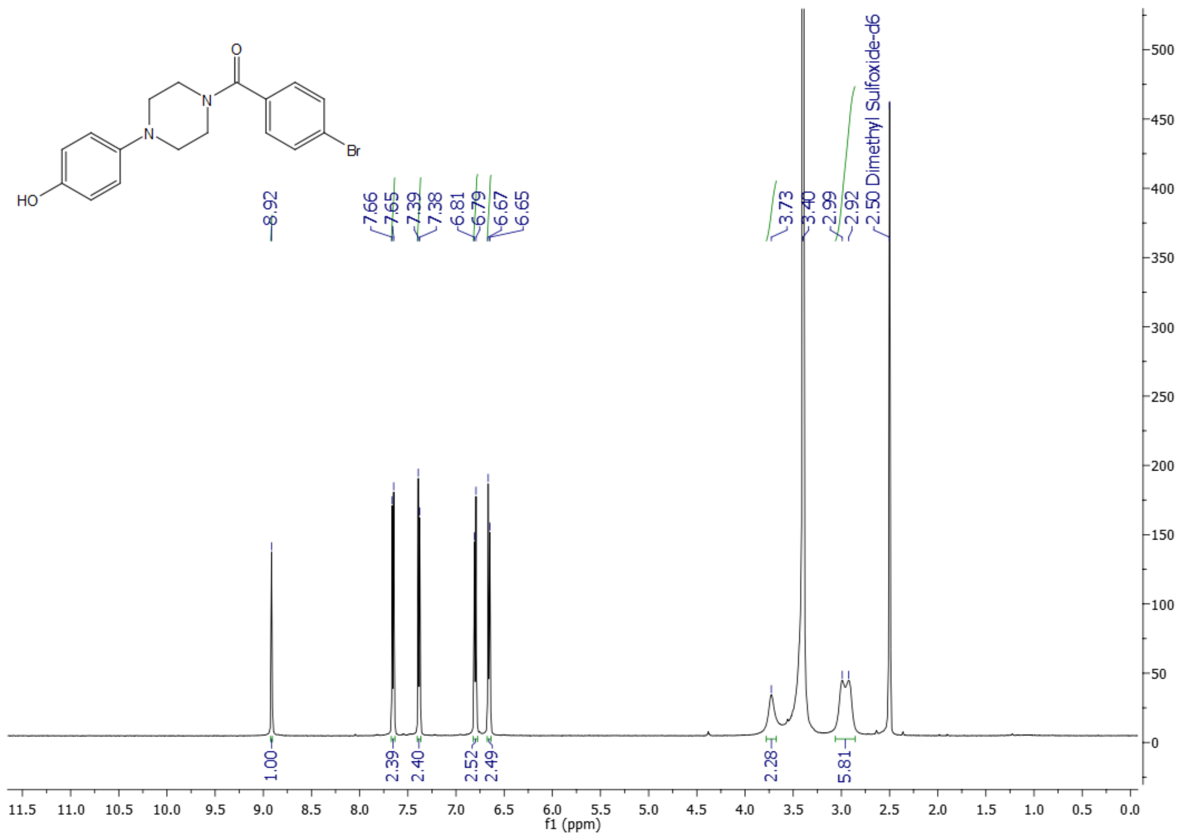

Figure S25: ^{1}H-NMR (DMSO-d_6) spectrum of (4-(4-hydroxyphenyl)piperazin-1-yl)(4-bromophenyl)methanone **(13)**

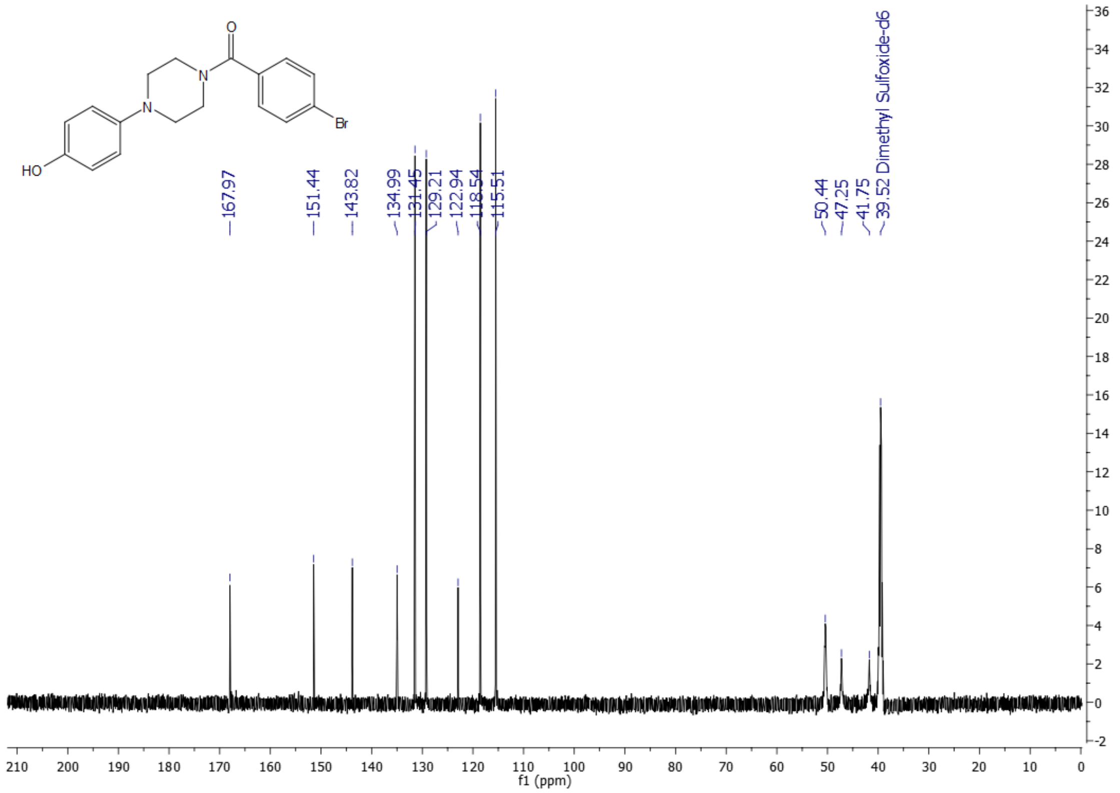

Figure S26: ^{13}C-NMR (DMSO-d_6) spectrum of (4-(4-hydroxyphenyl)piperazin-1-yl)(4-bromophenyl)methanone **(13)**

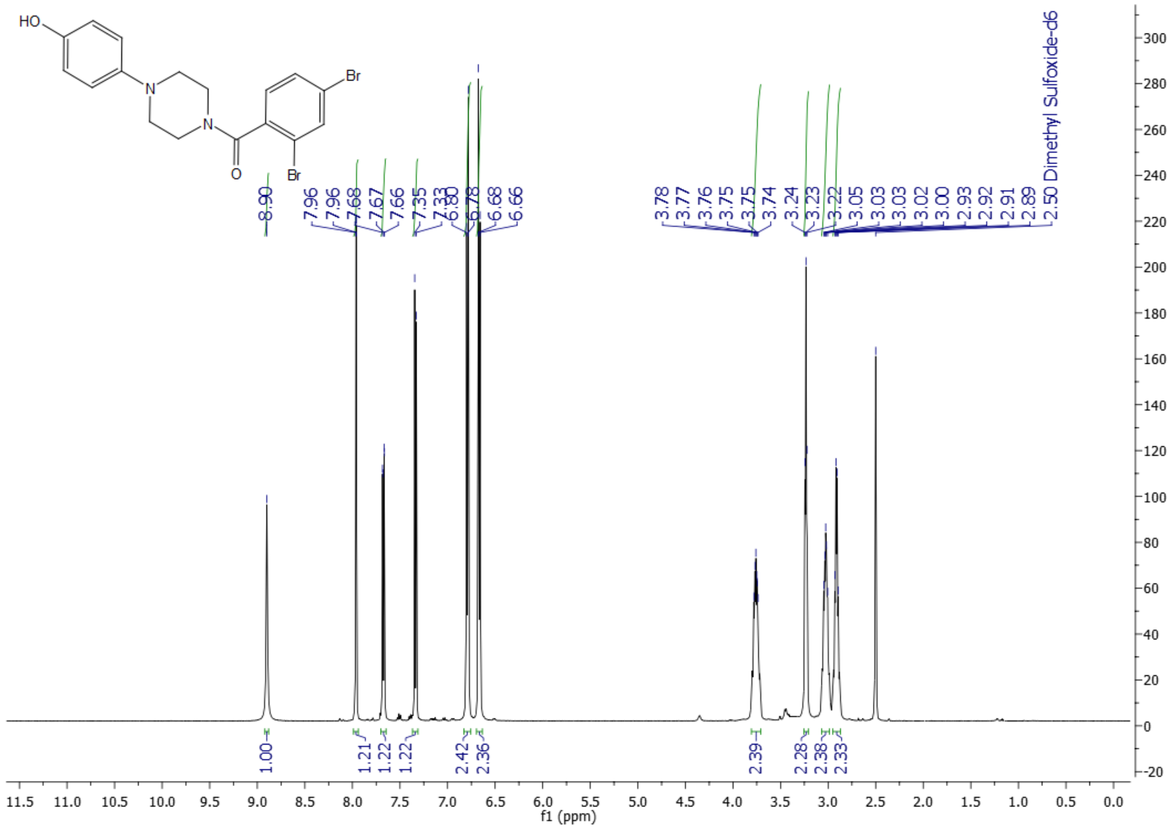

Figure S27: ^{1}H-NMR (DMSO-d$_6$) spectrum of (4-(4-hydroxyphenyl)piperazin-1-yl)(2,4-dibromophenyl)methanone **(26)**

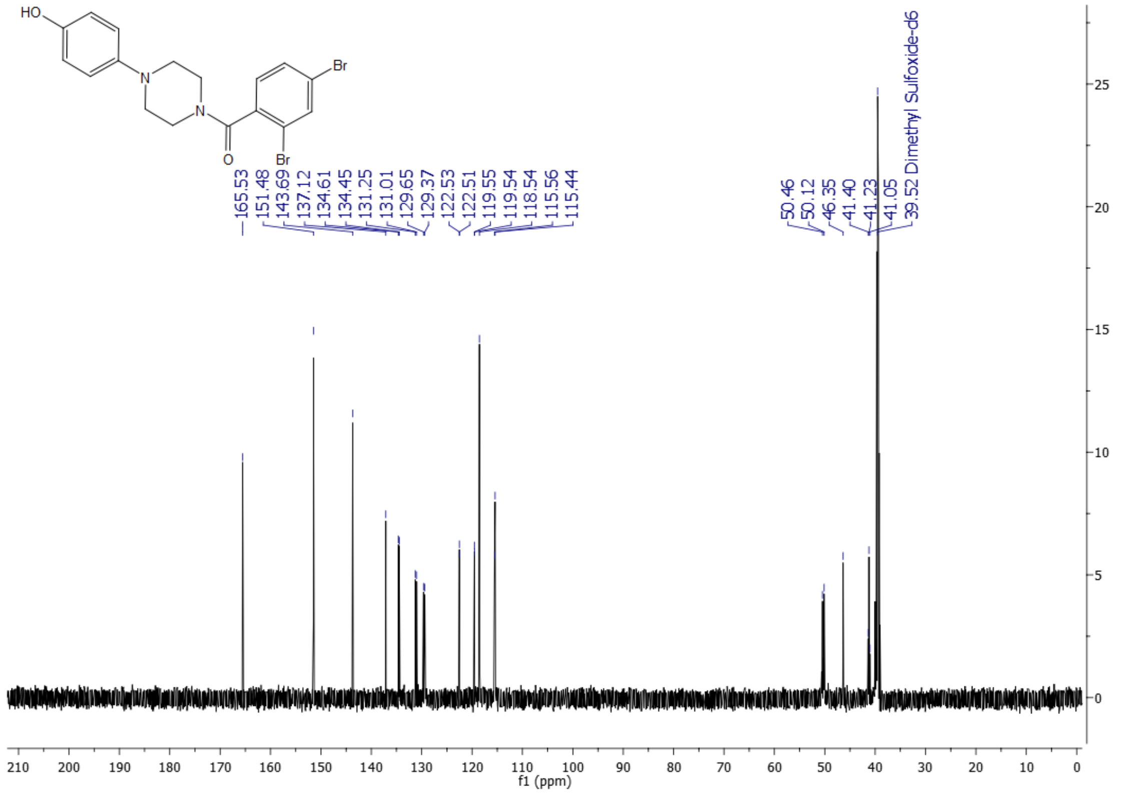

Figure S28: ^{13}C-NMR (DMSO-d$_6$) spectrum of (4-(4-hydroxyphenyl)piperazin-1-yl)(2,4-dibromophenyl)methanone **(26)**

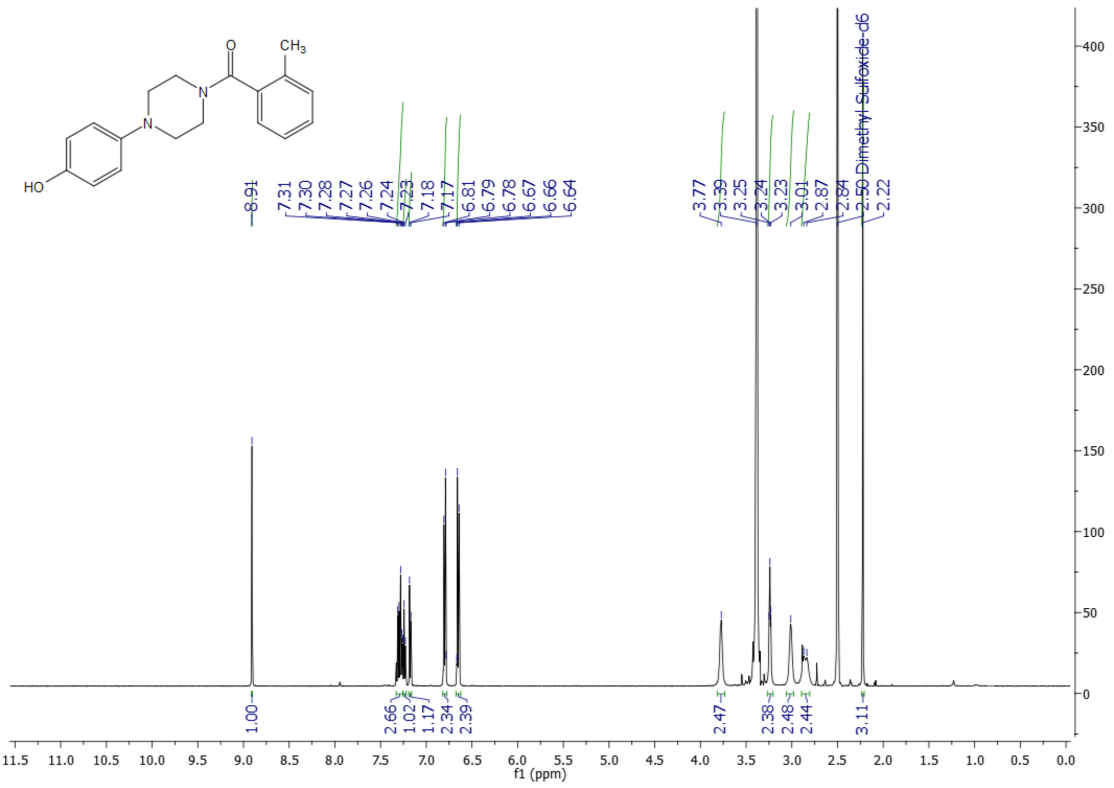

Figure S29: ^{1}H-NMR (DMSO-d$_6$) spectrum of [4-(4-hydroxyphenyl)piperazin-1-yl](2-methylphenyl)methanone **(14)**

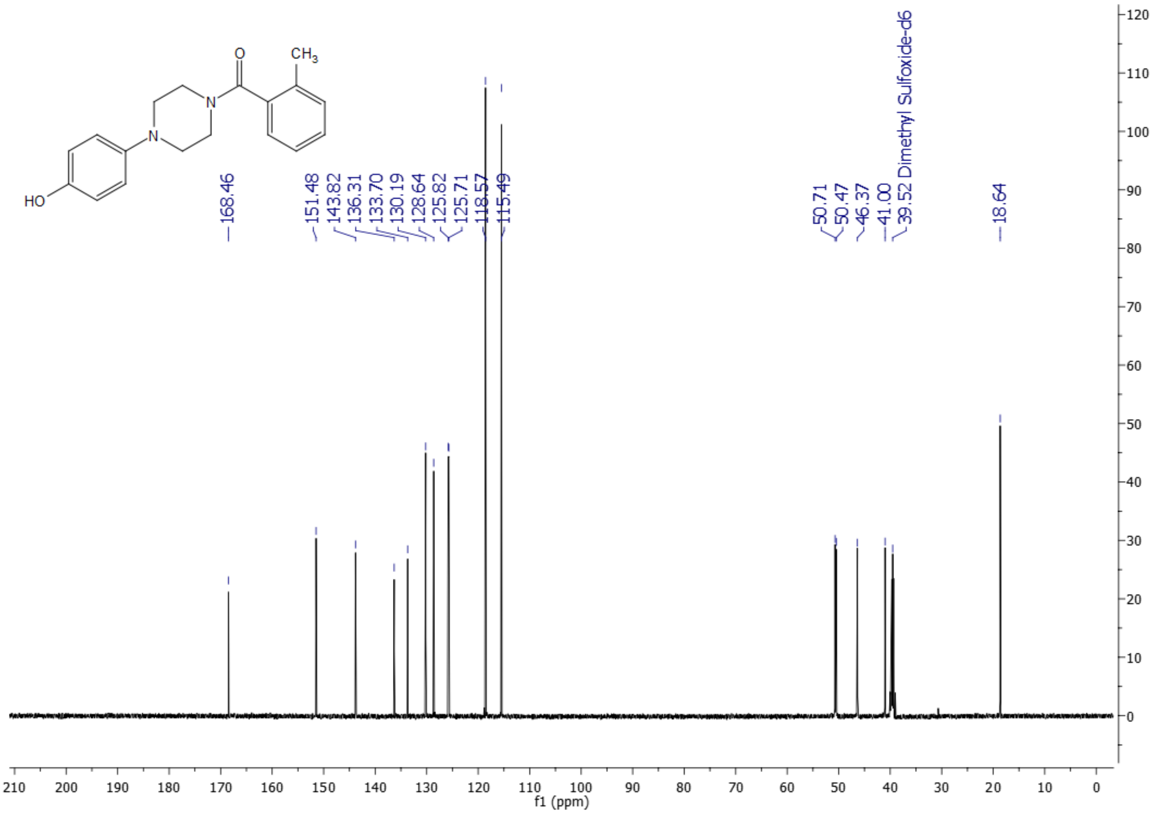

Figure S30: ^{13}C-NMR (DMSO-d$_6$) spectrum of [4-(4-hydroxyphenyl)piperazin-1-yl](2-methylphenyl)methanone **(14)**

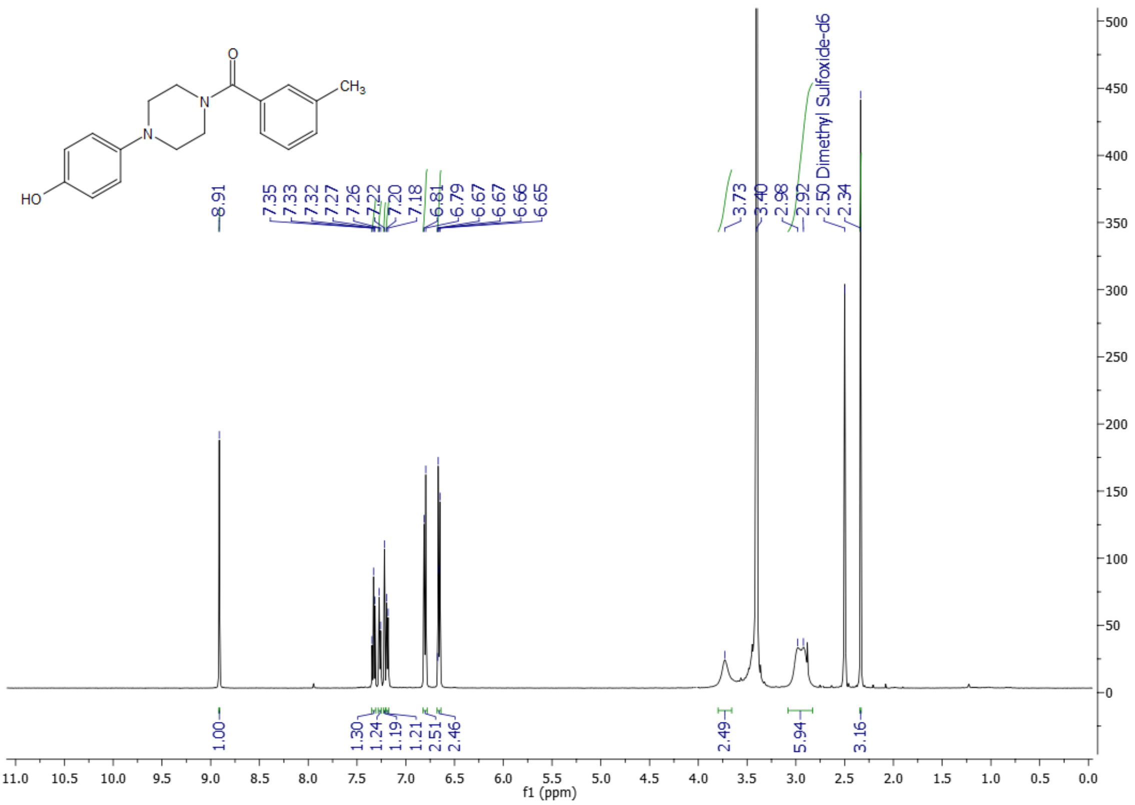

Figure S31: ^{1}H-NMR (DMSO-d_6) spectrum of [4-(4-hydroxyphenyl)piperazin-1-yl](3-methylphenyl)methanone (**15**)

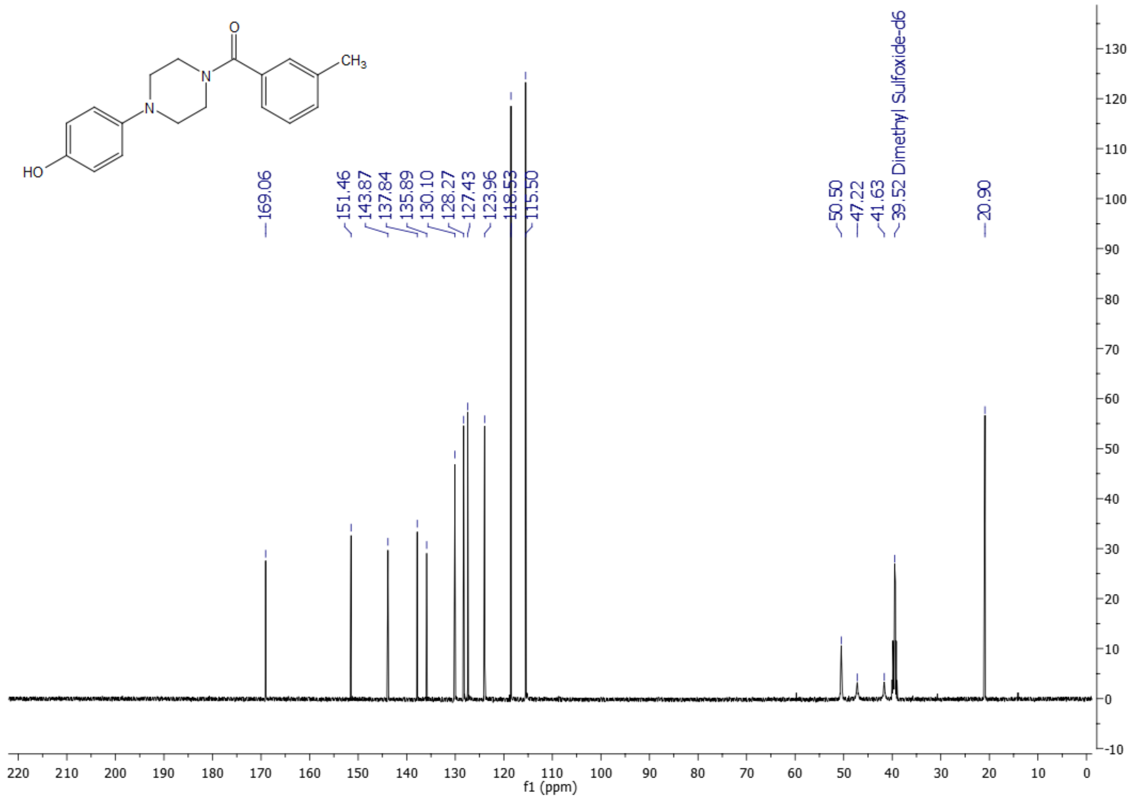

Figure S32: ^{13}C-NMR (DMSO-d_6) spectrum of [4-(4-hydroxyphenyl)piperazin-1-yl](3-methylphenyl)methanone (**15**)

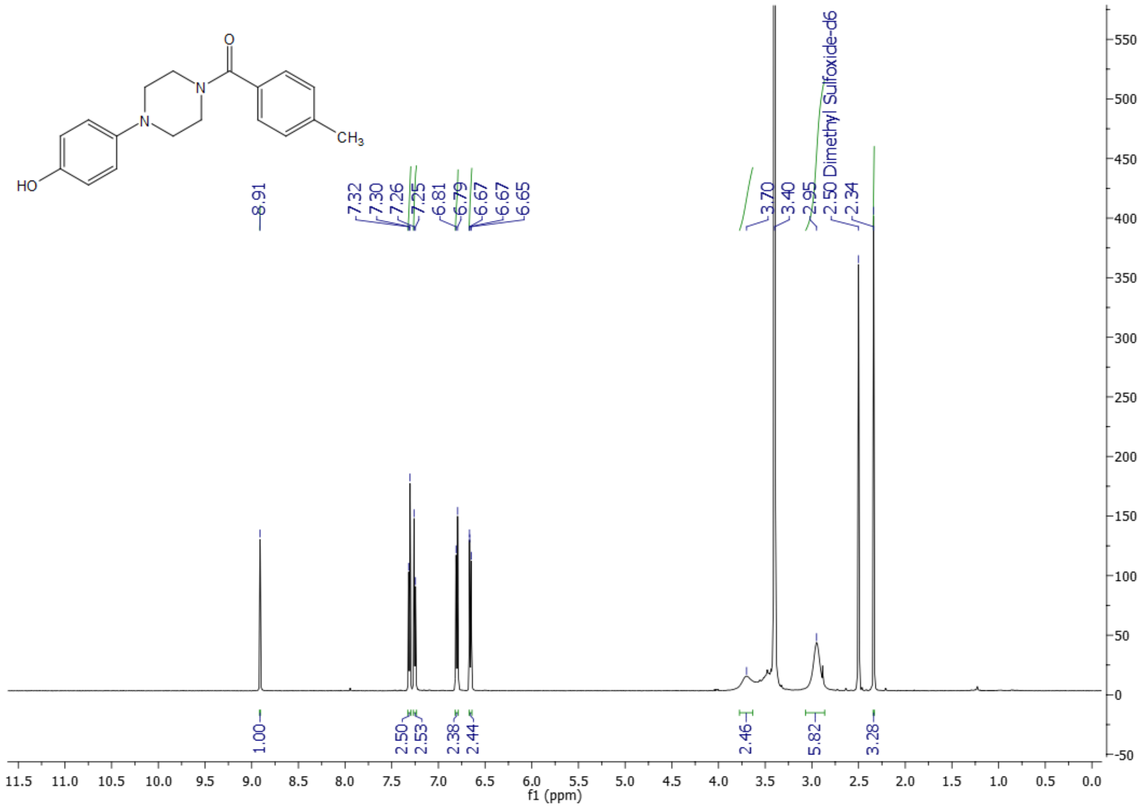

Figure S33: ^{1}H-NMR (DMSO-d$_6$) spectrum of [4-(4-hydroxyphenyl)piperazin-1-yl](4-methylphenyl)methanone **(16)**

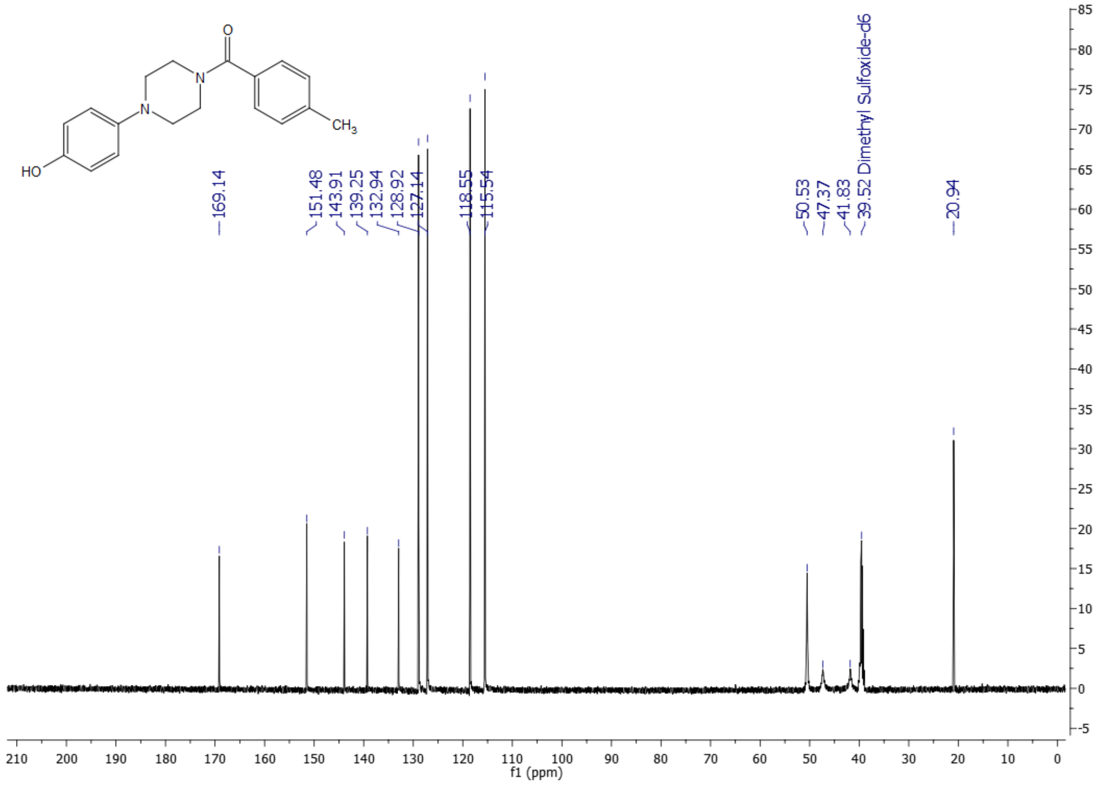

Figure S34: ^{13}C-NMR (DMSO-d$_6$) spectrum of [4-(4-hydroxyphenyl)piperazin-1-yl](4-methylphenyl)methanone **(16)**

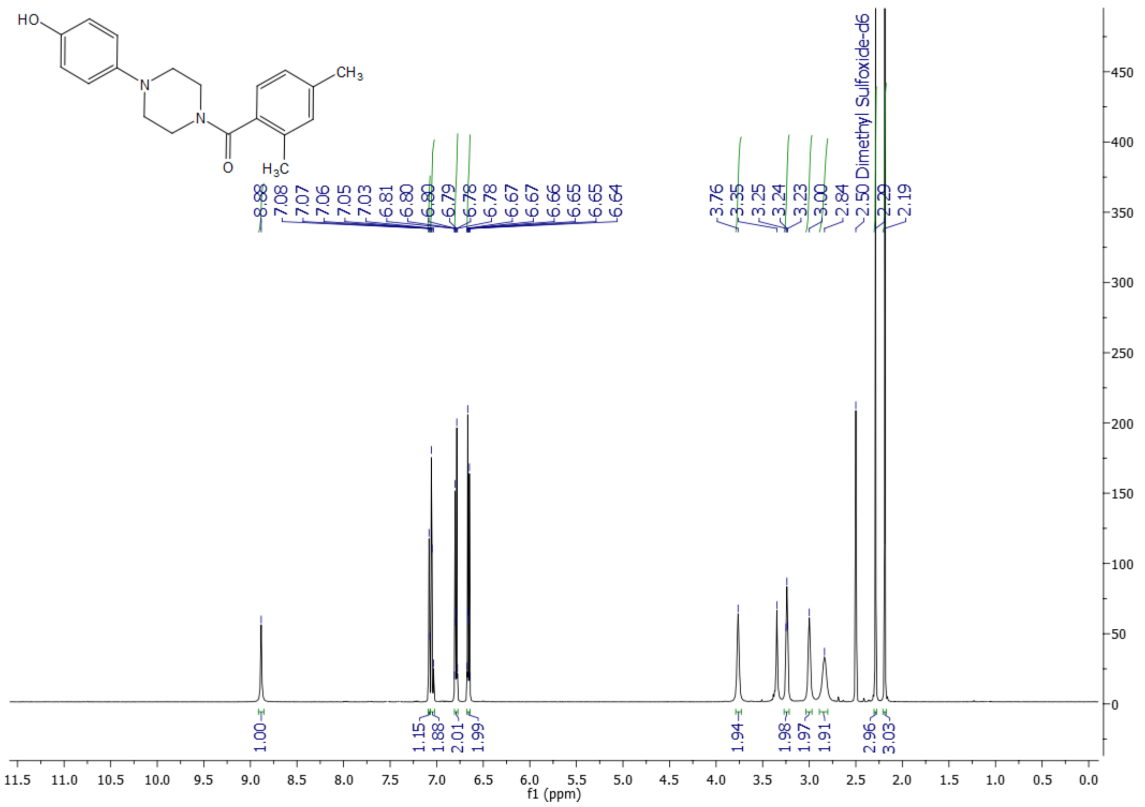

Figure S35: ^{1}H-NMR (DMSO-d$_6$) spectrum of [4-(4-hydroxyphenyl)piperazin-1-yl](2,4-dimethylphenyl)methanone **(27)**

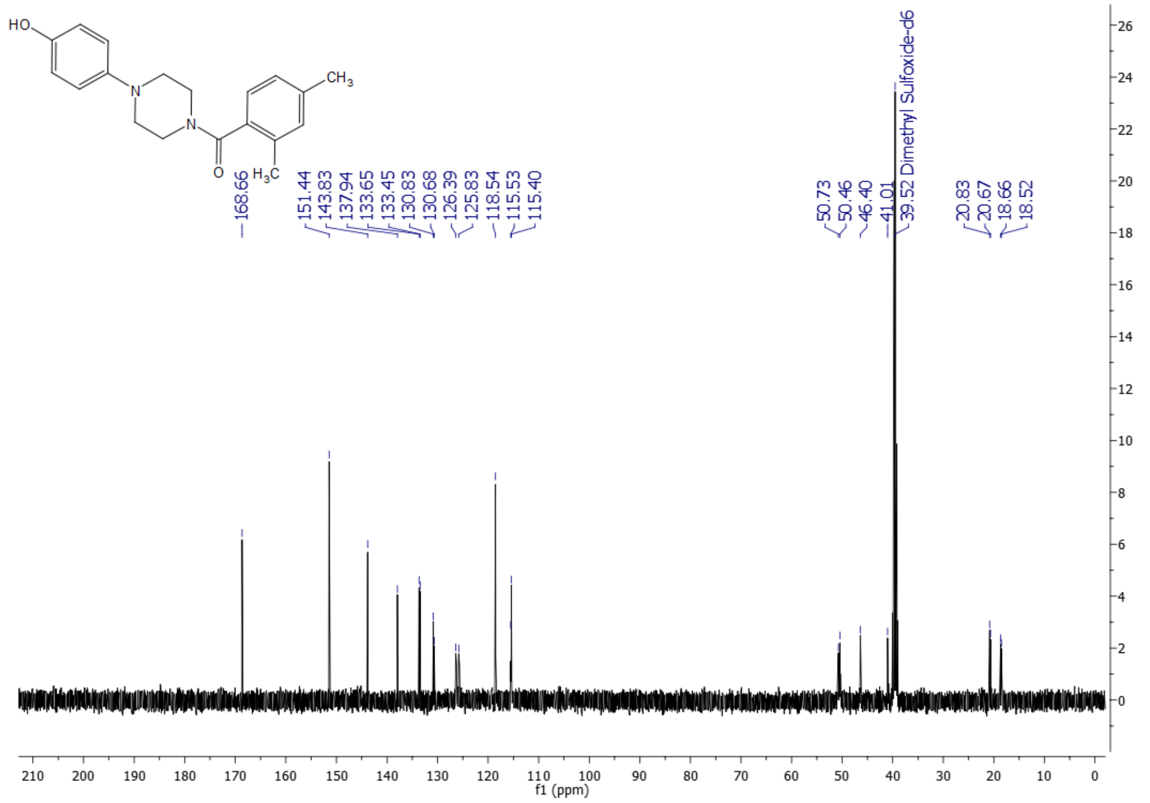

Figure S36: ^{13}C-NMR (DMSO-d$_6$) spectrum of 4-(4-hydroxyphenyl)piperazin-1-yl](2,4-dimethylphenyl)methanone **(27)**

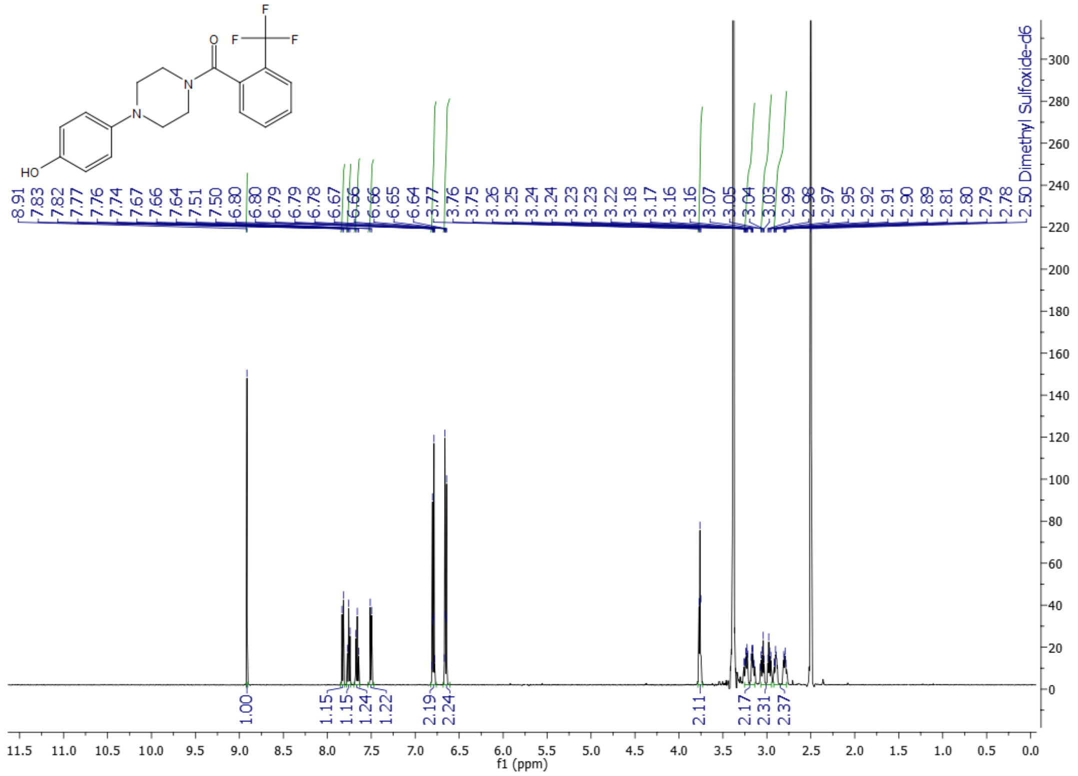

Figure S37: ^{1}H-NMR (DMSO-d_6) spectrum of (4-(4-hydroxyphenyl)piperazin-1-yl)(2-(trifluoromethyl)phenyl)methanone **(17)**

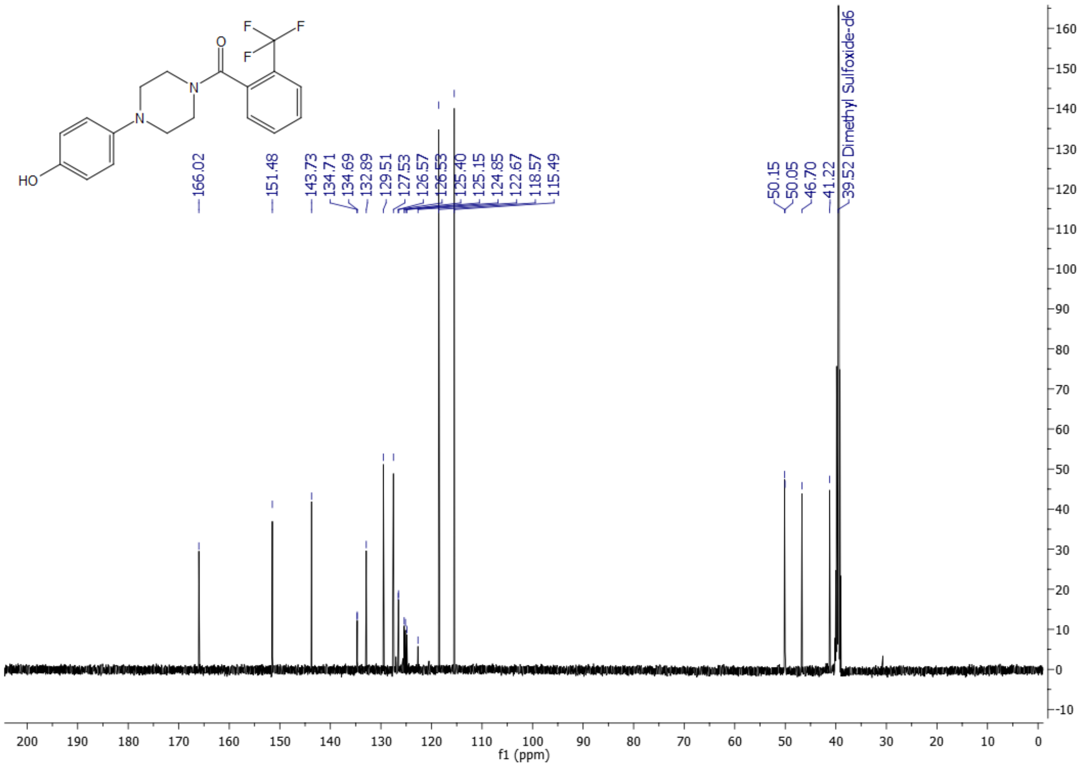

Figure S38: ^{13}C-NMR (DMSO-d_6) spectrum of (4-(4-hydroxyphenyl)piperazin-1-yl)(2-(trifluoromethyl)phenyl)methanone **(17)**

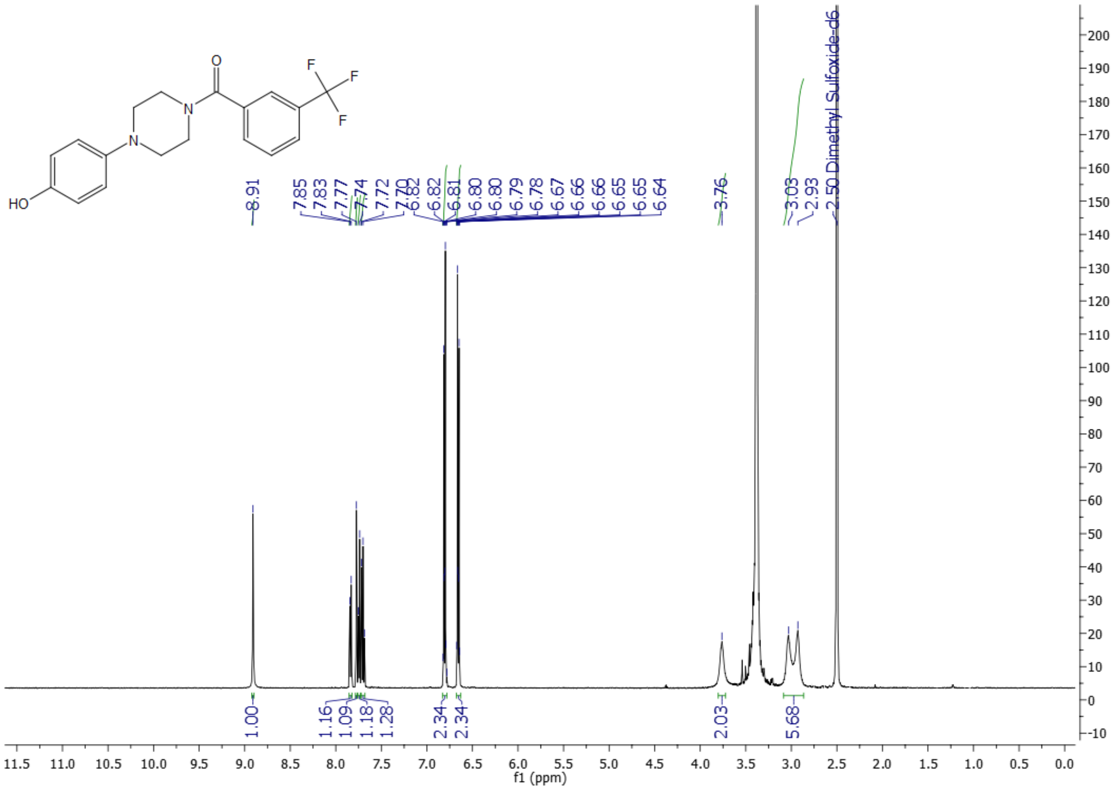

Figure S39: ^{1}H-NMR (DMSO-d$_6$) spectrum of (4-(4-hydroxyphenyl)piperazin-1-yl)(3-(trifluoromethyl)phenyl)methanone **(18)**

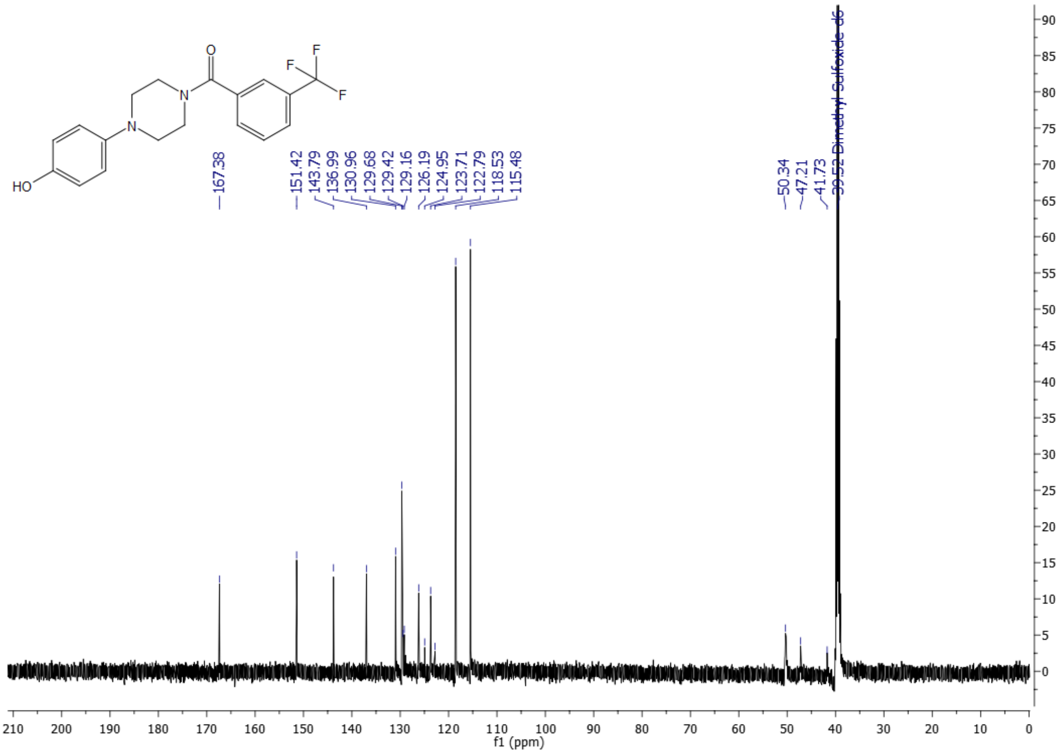

Figure S40: ^{13}C-NMR (DMSO-d$_6$) spectrum of (4-(4-hydroxyphenyl)piperazin-1-yl)(3-(trifluoromethyl)phenyl)methanone **(18)**

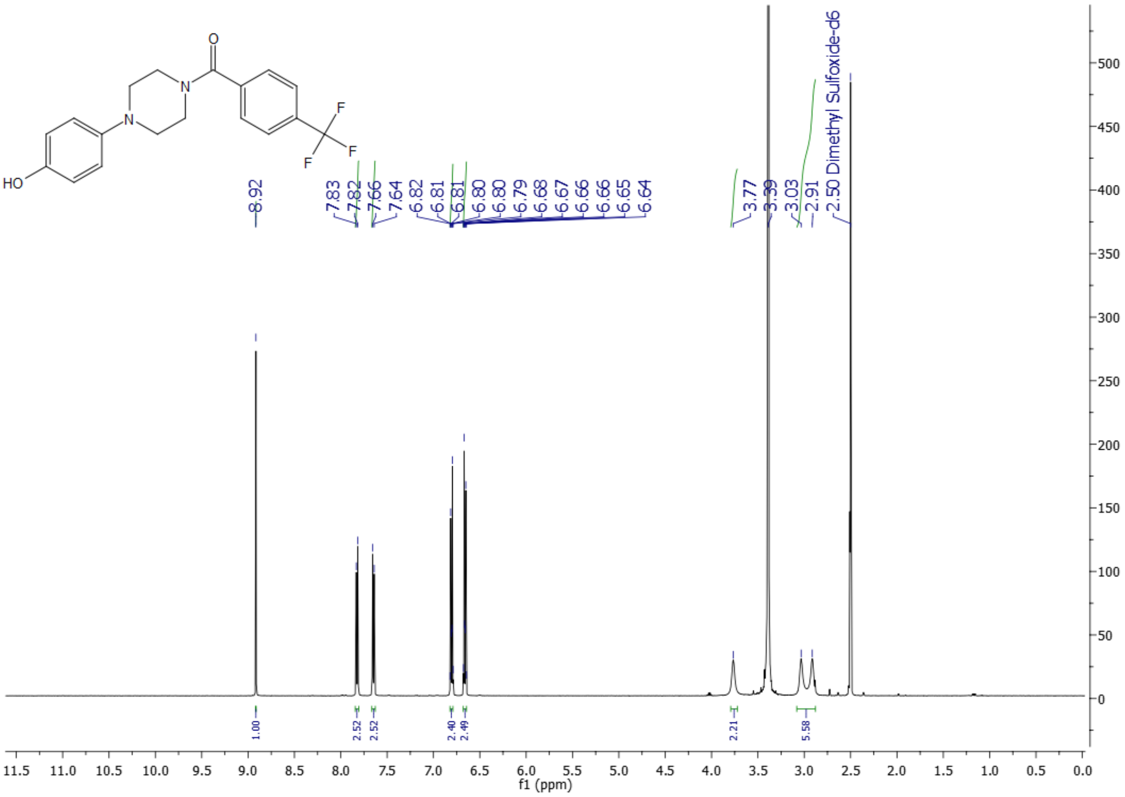

Figure S41: ^{1}H-NMR (DMSO-d_6) spectrum of (4-(4-hydroxyphenyl)piperazin-1-yl)(4-(trifluoromethyl)phenyl)methanone **(19)**

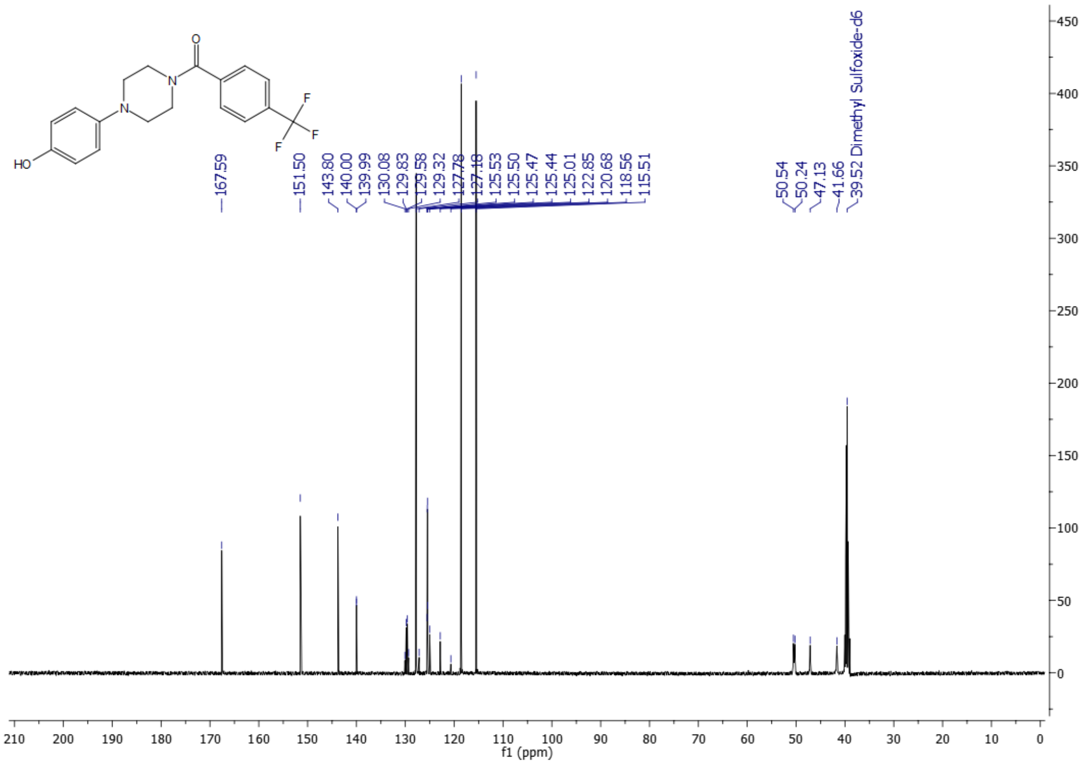

Figure S42: ^{13}C-NMR (DMSO-d_6) spectrum of (4-(4-hydroxyphenyl)piperazin-1-yl)(4-(trifluoromethyl)phenyl)methanone **(19)**

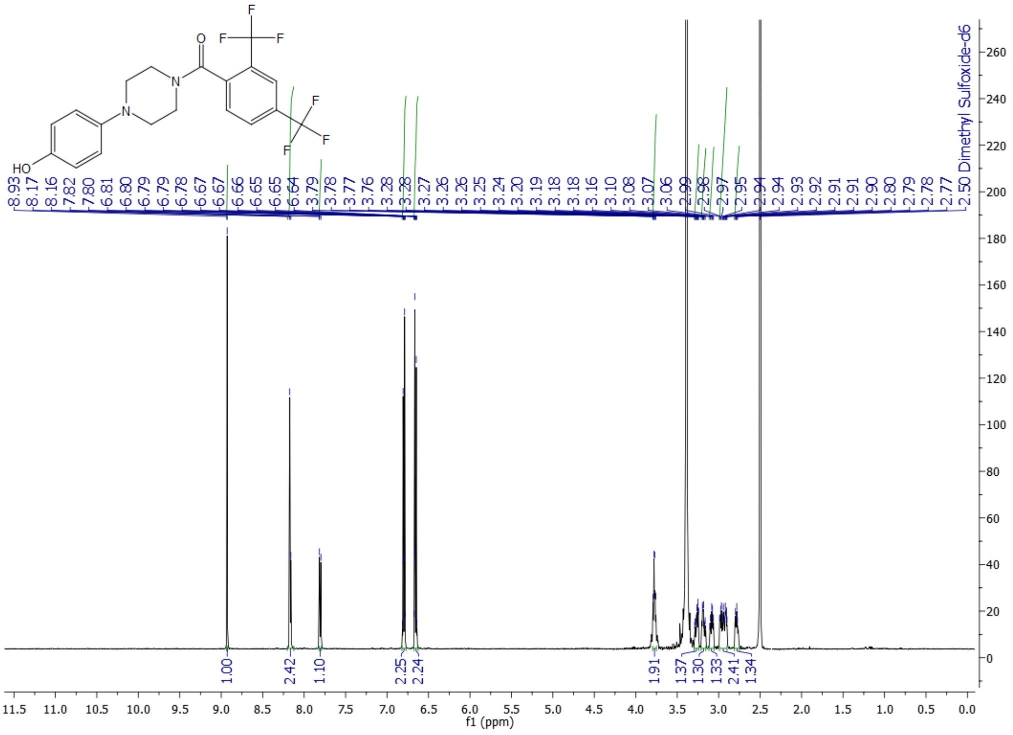

Figure S43: ^{1}H-NMR (DMSO-d_6) spectrum of (4-(4-hydroxyphenyl)piperazin-1-yl)(2,4-bis(trifluoromethyl)phenyl)methanone **(20)**

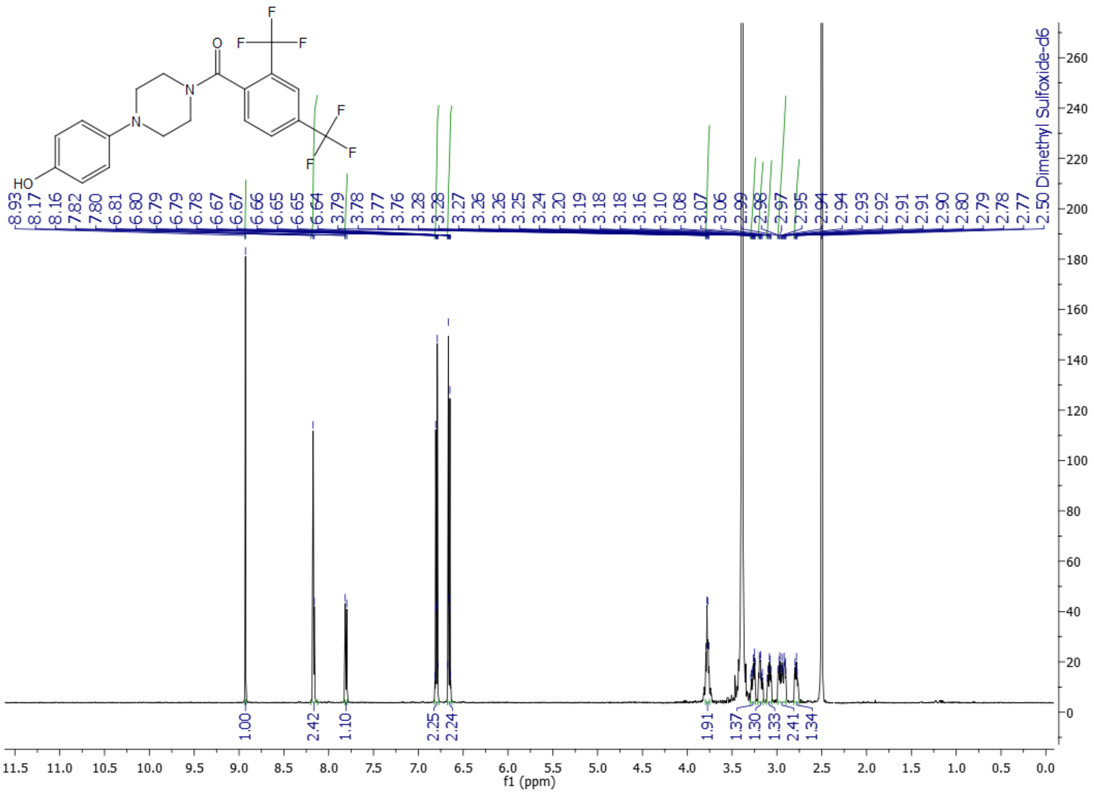

Figure S44: ^{13}C-NMR (DMSO-d_6) spectrum of (4-(4-hydroxyphenyl)piperazin-1-yl)(2,4-bis(trifluoromethyl)phenyl)methanone **(20)**

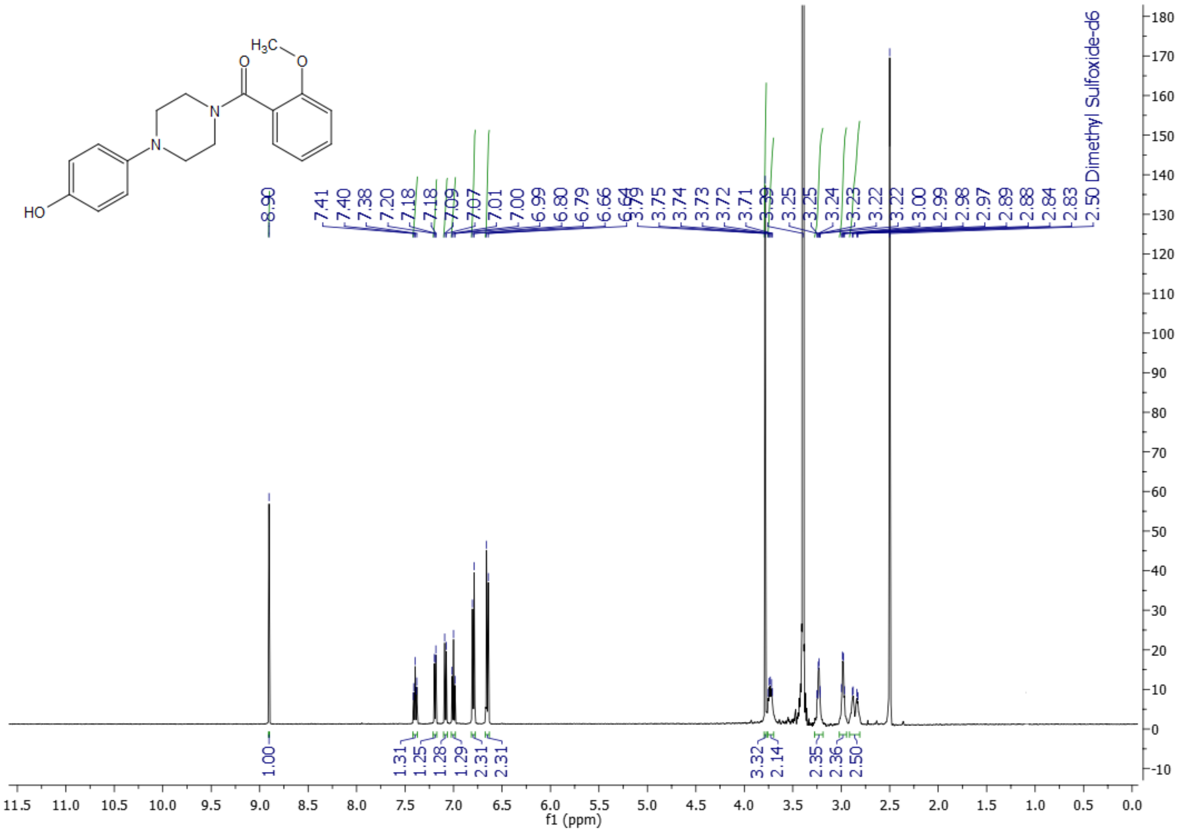

Figure S45: ^{1}H-NMR (DMSO-d_6) spectrum of [4-(4-hydroxyphenyl)piperazin-1-yl](2-methoxyphenyl)methanone **(21)**

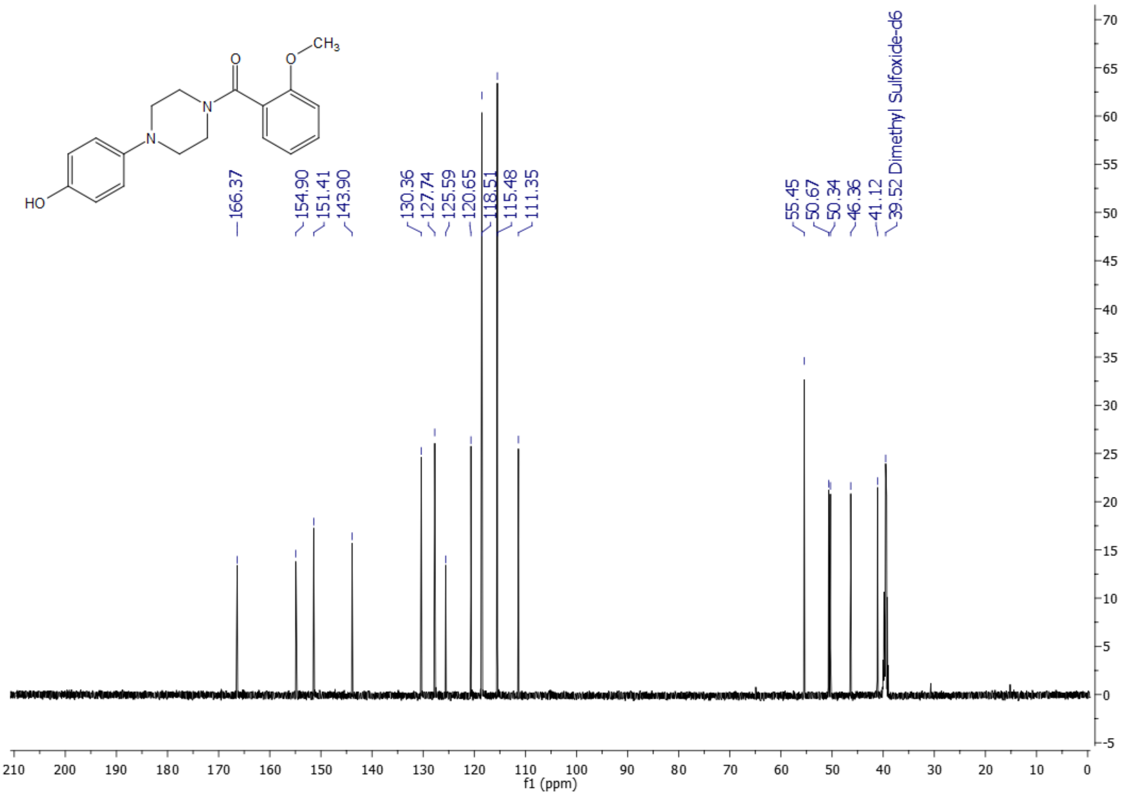

Figure S46: ^{13}C-NMR (DMSO-d_6) spectrum of [4-(4-hydroxyphenyl)piperazin-1-yl](2-methoxyphenyl)methanone **(21)**

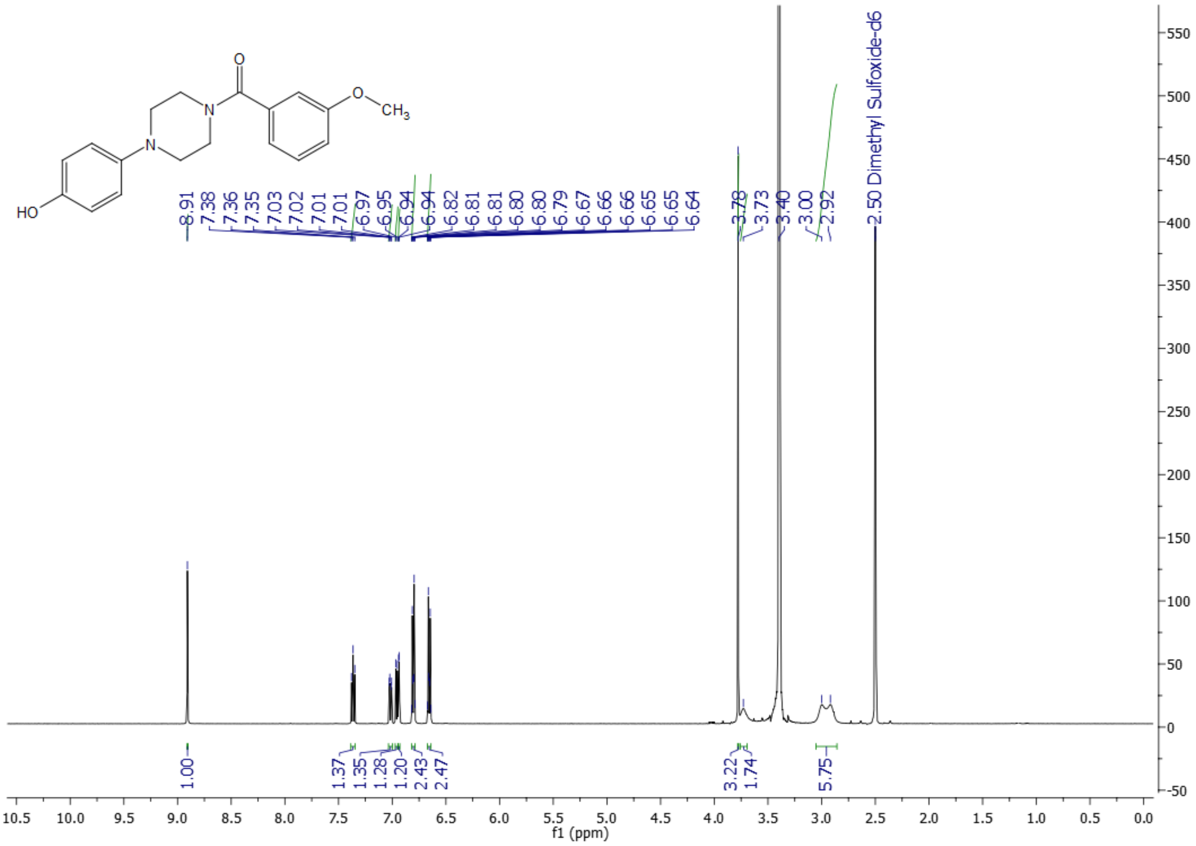

Figure S47: ^{1}H-NMR (DMSO-d$_6$) spectrum of [4-(4-hydroxyphenyl)piperazin-1-yl](3-methoxyphenyl)methanone **(22)**

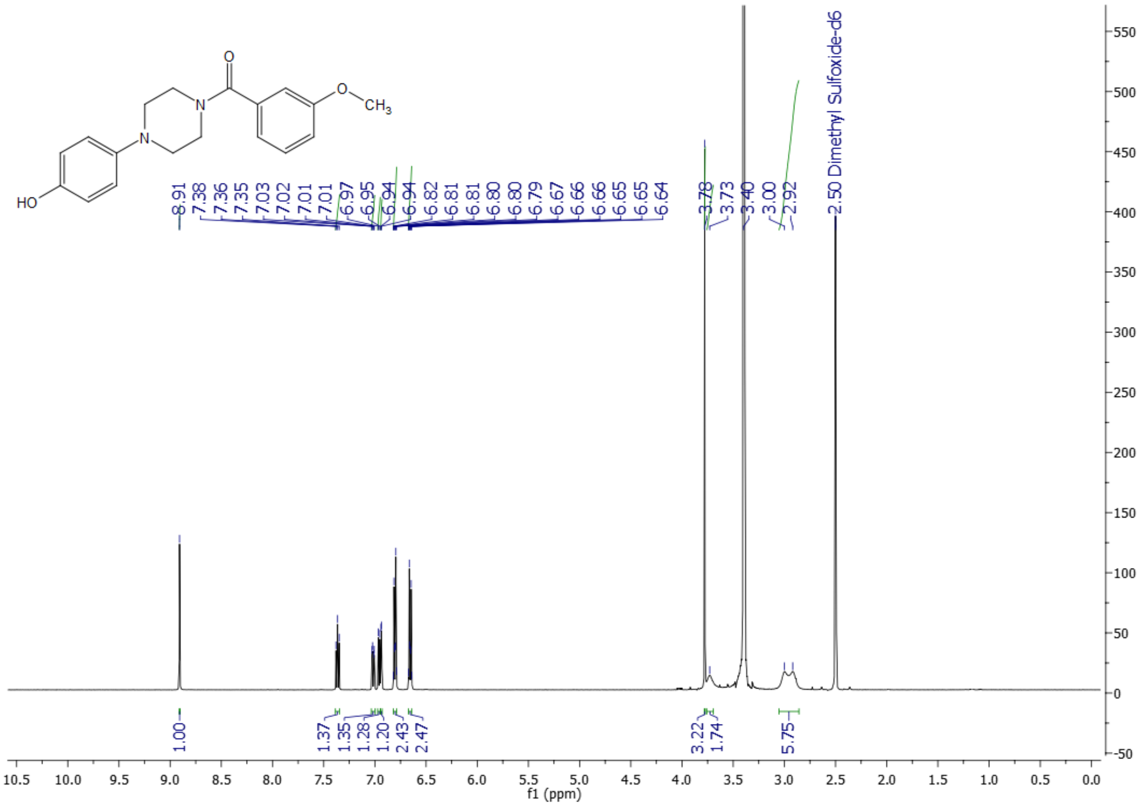

Figure S48: ^{13}C-NMR (DMSO-d$_6$) spectrum of [4-(4-hydroxyphenyl)piperazin-1-yl](3-methoxyphenyl)methanone **(22)**

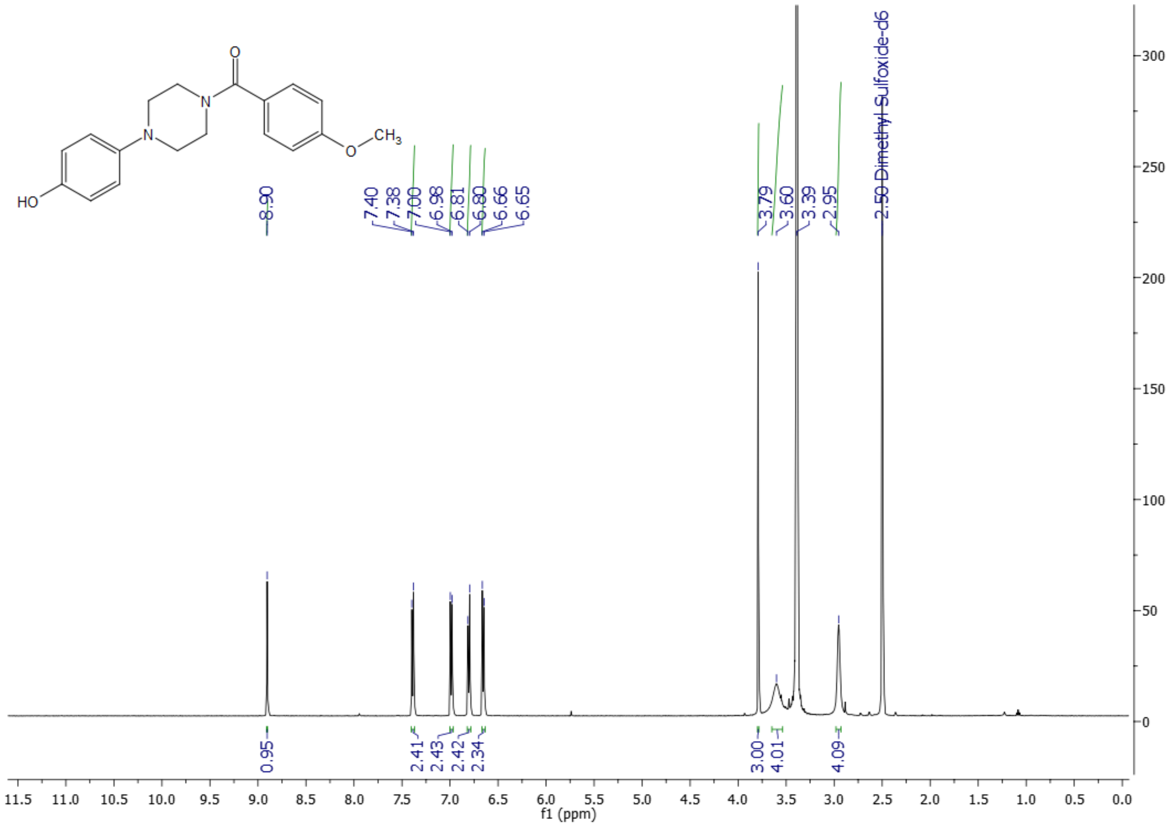

Figure S49: ^{1}H-NMR (DMSO-d_6) spectrum of [4-(4-hydroxyphenyl)piperazin-1-yl](4-methoxyphenyl)methanone **(23)**

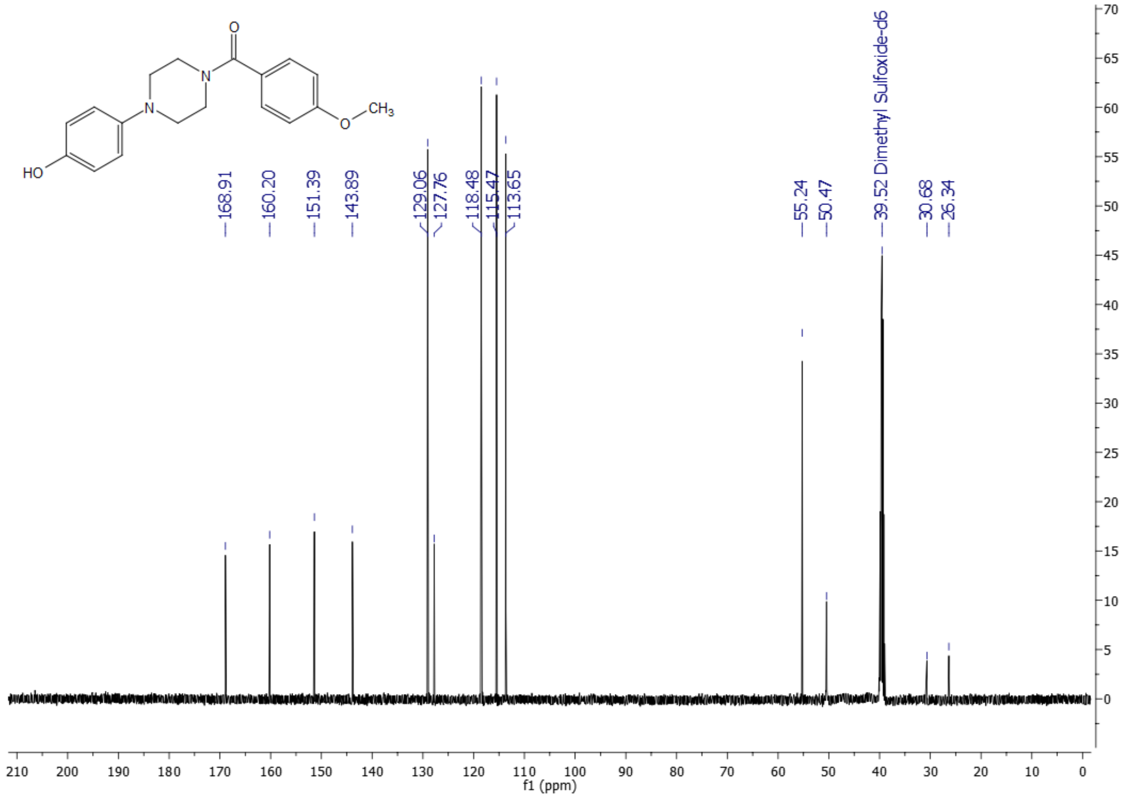

Figure S50: ^{13}C-NMR (DMSO-d_6) spectrum of [4-(4-hydroxyphenyl)piperazin-1-yl](4-methoxyphenyl)methanone **(23)**

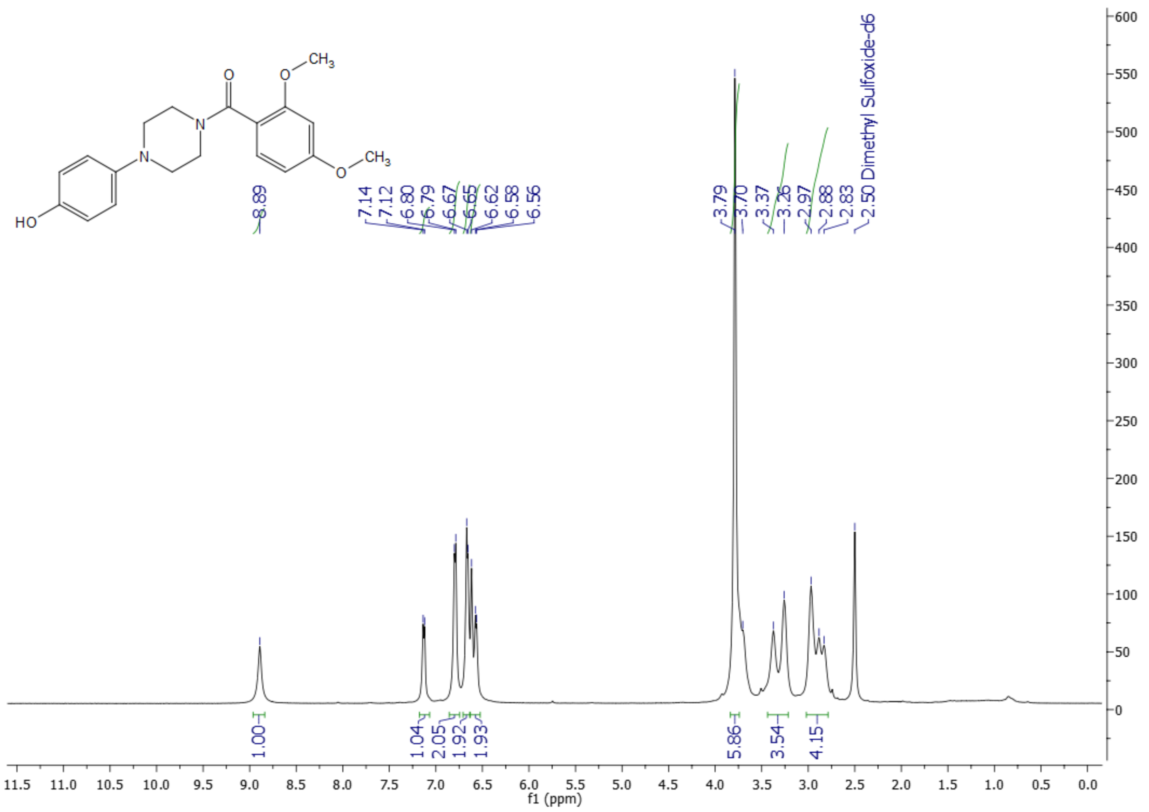

Figure S51: ^{1}H-NMR (DMSO-d_6) spectrum of [4-(4-hydroxyphenyl)piperazin-1-yl] 2,4-dimethoxyphenyl)methanone **(24)**

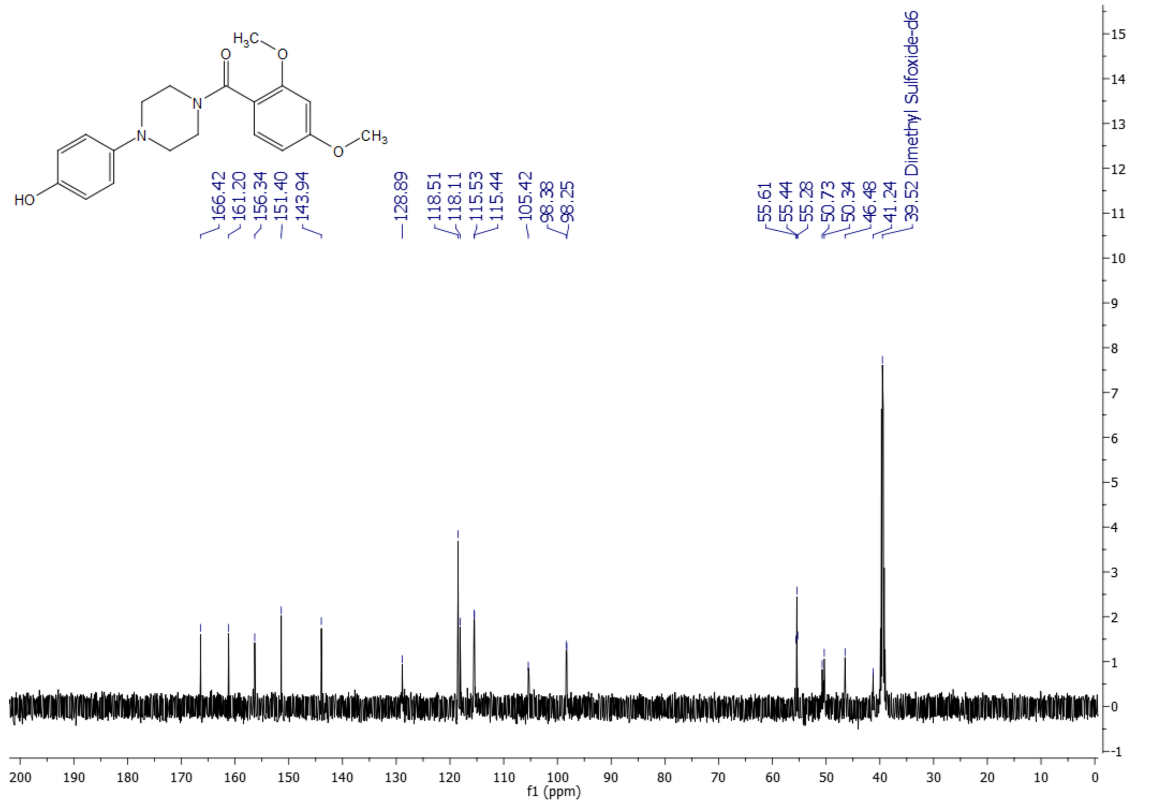

Figure S52: ^{13}C-NMR (DMSO-d_6) spectrum of 4-(4-hydroxyphenyl)piperazin-1-yl] 2,4-dimethoxyphenyl)methanone **(24)**

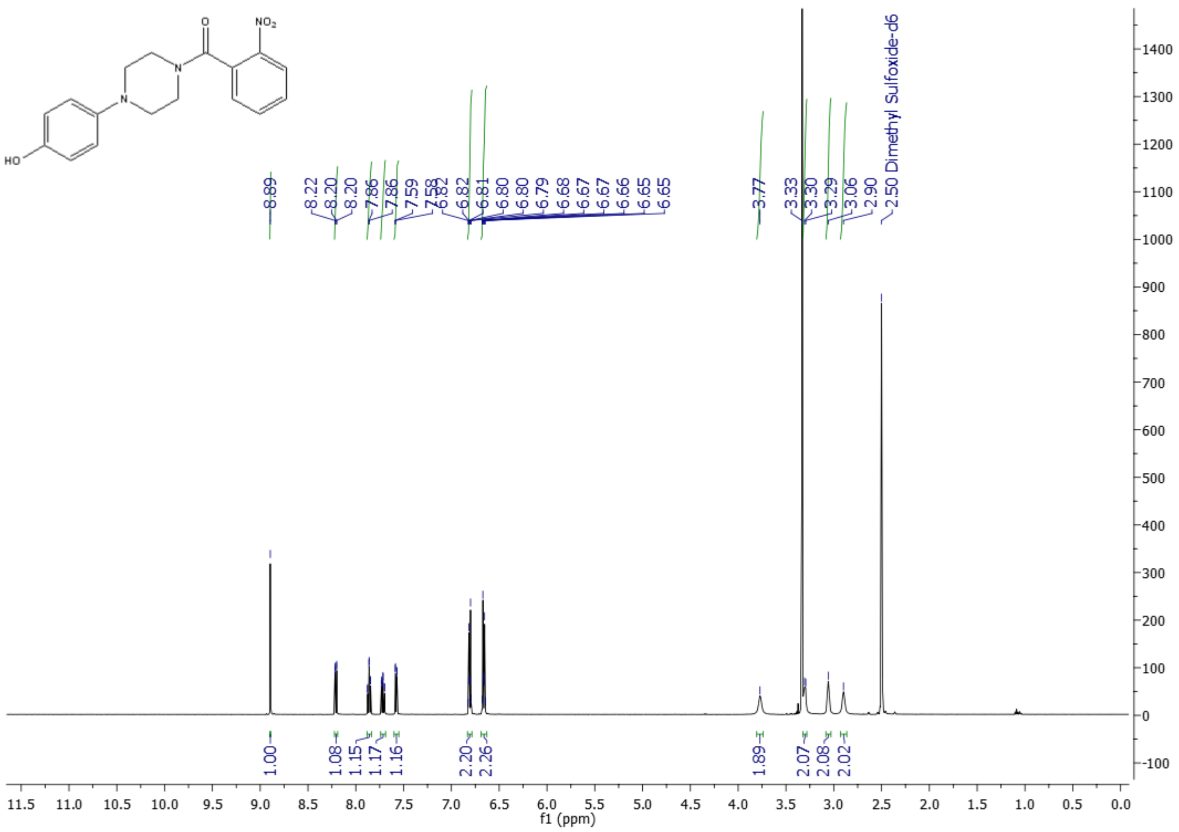

Figure S53: ^{1}H-NMR (DMSO-d$_6$) spectrum of (4-(4-hydroxyphenyl)piperazin-1-yl)(2-nitrophenyl)methanone **(28)**

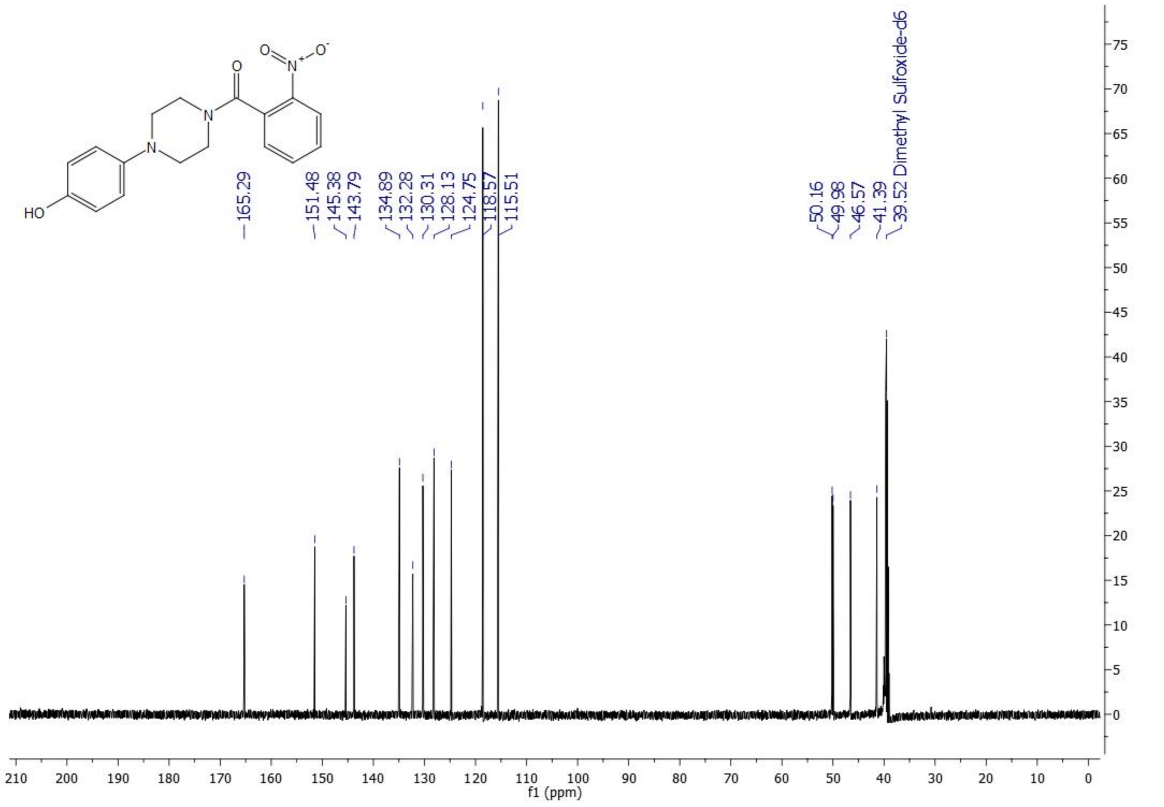

Figure S54: ^{13}C-NMR (DMSO-d$_6$) spectrum of (4-(4-hydroxyphenyl)piperazin-1-yl)(2-nitrophenyl)methanone **(28)**

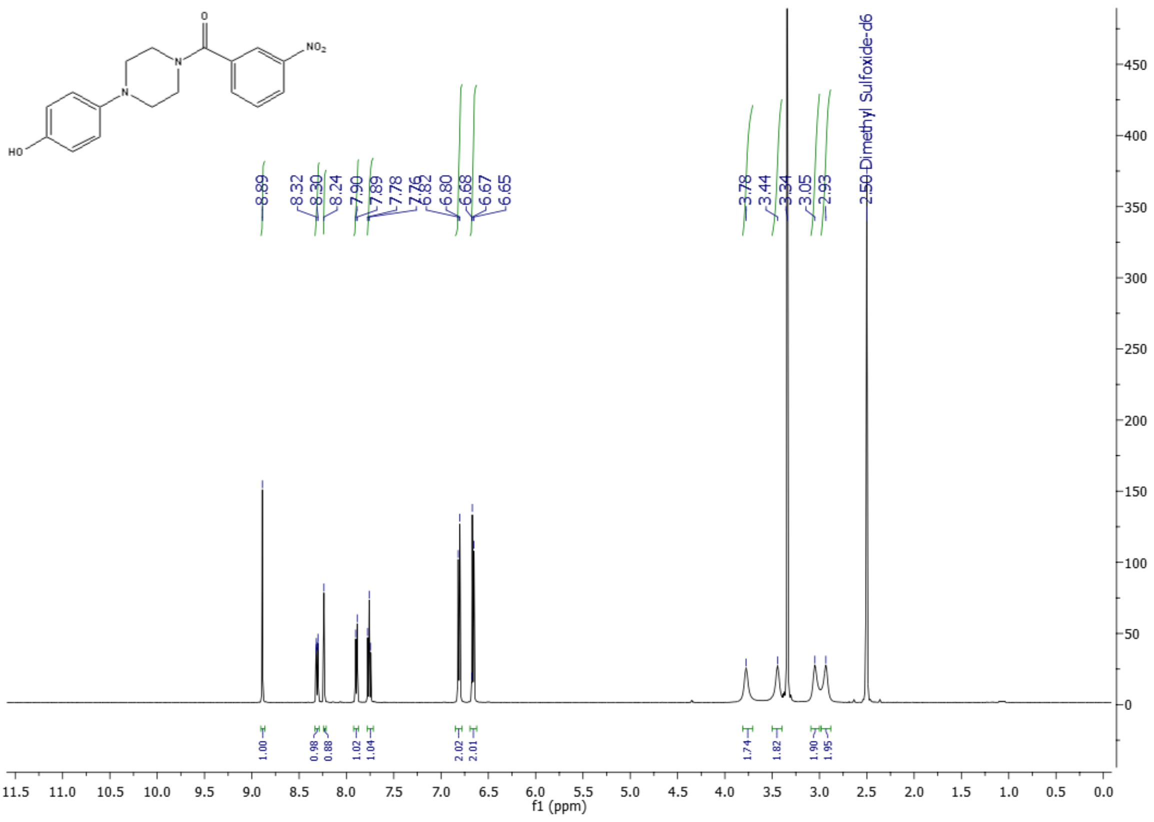

Figure S55: ^{1}H-NMR (DMSO-d_6) spectrum of (4-(4-hydroxyphenyl)piperazin-1-yl)(3-nitrophenyl)methanone **(29)**

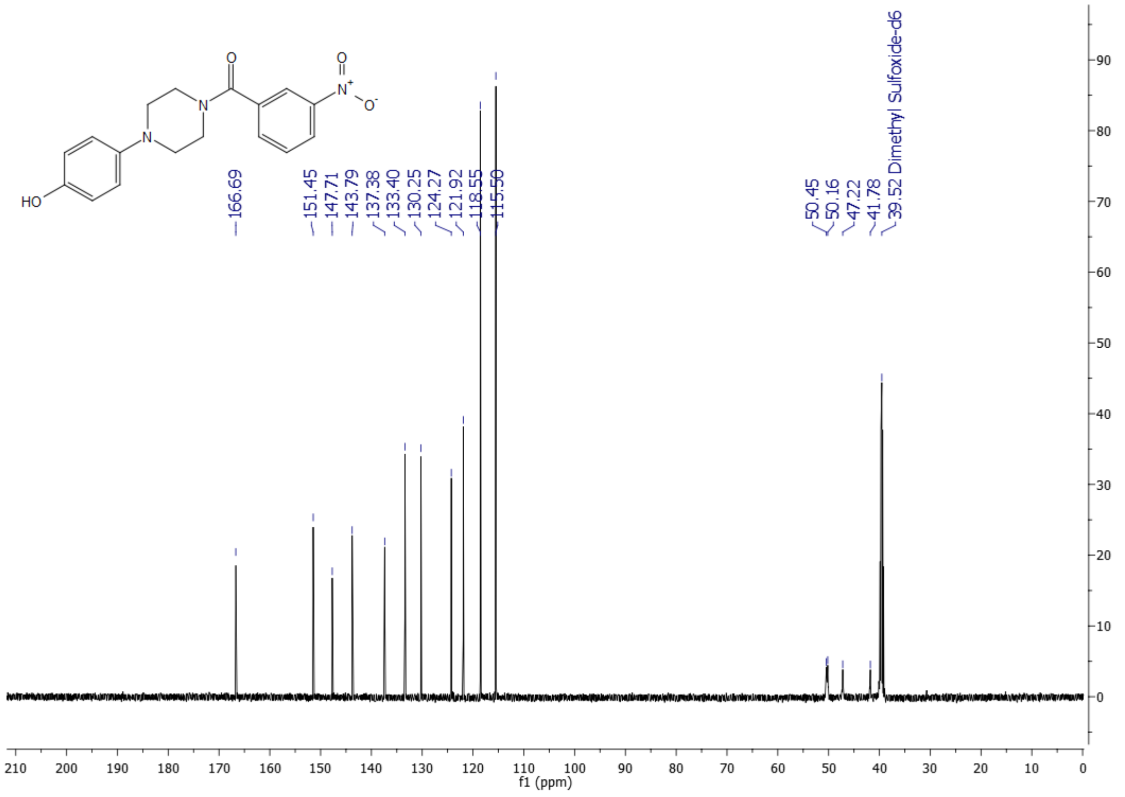

Figure S56: ^{13}C-NMR (DMSO-d_6) spectrum of (4-(4-hydroxyphenyl)piperazin-1-yl)(3-nitrophenyl)methanone **(29)**

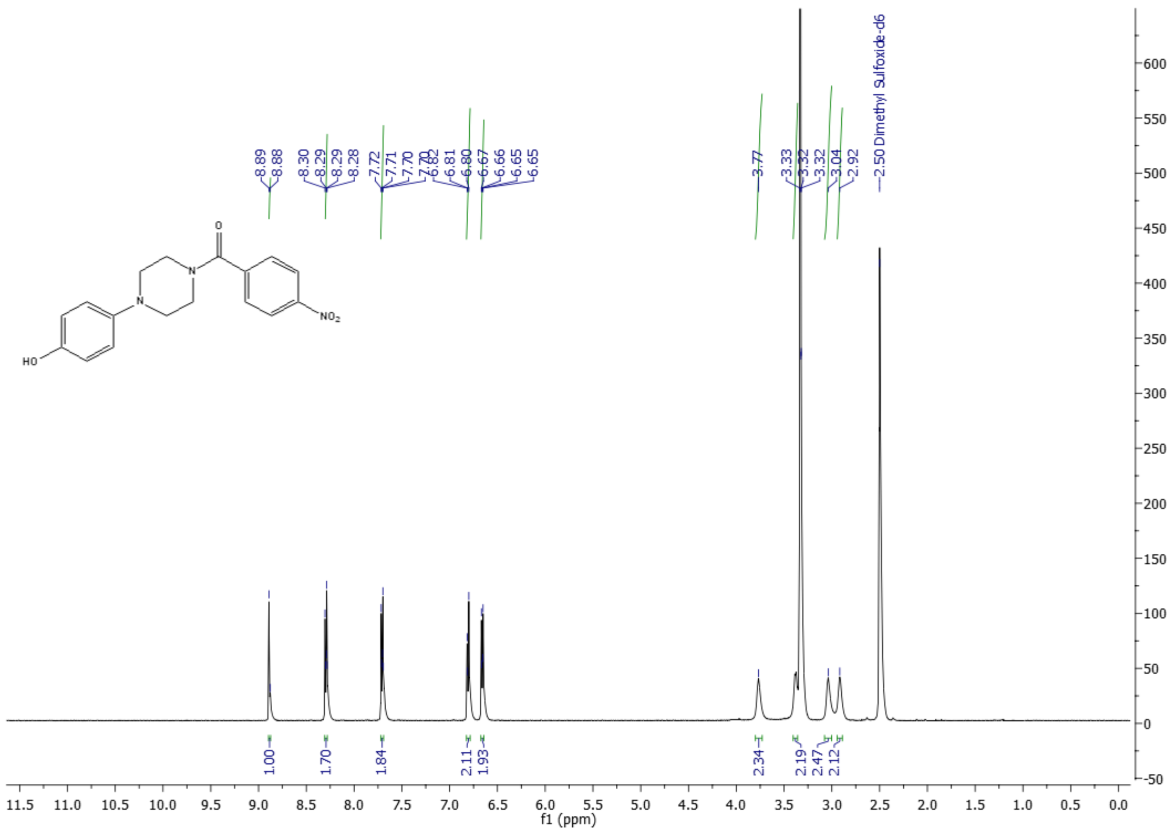

Figure S57: ^{1}H-NMR (DMSO-d_6) spectrum of (4-(4-hydroxyphenyl)piperazin-1-yl)(4-nitrophenyl)methanone **(30)**

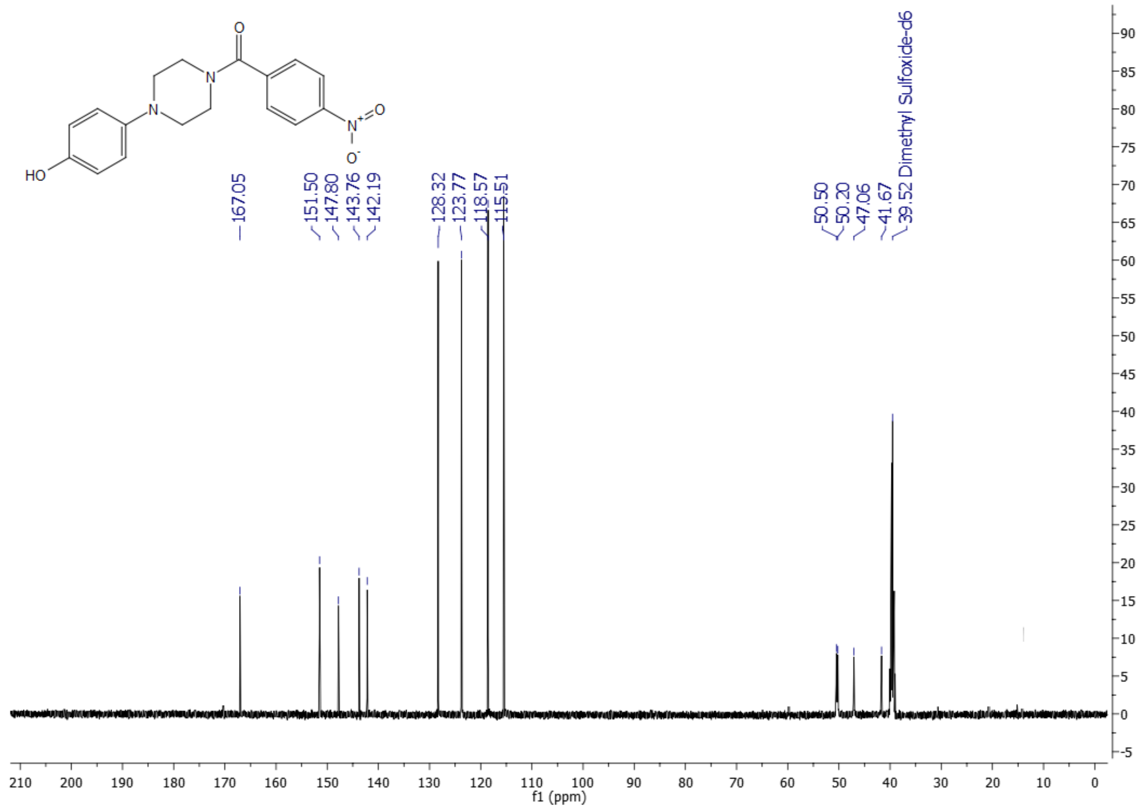

Figure S58: ^{13}C-NMR (DMSO-d_6) spectrum of (4-(4-hydroxyphenyl)piperazin-1-yl)(4-nitrophenyl)methanone **(30)**

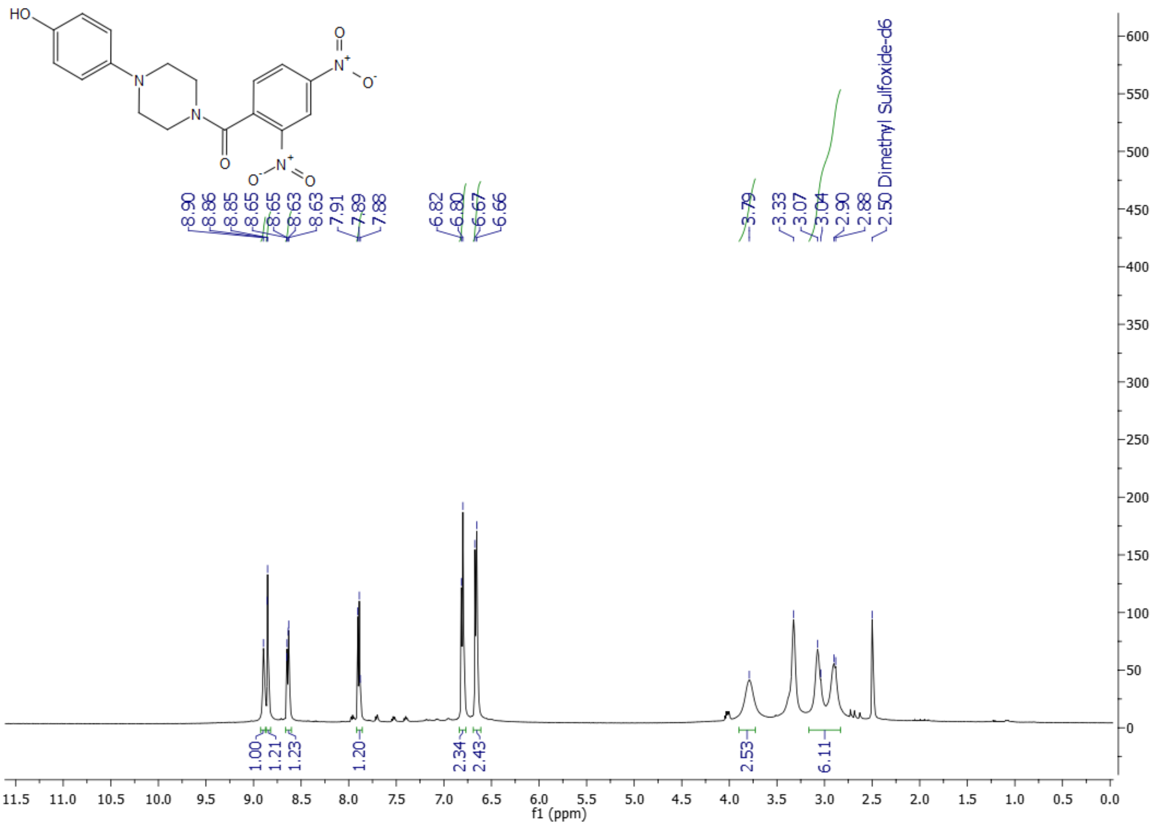

Figure S59: ^{1}H-NMR (DMSO-d_6) spectrum of (4-(4-hydroxyphenyl)piperazin-1-yl)(2,4-dinitrophenyl)methanone **(31)**

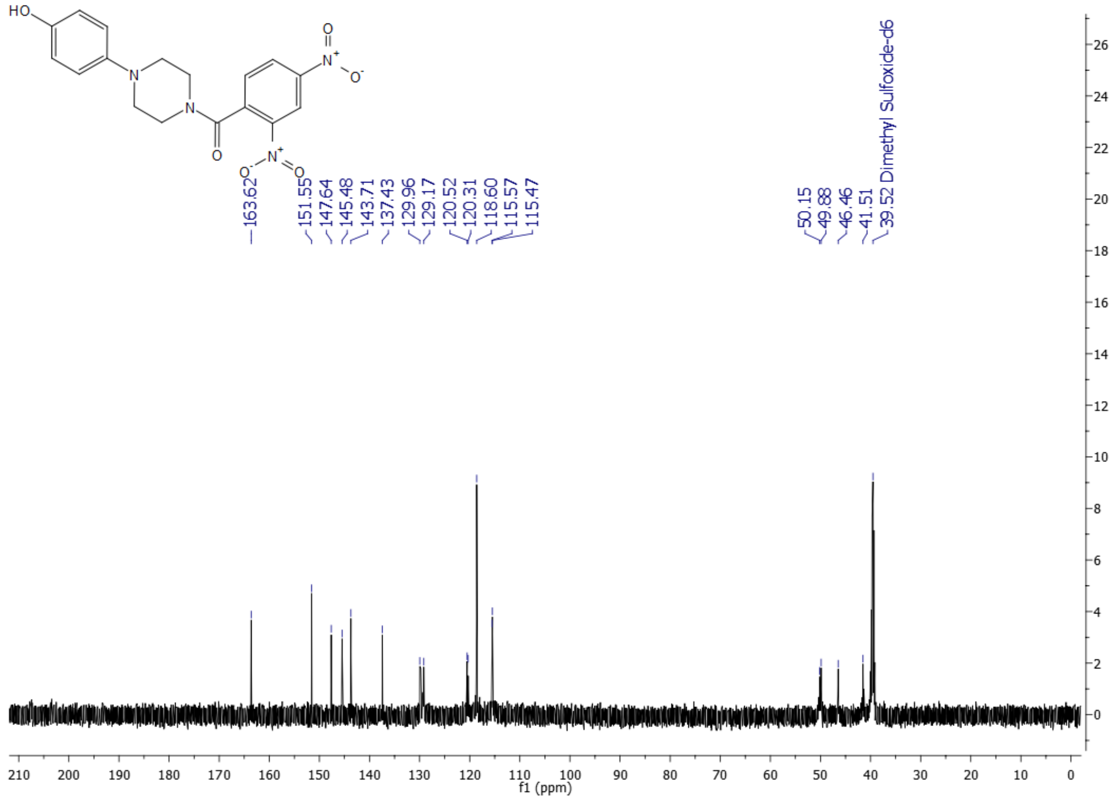

Figure S60: ^{13}C-NMR (DMSO-d_6) spectrum of (4-(4-hydroxyphenyl)piperazin-1-yl)(2,4-dinitrophenyl)methanone **(31)**

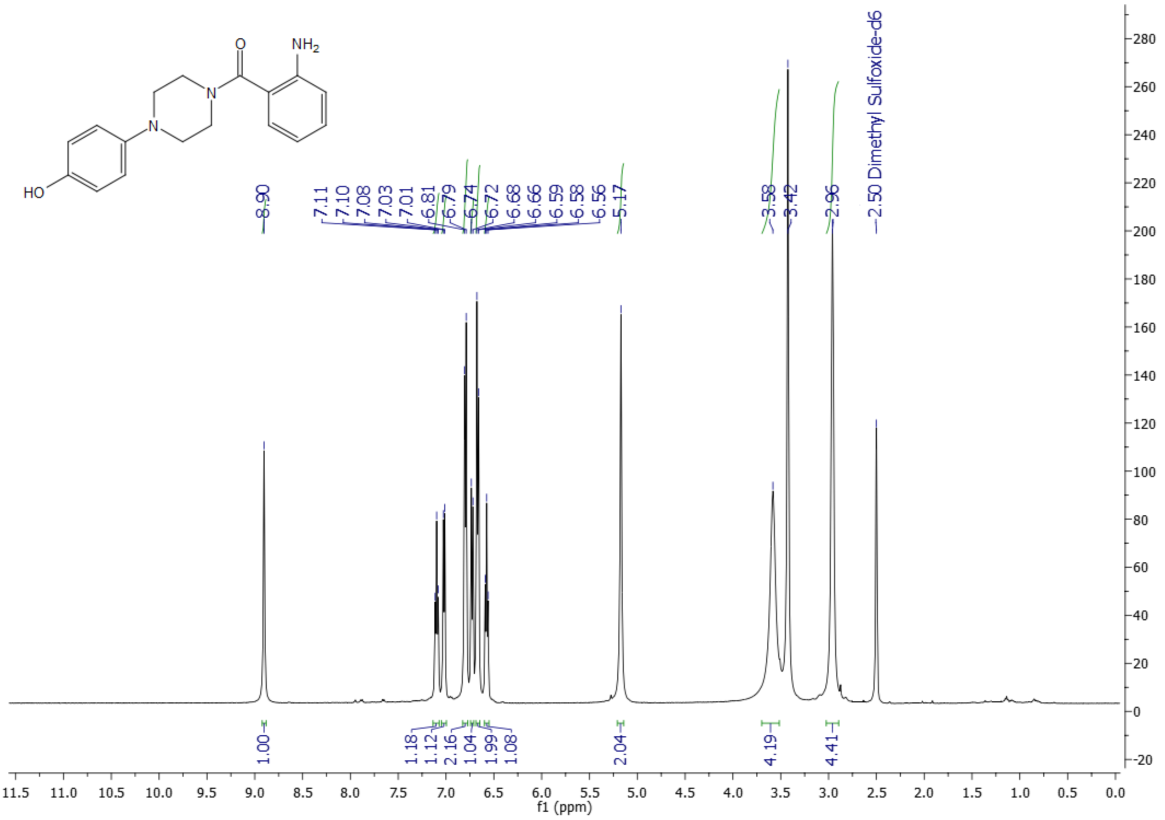

Figure S61: ^{1}H-NMR (DMSO-d_6) spectrum of (4-(4-hydroxyphenyl)piperazin-1-yl)(2-aminophenyl)methanone **(32)**

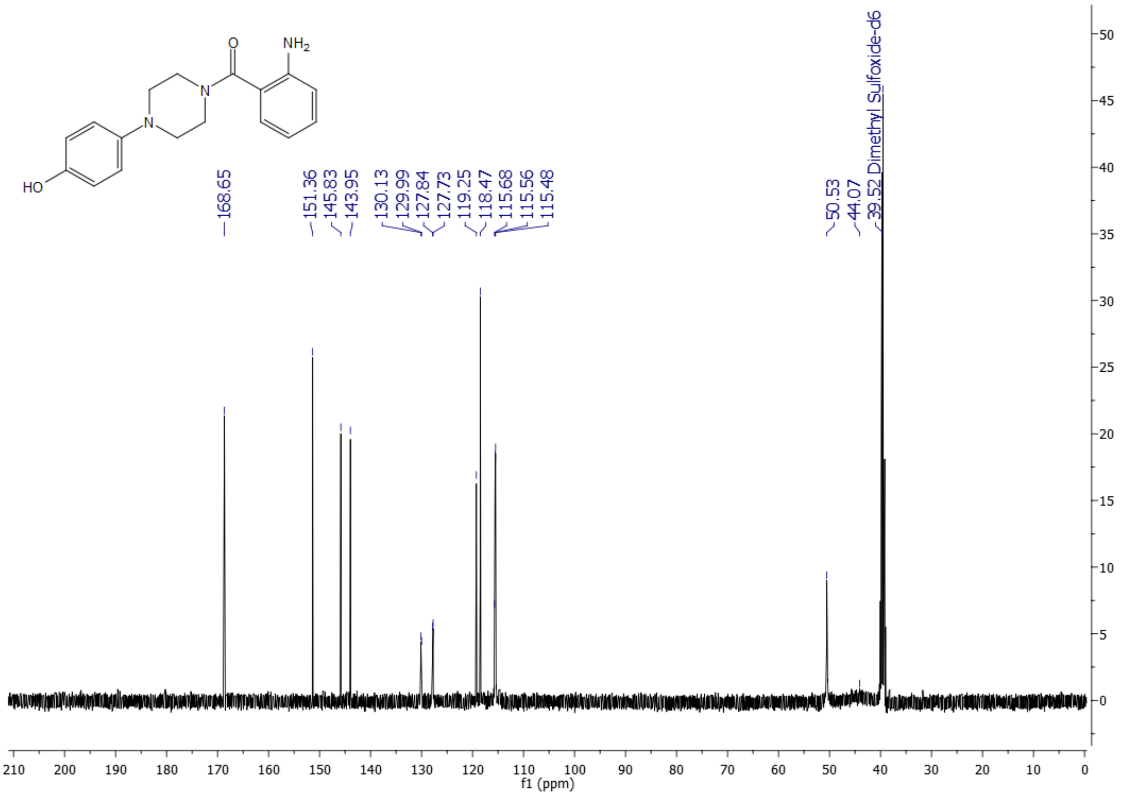

Figure S62: ^{13}C-NMR (DMSO-d_6) spectrum of 4-(4-hydroxyphenyl)piperazin-1-yl)(2-aminophenyl)methanone **(32)**

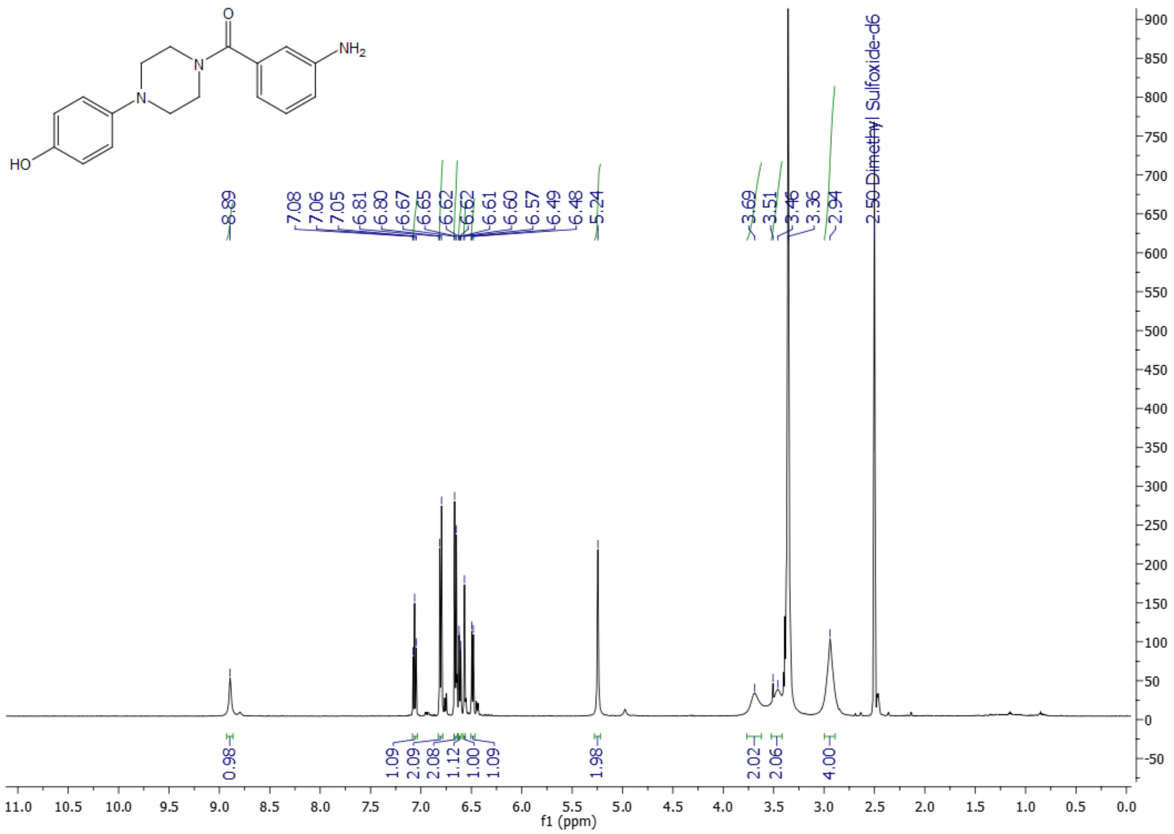

Figure S63: ^{1}H-NMR (DMSO-d_6) spectrum of (4-(4-hydroxyphenyl)piperazin-1-yl)(3-aminophenyl)methanone **(33)**

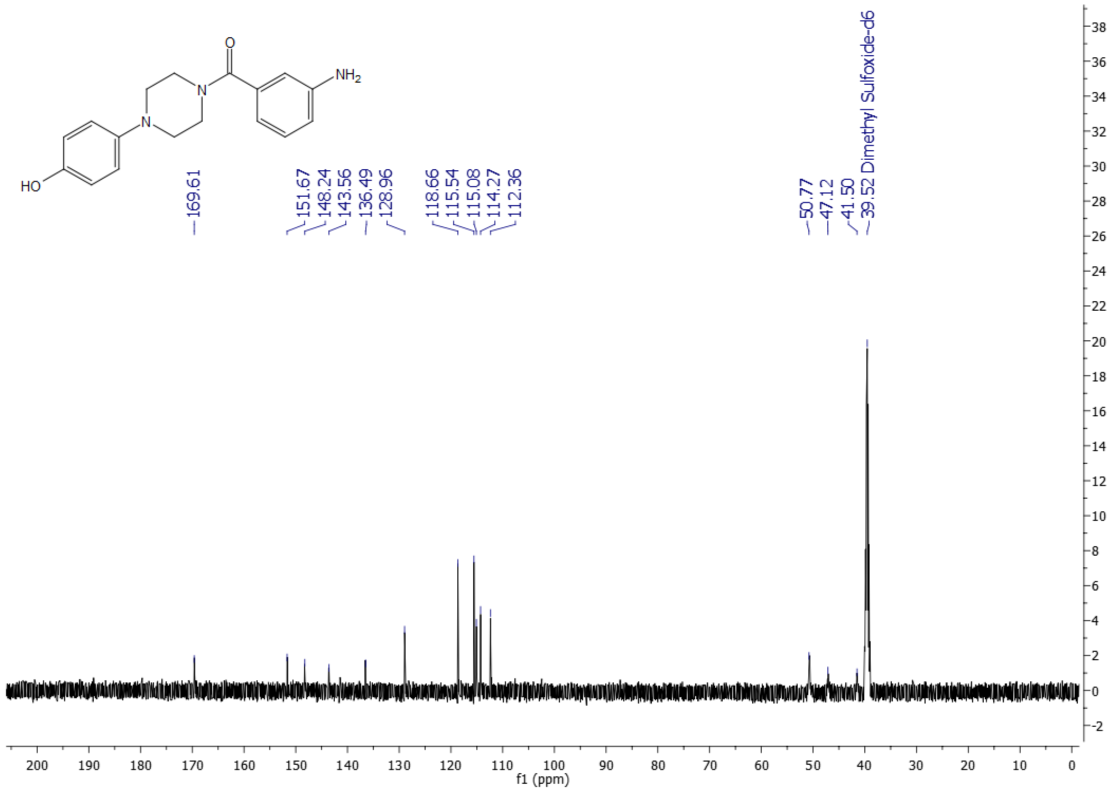

Figure S64: ^{13}C-NMR (DMSO-d_6) spectrum of (4-(4-hydroxyphenyl)piperazin-1-yl)(3-aminophenyl)methanone **(33)**

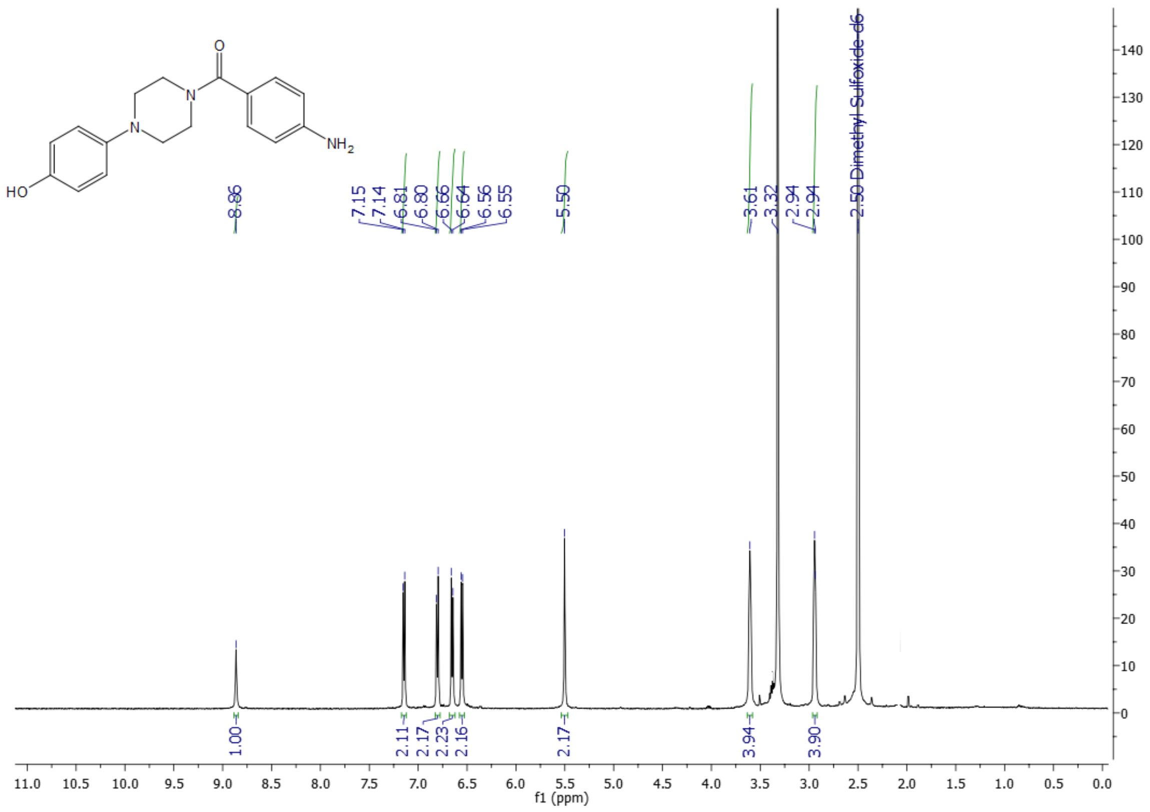

Figure S65: ^{1}H-NMR (DMSO-d_6) spectrum of (4-(4-hydroxyphenyl)piperazin-1-yl)(4-aminophenyl)methanone **(34)**

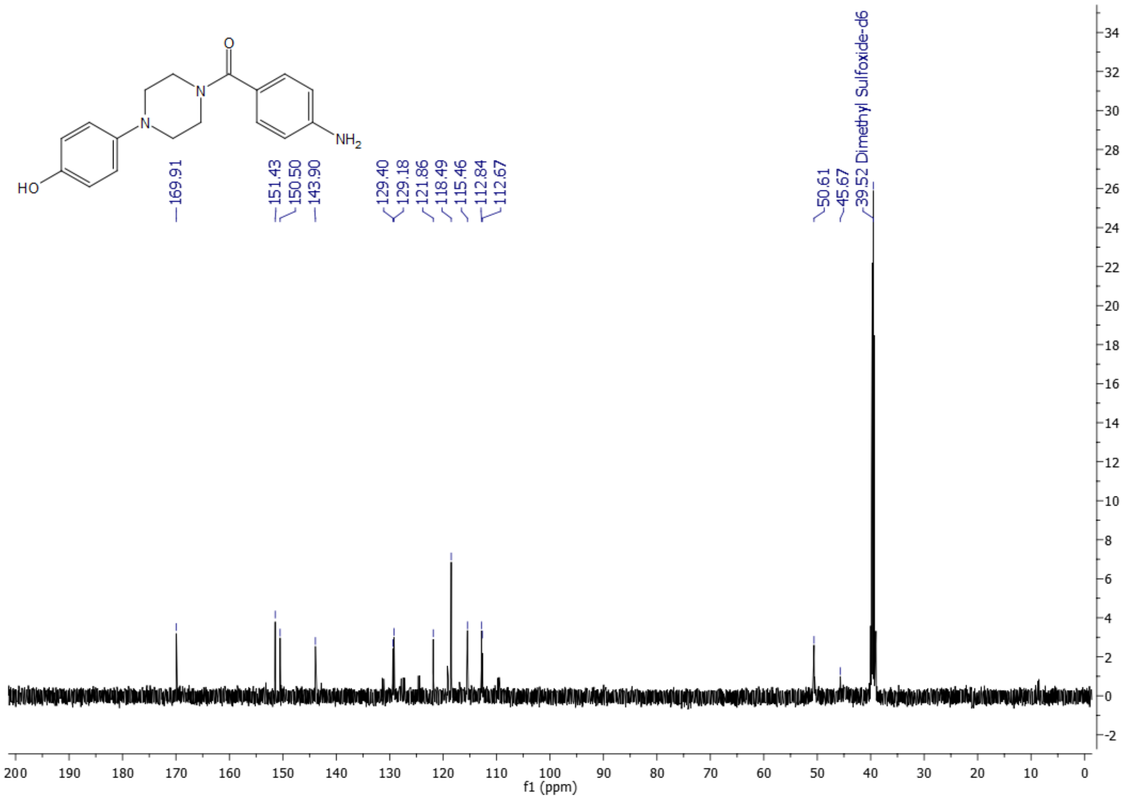

Figure S66: ^{13}C-NMR (DMSO-d_6) spectrum of 4-(4-hydroxyphenyl)piperazin-1-yl)(4-aminophenyl)methanone **(34)**

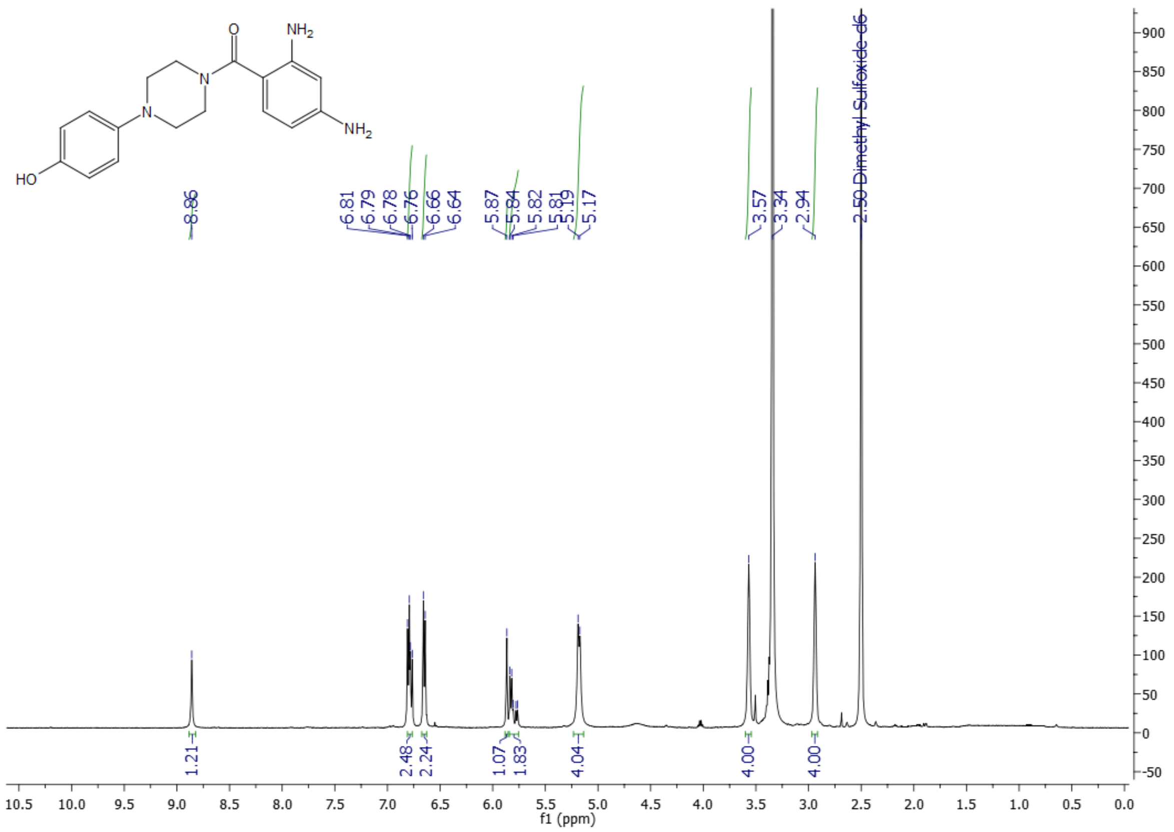

Figure S67: ^{1}H-NMR (DMSO-d_6) spectrum of (4-(4-hydroxyphenyl)piperazin-1-yl)(2,4-diaminophenyl)methanone **(35)**

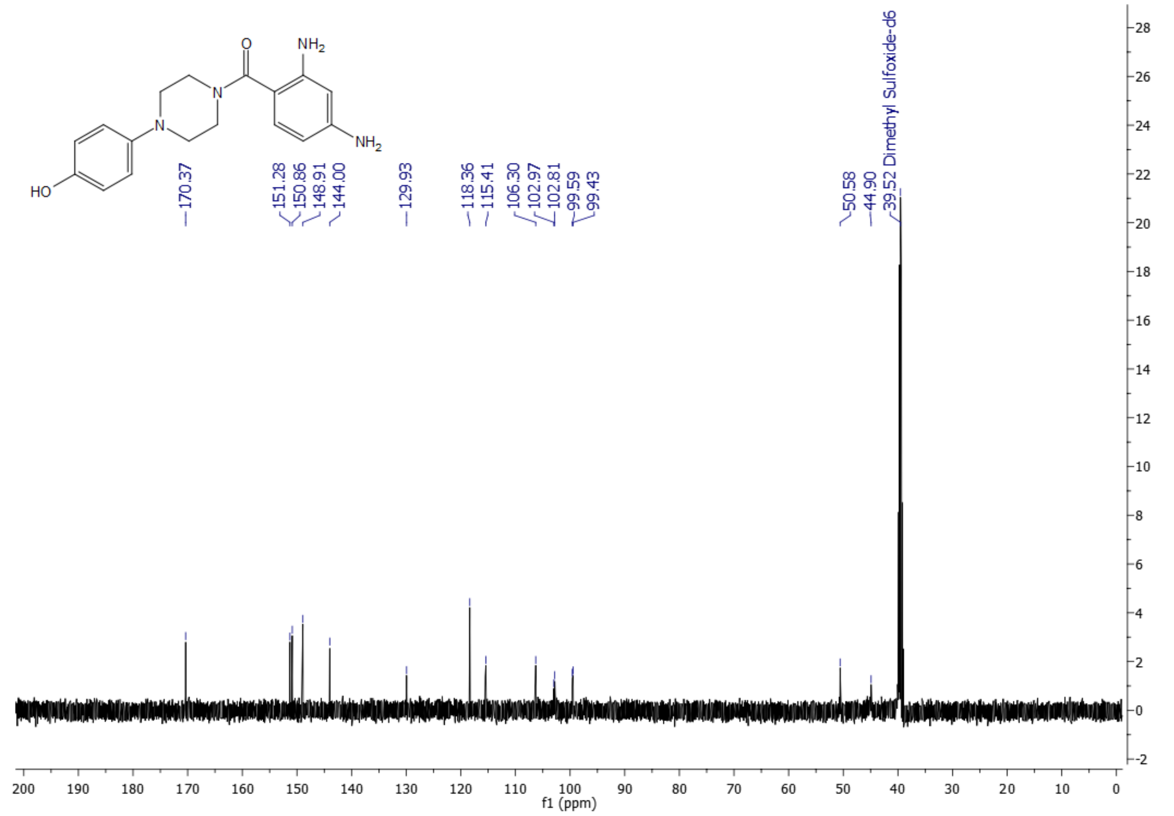

Figure S68: ^{13}C-NMR (DMSO-d_6) spectrum of (4-(4-hydroxyphenyl)piperazin-1-yl)(2,4-diaminophenyl)methanone **(35)**

Table S1: Smiles strings for compounds **2-35**

entry	SMILE
**2**	Oc1ccc(cc1)N2CCN(CC2)C(=O)c3ccccc3
**3**	Oc1ccc(cc1)N2CCN(CC2)C(=O)c3ccc(cc3)c4ccccc4
**4**	Oc1ccc(cc1)N2CCN(CC2)C(=O)c3ccccc3F
**5**	Oc1ccc(cc1)N2CCN(CC2)C(=O)c3cccc(F)c3
**6**	Oc1ccc(cc1)N2CCN(CC2)C(=O)c3ccc(F)cc3
**25**	Oc1ccc(cc1)N2CCN(CC2)C(=O)c3ccc(F)cc3F
**7**	Oc1ccc(cc1)N2CCN(CC2)C(=O)c3ccccc3Cl
**8**	Oc1ccc(cc1)N2CCN(CC2)C(=O)c3cccc(Cl)c3
**9**	Oc1ccc(cc1)N2CCN(CC2)C(=O)c3ccc(Cl)cc3
**10**	Oc1ccc(cc1)N2CCN(CC2)C(=O)c3ccc(Cl)cc3Cl
**11**	Oc1ccc(cc1)N2CCN(CC2)C(=O)c3ccccc3Br
**12**	Oc1ccc(cc1)N2CCN(CC2)C(=O)c3cccc(Br)c3
**13**	Oc1ccc(cc1)N2CCN(CC2)C(=O)c3ccc(Br)cc3
**26**	Oc1ccc(cc1)N2CCN(CC2)C(=O)c3ccc(Br)cc3Br
**14**	Cc1ccccc1C(=O)N2CCN(CC2)c3ccc(O)cc3
**15**	Cc1cccc(c1)C(=O)N2CCN(CC2)c3ccc(O)cc3
**16**	Cc1ccc(cc1)C(=O)N2CCN(CC2)c3ccc(O)cc3
**27**	Cc1ccc(C(=O)N2CCN(CC2)c3ccc(O)cc3)c(C)c1
**17**	Oc1ccc(cc1)N2CCN(CC2)C(=O)c3ccccc3C(F)(F)F
**18**	Oc1ccc(cc1)N2CCN(CC2)C(=O)c3cccc(c3)C(F)(F)F
**19**	Oc1ccc(cc1)N2CCN(CC2)C(=O)c3ccc(cc3)C(F)(F)F
**20**	Oc1ccc(cc1)N2CCN(CC2)C(=O)c3ccc(cc3C(F)(F)F)C(F)(F)F
**21**	COc1ccccc1C(=O)N2CCN(CC2)c3ccc(O)cc3
**22**	COc1cccc(c1)C(=O)N2CCN(CC2)c3ccc(O)cc3
**23**	COc1ccc(cc1)C(=O)N2CCN(CC2)c3ccc(O)cc3
**24**	COc1ccc(C(=O)N2CCN(CC2)c3ccc(O)cc3)c(OC)c1
**28**	Oc1ccc(cc1)N2CCN(CC2)C(=O)c3ccccc3[N+](=O)[O-]
**29**	Oc1ccc(cc1)N2CCN(CC2)C(=O)c3cccc(c3)[N+](=O)[O-]
**30**	Oc1ccc(cc1)N2CCN(CC2)C(=O)c3ccc(cc3)[N+](=O)[O-]
**31**	Oc1ccc(cc1)N2CCN(CC2)C(=O)c3ccc(cc3[N+](=O)[O-])[N+](=O)[O-]
**32**	Nc1ccccc1C(=O)N2CCN(CC2)c3ccc(O)cc3
**33**	Nc1cccc(c1)C(=O)N2CCN(CC2)c3ccc(O)cc3
**34**	Nc1ccc(cc1)C(=O)N2CCN(CC2)c3ccc(O)cc3
**35**	Nc1ccc(C(=O)N2CCN(CC2)c3ccc(O)cc3)c(N)c1

Mushroom tyrosinase inhibition assay

Aliquots (0.05mL) of test compound at various concentrations (0.5–140µM) were firstly mixed with 0.5 mL of L-tyrosine or L-DOPA solution (1.25mM), 0.9 mL of phosphate buffer (0.05M, pH 6.8). Then, the reaction mixture was preincubated at 25 °C for 10 min. Subsequently, 0.05 mL of an aqueous solution of mushroom tyrosinase (333 U/mL) was added. The linear increase in absorbance was measured after 60 or 1 min of incubation time in the reaction mixture containing L-tyrosine or L-DOPA, respectively. The inhibitory activity was expressed as inhibition percentage respect to control sample. The concentrations leading to 50 % activity loss (IC_{50}) were also calculated by interpolation of the dose-response curves. Kojic acid [5-hydroxy-2-(hydroxymethyl)-4H-pyran-4-one], a fungal secondary metabolite used as skin whitening agent, was employed as a positive standard (10–50 µM). A spectrophotometer (Agilent Cary 60 UV-Vis Spectrophotometer) was used for absorbance measurements.

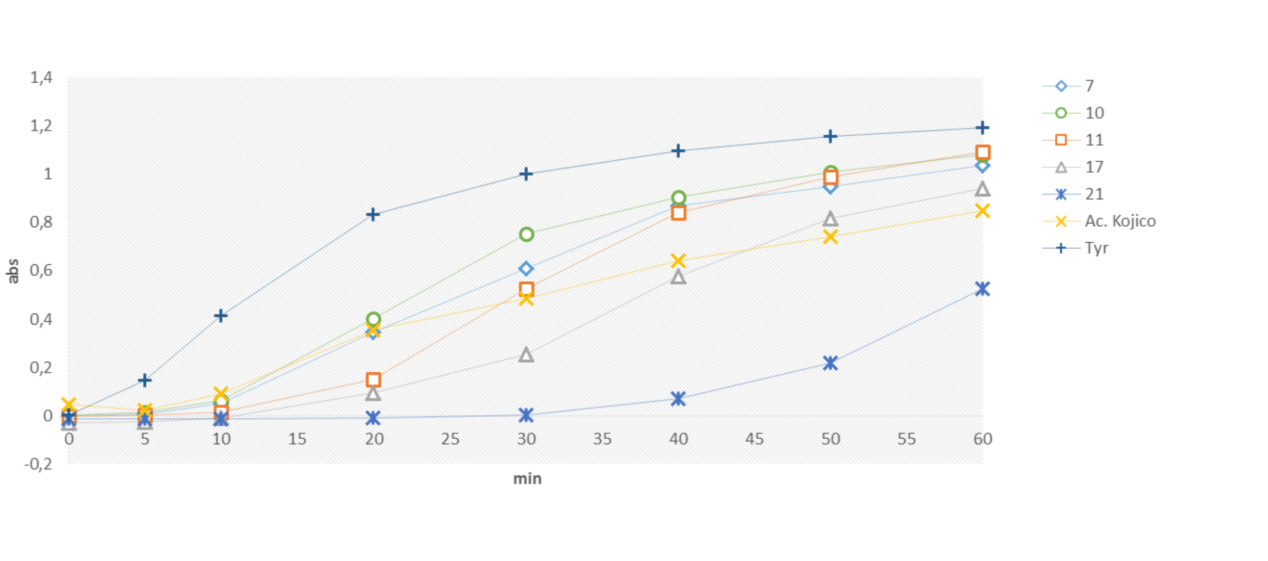

Figure S69: Inhibition effects of compounds **7, 10 11, 17** and **21** (40 µM) on monophenolase activity of AbTYR compared to **Kojic Acid**

Kinetic analysis of the tyrosinase inhibition

The inhibition kinetics on the tyrosinase were studied using Lineweaver-Burk double reciprocal plots. The reaction mixture consisted of four different concentrations of the substrate L-DOPA (0.6–5 mM), and mushroom tyrosinase in phosphate buffer (0.05 M, pH 6.8). Three different concentrations were used for the analysis. Preincubation and measurement time were the same as discussed in mushroom tyrosinase inhibition assay. The Michaelis-Menten constant (Km) and maximal velocity (Vmax) of tyrosinase were determined by Lineweaver-Burk plots.

A

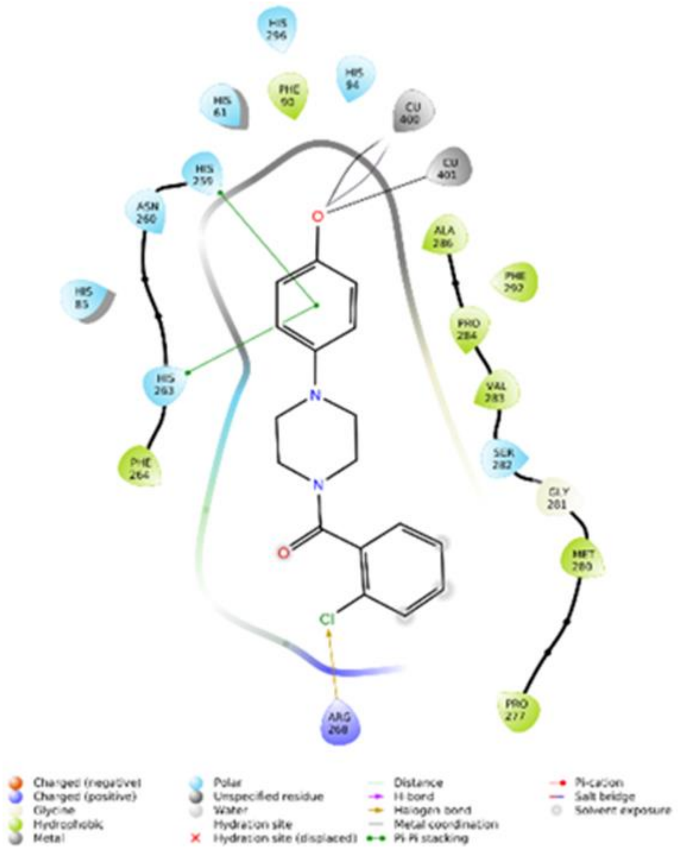

HIS 296
HIS 94
PHE 90
HIS 61
CU 400
CU 401
HIS 259
ASN 260
HIS 85
HIS 263
PHE 264
ALA 286
PHE 292
PRO 284
VAL 283
SER 282
GLY 281
MET 280
PRO 277
ARG 268
O
N
N
O
Cl
Charged (negative)
Charged (positive)
Glycine
Hydrophobic
Metal
Polar
Unspecified residue
Water
Hydration site
Hydration site (displaced)
Distance
H-bond
Halogen bond
Metal coordination
Pi-Pi stacking
Pi-cation
Salt bridge
Solvent exposure

B

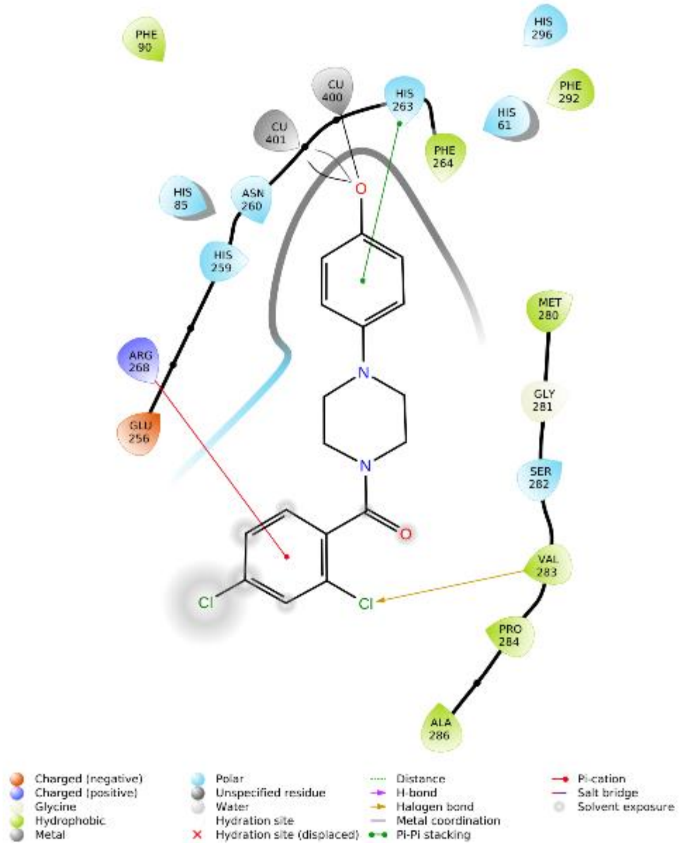

PHE 90
HIS 296
CU 400
HIS 263
PHE 292
CU 401
HIS 61
PHE 264
HIS 85
ASN 260
HIS 259
MET 280
ARG 268
GLY 281
GLU 256
SER 282
VAL 283
PRO 284
ALA 286
O
N
N
O
Cl
Cl
Charged (negative)
Charged (positive)
Glycine
Hydrophobic
Metal
Polar
Unspecified residue
Water
Hydration site
Hydration site (displaced)
Distance
H-bond
Halogen bond
Metal coordination
Pi-Pi stacking
Pi-cation
Salt bridge
Solvent exposure

C

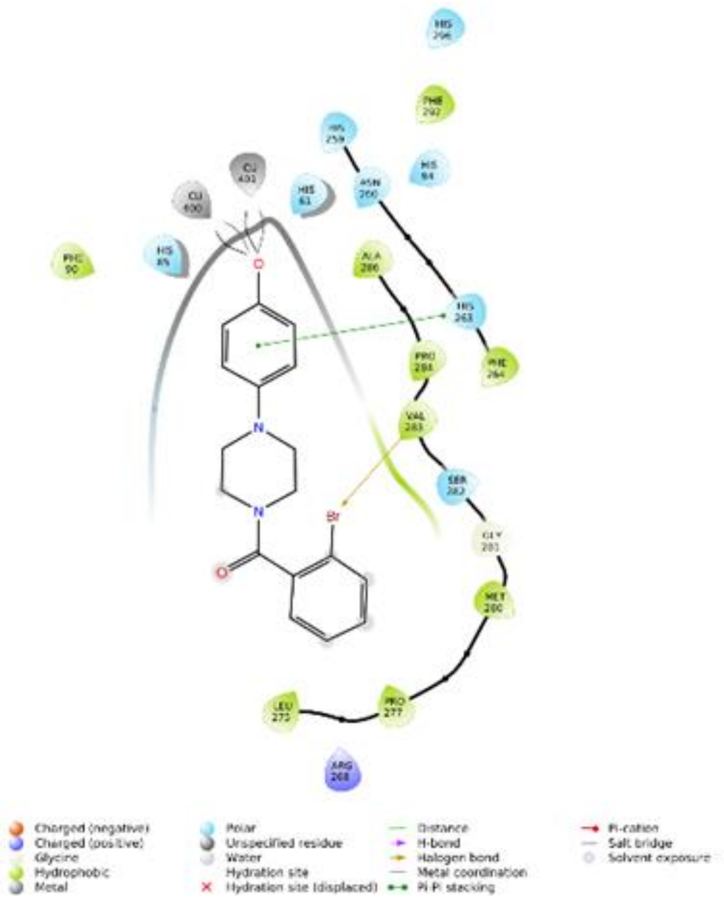
O
N
N
Br
O
Charged (negative)
Charged (positive)
Glycine
Hydrophobic
Metal
Polar
Unspecified residue
Water
Hydration site
Hydration site (displaced)
Distance
H-bond
Halogen bond
Metal coordination
Pi-Pi stacking
Pi-cation
Salt bridge
Solvent exposure

D

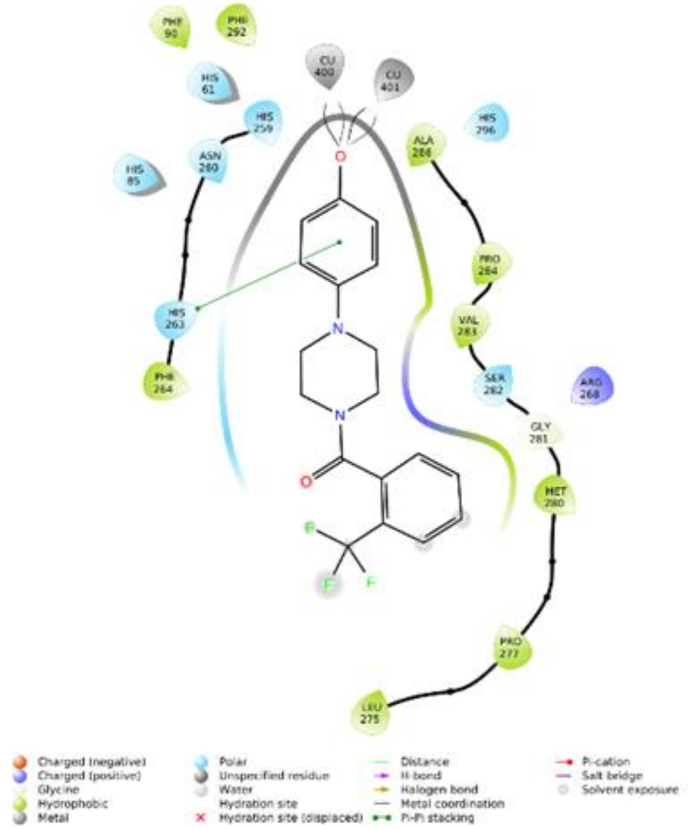
O
N
N
O
F
F
F
Charged (negative)
Charged (positive)
Glycine
Hydrophobic
Metal
Polar
Unspecified residue
Water
Hydration site
Hydration site (displaced)
Distance
H-bond
Halogen bond
Metal coordination
Pi-Pi stacking
Pi-cation
Salt bridge
Solvent exposure

E

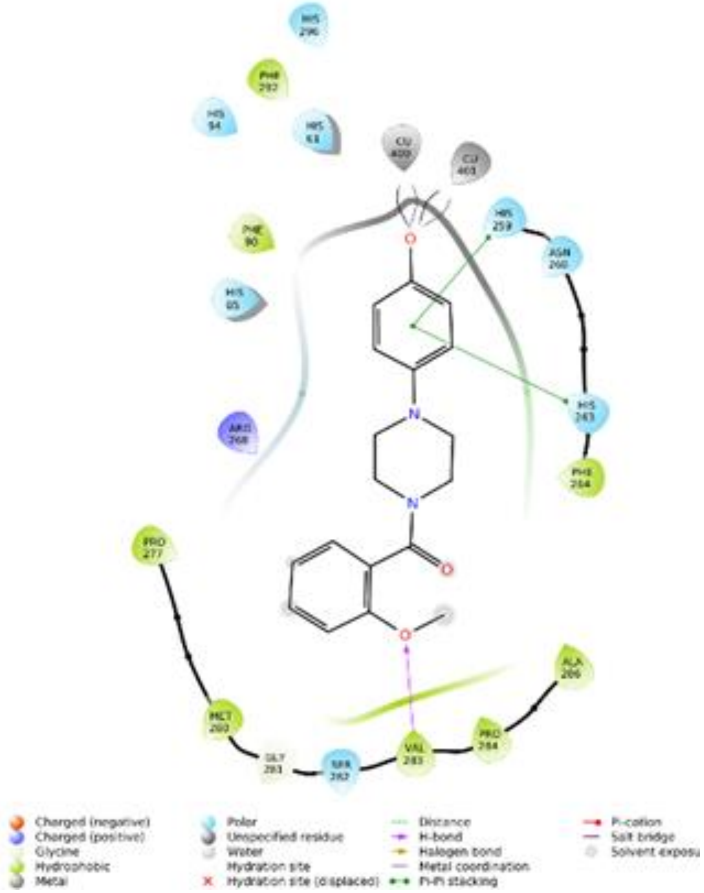

Figure S70: 2 D interaction diagrams of compounds **7** (A),**10** (B),**11** (C),**17** (D),**21** (E) in the catalytic cavity of AbTYR

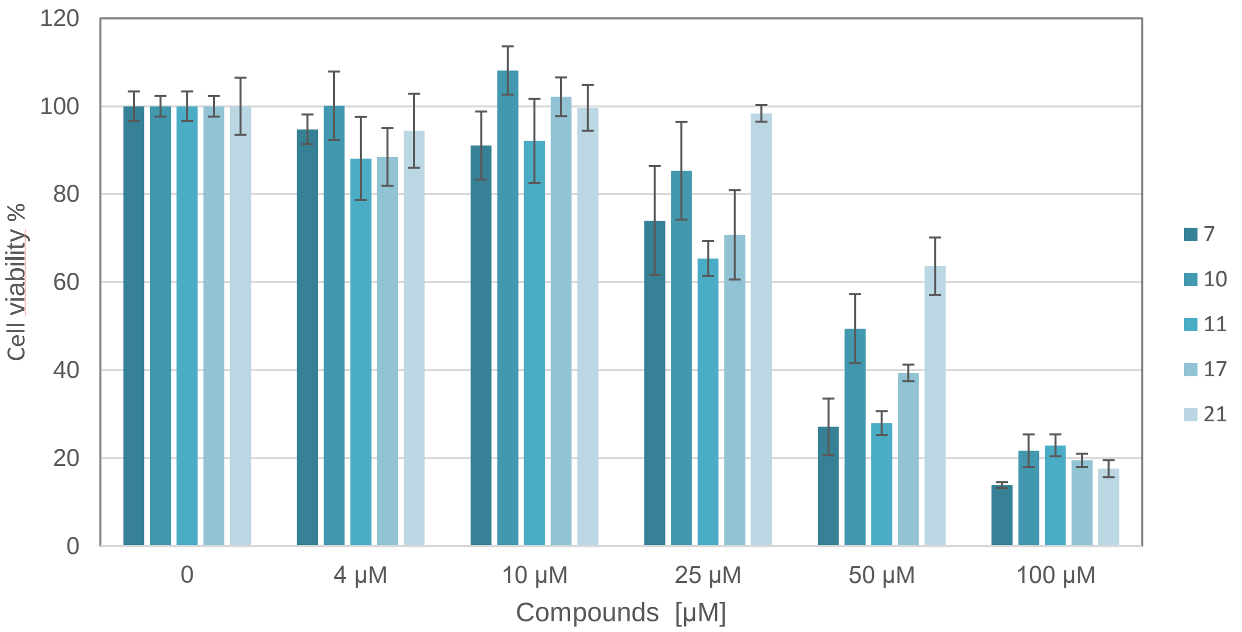

Figure S71: Effect of compounds **7**, **10**, **11**, **17** and **21** on B16F10 cell viability. Cells were treated with different concentrations of compounds (4-100 µM) and their viability was evaluated by MTT assay. Data represent mean ± SD of triplicate experiments.
